# Global, regional, and national burden of atrial fibrillation/flutter attributable to metabolic, behavioral, and environmental risk factors, 1990–2021: a longitudinal observational study

**DOI:** 10.3389/fnut.2025.1560334

**Published:** 2025-05-15

**Authors:** Zhen Wei, Qi Wang, Hong-Ling Jia

**Affiliations:** ^1^School of Acupuncture and Tuina, Shandong University of Traditional Chinese Medicine, Jinan, China; ^2^Department of Acupuncture, Second Affiliated Hospital of Shandong University of Traditional Chinese Medicine, Jinan, China

**Keywords:** atrial fibrillation/flutter, risk factors, age-standardized mortality rates (ASMR), age-standardized disability-adjusted life year rates (ASDR), average annual percentage change (AAPC)

## Abstract

**Background:**

Atrial fibrillation/flutter (AF/AFL) remains a significant global public health issue, with its development influenced by metabolic, behavioral, and environmental risk factors However, comprehensive analyses of temporal and geographic variations in AF/AFL burden attributed to risk factors remain lacking.

**Objective:**

This study reveals the geographic and temporal distribution of the burden of AF/AFL attributable to specific risk factors at global, regional, and national levels from 1990 to 2021, providing a scientific basis for developing targeted prevention and control policies.

**Methods:**

We extracted data on AF/AFL risk-attributable deaths, disability-adjusted life years (DALYs), age-standardized mortality rates (ASMRs), and age-standardized DALY rates (ASDRs) from the Global Burden of Disease (GBD) database for the years 1990 to 2021. The burden of AF/AFL caused by metabolic, behavioral, and environmental risk factors stratified by age, sex, region, and country. Long-term trends in the AF/AFL burden associated with specific risk factors were assessed using the average annual percentage change (AAPC).

**Results:**

Over the past 32 years, high body mass index has been the primary contributor to the rising AF/AFL burden, with AAPCs of ASMR and ASDR at 1.66 (95% CI: 1.63–1.68) and 1.68 (95% CI: 1.67–1.70), respectively. The most significant increase occurred in males aged 30–34. The burden related to high sodium diets and lead exposure is also rising, particularly in females aged 65–69. Smoking showed the greatest decrease, with AAPCs of ASMR and ASDR at −0.66 (95% CI: −0.68 to −0.65) and −0.72 (95% CI: −0.72 to −0.71), most significantly in women aged 30–34. High systolic blood pressure decreased overall, but increased in individuals aged 34–49. East Asia saw the largest increase in burden from high body mass index, with AAPCs of ASMR at 8.28 (95% CI: 8.18–8.37) and ASDR at 8.22 (95% CI: 8.18–8.26). In 2021, China had the highest AF/AFL deaths and DALYs attributed to high systolic blood pressure, high sodium diets, smoking, and lead exposure.

**Conclusion:**

From 1990 to 2021, high body mass index became the primary driver of the rising global AF/AFL burden, particularly affecting East Asia and young and middle-aged adults. In contrast, the burden associated with smoking showed the greatest decline. In 2021, China had the highest AF/AFL burden due to various risk factors. Given the regional variations and characteristics of high-risk populations, policymakers should develop targeted yet comprehensive prevention strategies. These measures should include promoting healthy dietary habits, strengthening global surveillance systems, and fostering data-sharing collaborations to mitigate this growing epidemic.

## Introduction

Atrial fibrillation/Atrial flutter (AF/AFL) is the most common type of tachyarrhythmia worldwide. As the global population ages, the prevalence of AF/AFL continues to rise annually. This condition is associated with severe complications, including myocardial infarction, heart failure, and stroke, which markedly elevate the risk of mortality (1). In 2021, AF/AFL was responsible for over 330,000 deaths and 8.35 million disability-adjusted life years (DALYs), making it one of the leading global causes of death and disability, significantly impacting patients’ quality of life. The Global Burden of Disease Study 2021 reports that the number of AF/AFL-related deaths and DALYs is increasing at a faster rate than both prevalent and incident cases (2). Therefore, early detection and effective prevention of AF/AFL are critical to reducing its prevalence and associated complications, presenting major public health and clinical challenges, particularly in low-income countries. Additionally, the burden of AF /AFL varies significantly by geographic region, particularly in high-income areas of Europe and North America, where both incidence and prevalence are among the highest globally (3–5). The unequal burden of cardiovascular disease across countries, regions, genders, and age groups is reflected in the United Nations Sustainable Development Goal 3.4.1, which aims to reduce premature deaths from non-communicable diseases (e.g., cardiovascular disease) by one-third by 2030, through addressing changing risk factors and implementing effective healthcare interventions (6).

AF/AFL is classified into two categories: non-modifiable (e.g., age, ethnicity) and modifiable (e.g., high blood pressure, high body-mass index, smoking, alcohol use, high-sodium diets, lead exposure) categories (7). Studies suggest that AF/AFL risk can be reduced by managing modifiable factors. For example, high systolic blood pressure increases the risk of AF/AFL by 19% for every 10 mmHg increase, while a high body-mass index increases the risk by 4–5% for each unit increase (2, 8, 9). Therefore, controlling these risk factors can help reduce the incidence of AF/AFL. Additionally, there are significant gender differences in risk factors, with a higher incidence in men and a higher mortality rate in women (10). However, existing studies have primarily focused on assessing the global burden of AF/AFL or examining the impact of risk factors on AF/AFL burden within a single geographic context (1). There remains a lack of comprehensive evaluations that simultaneously consider metabolic, behavioral, and environmental risk factors across different time periods, age and sex groups, as well as regions with varying socioeconomic structures and levels of national development. This gap in research not only limits a comprehensive understanding of AF/AFL burden but also hinders the optimization of prevention strategies and the refinement of treatment priorities. To address this limitation, this study conducts the first longitudinal analysis to systematically assess the temporal trends in AF/AFL burden attributable to specific risk factors at the global, regional, and national levels from 1990 to 2021, with stratification by sex and age. The findings provide critical evidence for different countries and regions, facilitating the development of more targeted AF/AFL prevention and control strategies to effectively reduce the global disease burden.

The Global Burden of Disease (GBD) database assesses the burden of AF/AFL by extensively collecting data on modifiable risk factors from populations of different ages and sexes across various regions and countries. GBD quantifies and models the impact of these risk factors on AF/AFL across multiple temporal and geographic dimensions, with a rigorous quality assurance process in place. Furthermore, GBD 2021 provides up-to-date data on risk-outcome pairs from 1990 to 2021, covering 204 countries and territories, enabling a comprehensive evaluation of the risk-attributable disease burden across nations. In this study, we analyzed the burden of AF/AFL attributable to metabolic, behavioral and environmental factors at global, regional, and national levels. This study aims to provide targeted prevention and control evidence for reducing the burden of AF/AFL in different countries and regions.

## Methods

### Study design and data source

All data for this study were obtained from the Global Burden of Disease, Injury, and Risk 2021 (GBD 2021) database, which rigorously evaluates 371 diseases, injuries, and 88 risk factors across geographic and temporal distributions, as well as by age and gender, using the most recent epidemiological data and standardized assessment methods (11, 12). The GBD 2021 database divides 204 countries and territories into 21 regions based on geography and economic development levels. It also categorizes these countries and regions into five socio-economic index (SDI) regions based on fertility, per capita income, and educational attainment: low SDI (0–0.455), medium-low SDI (0.456–0.608), medium SDI (0.609–0.690), medium-high SDI (0.690–0.805), and high SDI (0.806–1). The SDI index ranges from 0 to 1, with a higher value indicating a higher level of health development (13, 14). In this study, we used age-standardized rates (ASR) to quantify the mortality and disability-adjusted life-year (DALY) rates of AF/AFL burden associated with specific risk factors. ASR eliminates the effect of population age structure on overall rates, allowing comparisons of the disease burden across different ages and regions.

We collected data on deaths, DALYs, age-standardized mortality rate (ASMR), and age-standardized DALY rate (ASDR) related to AF/AFL from GBD 2021 database, covering global, regional, and national levels from 1990 to 2021. AF/AFL cases were identified using the International Classification of Diseases (ICD) codes: ICD-9 (427.3) and ICD-10 (I48–I48.9). The data were stratified by sex and age. To account for uncertainty due to location-specific, time period-specific, and heterogeneous data, we extracted a 95% uncertainty interval (UI) for each dataset. The 95% UI for each data point was calculated by ranking the 1,000 sampled values and taking the 25th and 975th percentiles (15). It is important to note that the 95% UI for risk-attributable deaths or DALYs may be negative. This is due to the Burden of Proof Risk Function (BPRF) method used in GBD 2021, which integrates between-study heterogeneity, resulting in wider 95% intervals. The inclusion of negative values reflects poorly understood or weak risk–outcome relationships. We report the full uncertainty distribution for transparency. The presence of negative values in the 95% UI merely reflects statistical uncertainty rather than actual effects. If the core attributable value remains positive, it still supports the public health significance of the risk factor in disease burden (11). Furthermore, epidemiological studies indicate that AF/AFL is more common in individuals over 30, with negligible incidence and mortality in those under 30 (16). Therefore, this study defines the 30–34 age group as the starting category, with subsequent groups spanning five-year intervals, and those over 95 as the final group. This classification follows the age-standardized grouping used in previous GBD analyses, ensuring comparability across different age populations globally (1, 2). All data utilized in this study, including the 95% UI, are accessible through the Global Health Data Exchange online query tool provided by the Institute for Health Metrics and Evaluation at https://ghdx.healthdata.org/. The GBD study used de-identified data, and informed consent waivers were approved by the University of Washington’s Institutional Review Board. All data summaries and analyses adhered to the Guidelines for Accurate and Transparent Reporting of Health Estimates (17).

### Risk factors

A review of the literature found that risk factors for AF/AFL were identified based on strong evidence of AF/AFL etiology, the availability of exposure data, and the potential for intervention (1, 18). The GBD 2021 data indicated that six key risk factors—high systolic blood pressure, high body-mass index, smoking, alcohol use, high-sodium diets, and lead exposure—were the most prominent factors influencing the burden of AF/AFL (2). Since GBD 2021 classifies metabolic, behavioral, and environmental factors as the first tier, each of the six dominant factors can be categorized into Tier 1 classifications (11). Therefore, we analyzed the burden of AF/AFL in different countries and regions with respect to the dominant risk factors of metabolism (high systolic blood pressure, high body-mass index), behavior (smoking, alcohol use, and high-sodium diets), and the environment (lead exposure), in order to implement prevention and adjustment strategies for AF/AFL. High systolic blood pressure was defined as a systolic blood pressure higher than 110–115 mmHg. High body-mass index was defined as a body-mass index higher than 20–25 kg/m^2^. Smoking was defined as the prevalence of current tobacco use and the prevalence of ever using any tobacco product. Alcohol use was defined as the average daily intake of pure alcohol (in grams per day) among current drinkers over the past 12 months. A high-sodium diet was defined as a 24-h average urinary sodium excretion (in grams per day) exceeding the theoretical minimum risk exposure level (TMREL) of 1–5 grams. Lead exposure was defined in two types: acute lead exposure, measured by blood lead levels (μg/dL), associated with decreased IQ in children, and chronic lead exposure, measured by bone lead levels (μg/g), linked to elevated systolic blood pressure and cardiovascular disease (19).

### Statistical analysis

#### Estimation of AF/AFL risk–attributable burden

This study follows the GBD2021 comparative risk assessment framework, comprising seven analytical steps. First, we assessed the strength of association between risk factors and AF/AFL using meta-regression, systematic review, and bias adjustment. In the second step, exposure levels and distributions of each risk factor were estimated using Bayesian statistical modeling techniques, including spatiotemporal Gaussian process regression and disease modeling meta-regression, with adjustments for error and bias. In the third step, TMREL was determined using a counterfactual scenario and the 85th percentile of exposure data from cohort studies and clinical trials. In the fourth step, population attribution scores were calculated for each risk-outcome pair, which quantifies the change in the proportion of healthy people whose exposure level would be to the TMREL. In the fifth step, exposure weights for each risk factor were calculated based on sex and age. In the sixth step, mediators were estimated to correct for the overestimation of population attribution scores resulting from potential interactions between risk factors. This step helps calculate the burden of disease attributable to combinations of multiple risk factors. Finally, the burden of AF/AFL attributable to specific risk factors is calculated by multiplying the population attribution score by the number of deaths or DALYs, stratified by age, sex, region, and year (11).

#### Temporal trend analysis of AF/AFL risk–attributable burden

In this study, we used Joinpoint trend analysis software (version 5.3.0) to analyze time trends in ASMR and ASDR for AF/AFL attributed to specific risk factors, stratified by age and gender at global, regional, and national levels. We applied the mean annual percentage change (AAPC) and its corresponding 95% confidence interval (CI) to quantify the long-term trends in the burden of AF/AFL attributable to each risk factor from 1990 to 2021. The AAPC was calculated as the weighted average of the slope coefficients in the Joinpoint regression model for the period from 1990 to 2021. If the AAPC is greater than 0 and the lower bound of the 95% CI is above 0 (*p* < 0.05), it indicates a significantly increasing trend in ASMR and ASDR from 1990 to 2021. Conversely, if the AAPC is less than 0 and the upper bound of the 95% CI is below 0 (*p* < 0.05), it indicates a significantly decreasing trend in ASMR and ASDR from 1990 to 2021. *p* < 0.05 represents statistical significance (20, 21).

## Results

### Global trends in AF/AFL risk–attributable burden

**Figure 1:**
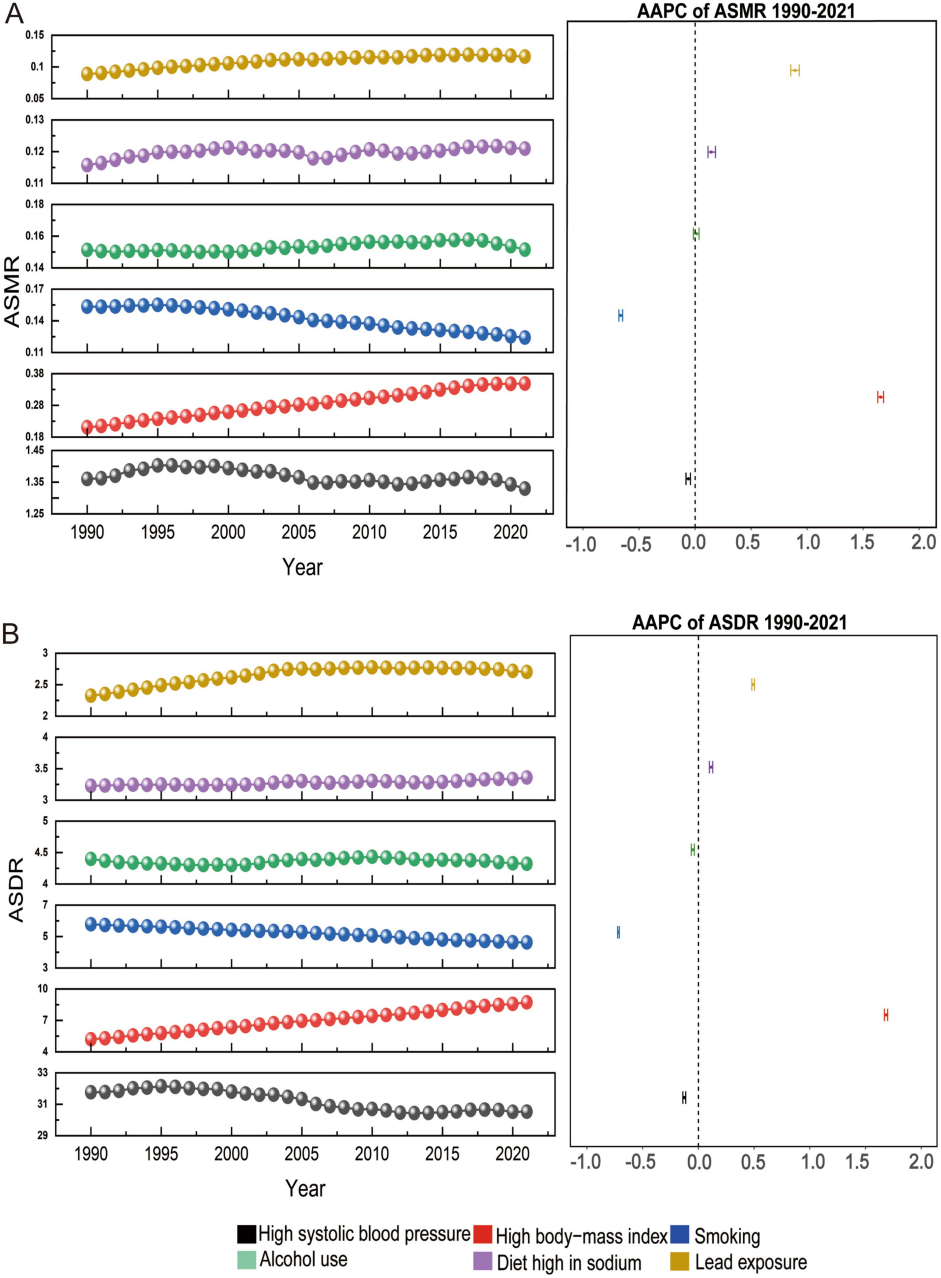
The trends and changes in atrial fibrillation **(A)** age-standardized mortality rate and **(B)** age-standardized disability-adjusted life year rate attributable to specific risk factors for both sexes combined globally from 1990 to 2021. AAPC: average annual percentage change; ASDR: age-standardized disability-adjusted life year rate; ASMR: age-standardized mortality rate.

As shown in Table 1, the global number of deaths and DALYs due to AF/AFL risk increased for both men and women between 1990 and 2021, while the corresponding ASMRs and ASDRs for certain risk factors declined. Deaths due to high systolic blood pressure increased from 36,854 (95% UI: 13,821–58,445) in 1990 to 103,423 (95% UI: 36,820–170,727) in 2021, while the corresponding ASMRs decreased from 1.36 (95% UI: 0.51–2.17) per 100,000 people in 1990 to 1.33 (95% UI: 0.47–2.20) per 100,000 people in 2021. DALYs and ASDRs due to high systolic blood pressure showed a similar trend over the same period. Smoking-related deaths increased from 4,769 (95% UI: 2,775–6,865) in 1990 to 10,012 (95% UI: 5,851–14,649) in 2021, while the corresponding ASMRs decreased from 0.15 (95% UI: 0.09–0.23) per 100,000 people in 1990 to 0.12 (95% UI: 0.07–0.18) per 100,000 people in 2021. DALYs and ASDRs due to smoking showed a similar trend over the same period. Furthermore, the increased burden of AF/AFL due to high systolic blood pressure and high body-mass index was predominantly observed in females, while the increased burden due to smoking and alcohol use was more prominent in males. AF/AFL deaths due to high-sodium diets and lead exposure were higher in females than in males, while DALY cases were more prevalent in males than in females (Supplementary Tables S1, S2). Figure 1 shows the trends in ASMR and ASDR for AF/AFL in both men and women combined, attributed to specific risk factors. Smoking, the leading risk factor for AF/AFL, has shown the most significant decline over the past 30 years, dropping from third in 1990 to fourth in 2021 (Supplementary Figure S1). The burden of AF/AFL due to high systolic blood pressure remains the highest (Supplementary Figure S1), although both ASMR and ASDR have decreased from 1990 to 2021. High body-mass index, the second-ranked risk factor (Supplementary Figure S1), has shown the most significant upward trend in AF/AFL burden from 1990 to 2021. Additionally, the burden of AF/AFL due to high-sodium diets and lead exposure also shows an increasing trend during the same period.

**Table 1:** Global burden of atrial fibrillation attributable to metabolic, behavioral and environmental factors for both sexes combined in 1990 and 2021.

Risk factor	Death (persons, 95%UI)^a^		ASMR^b^ (95%UI)		DALY^c^ (95%UI)		ASDR^d^ (95%UI)	
	1990	2021	1990	2021	1990	2021	1990	2021
High systolic blood pressure	36,854 (13,821–58,445)	103,423 (36,820–170,727)	1.36 (0.51–2.17)	1.33 (0.47–2.20)	1,049,327 (362,164–1,717,378)	2,514,582 (849,855–4,226,734)	31.77 (11.02–51.73)	30.53 (10.31–51.38)
High body-mass index	5,722 (2352–9,911)	27,237 (11,747–46,605)	0.21 (0.09–0.36)	0.35 (0.15–0.60)	175,032 (67,999–298,165)	724,574 (303,525–124,637)	5.19 (2.02–8.75)	8.71 (3.65–15.06)
Smoking	4,769 (2775–6,865)	10,012 (5,851–14,649)	0.15 (0.09–0.23)	0.12 (0.07–0.18)	218,722 (128,306–326,791)	396,210 (186,932–469,855)	5.78 (3.40–8.67)	4.62 (2.71–6.87)
Alcohol use	4,308 (3000–5,597)	11,908 (8,860–14,981)	0.15 (0.10–0.20)	0.15 (0.11–0.19)	155,704 (105,255–206,083)	362,698 (263,321–465,594)	4.40 (2.94–5.79)	4.32 (3.15–5.57)
Diet high in sodium	3,350 (385–10,222)	9,529 (781–30,753)	0.12 (0.01–0.36)	0.12 (0.01–0.39)	114,175 (15295–328,547)	282,457 (32,790–846,886)	3.23 (0.40–9.53)	3.36 (0.38–10.13)
Lead exposure	2,481(−357–6,298)	9,053 (−1,392–22,236)	0.09 (−0.01–0.22)	0.12 (−0.02–0.29)	79,858 (−10,305–205,458)	223,015 (−29,502–563,552)	2.32 (−0.30–5.95)	2.70 (−0.36–6.83)

a UI: uncertainty interval.

b ASMR: age-standardized mortality rate per 100,000 people.

c DALY: disability-adjusted life year of persons.

d ASDR: age-standardized disability-adjusted life year rate per 100,000 people.

### Global trends in AF/AFL risk–attributable burden by age groups

**Figure 2:**
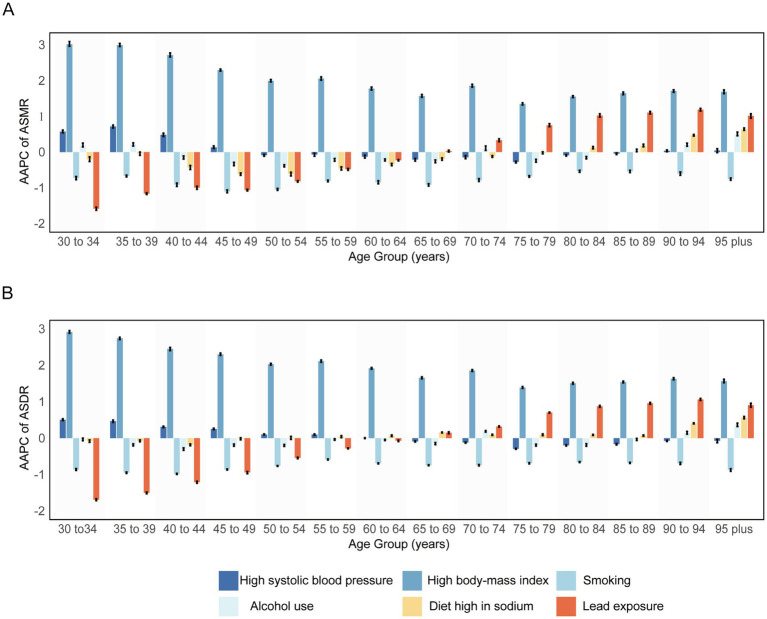
The average annual percentage change of **(A)** age-standardized mortality rate and **(B)** age-standardized disability-adjusted life year rate for both sexes combined by age groups in 1990-2021 globally. AAPC: average annual percentage change; ASDR: age-standardized disability-adjusted life year rate; ASMR: age-standardized mortality rate.

Figure 2 illustrates the effect of high systolic blood pressure on ASMR and ASDR across different age groups. Both ASMR and ASDR show an increasing trend in the 34–49 years age group, with the increase diminishing with age. After 65 years, both ASMR and ASDR reverse to a decreasing trend, with ASMR showing a slight increase after 90 years of age. Smoking-induced ASMR and ASDR showed a decreasing trend in all age groups, with a more significant decline in females than in males. The greatest decrease in females occurred in the 30–34 years age group (Supplementary Figures S2, S3). Similarly, ASMR and ASDR due to high body-mass index tended to increase in all age groups, with a larger increase in ASMR in males than females. The greatest increase in males occurred in the 30–34 years age group (Supplementary Figures S2, S3). It is noteworthy that the decline in ASMR and ASDR due to lead exposure diminishes with age, but after 65–69 years, the trend reverses and begins to increase. In females, the magnitude of the rise and fall due to lead exposure was more pronounced (Supplementary Figures S2, S3).

### Regional and SDI trends in AF/AFL risk–attributable burden

**Figure 3:**
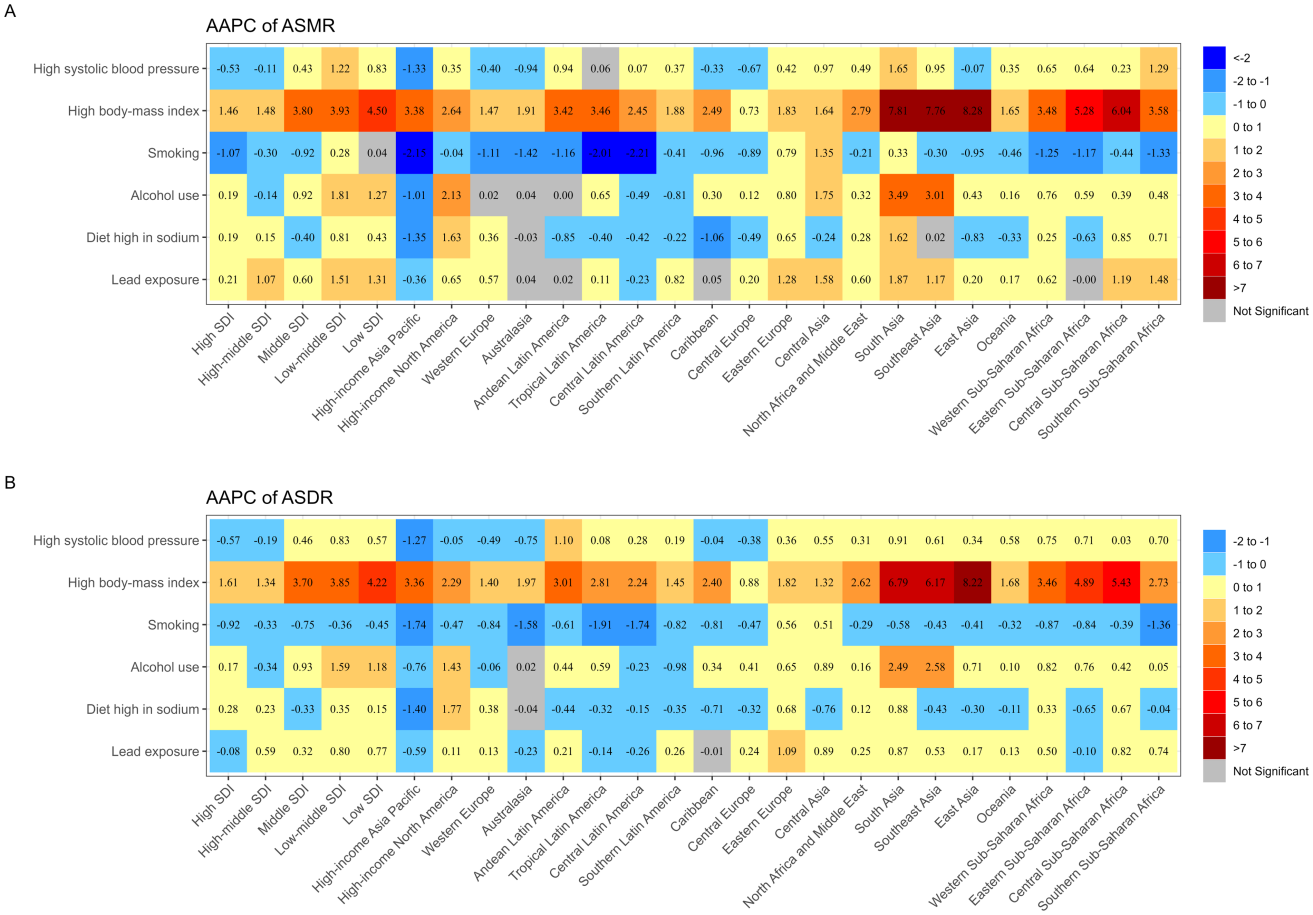
The average annual percentage change for atrial fibrillation risk–attributable **(A)** age-standardized mortality rate and **(B)** age-standardized disability-adjusted life year rate in 21 global burden of disease regions classified by 5 sociodemographic index levels for both sexes in 1990-2021. AAPC: average annual percentage change; ASDR: age-standardized disability-adjusted life year rate; ASMR: age-standardized mortality rate; SDI: sociodemographic index.

Figure 3 shows a significant upward trend in ASMR and ASDR related to high body fat across all regions, with East Asia showing the largest increase. Notably, in Eastern Europe, ASMR and ASDR for AF/AFL attributable to all risk factors have significantly increased. In High-income Asia Pacific, all risk factors for AF/AFL, except for high body-mass index, show a downward trend in ASMR and ASDR. High body-mass index and smoking are the primary risk factors driving the increase and decrease in AF/AFL burden in these regions. In the five SDI regions, the Low SDI region exhibits the largest increase in AF/AFL burden attributable to high body-mass index. As the SDI index increases, the upward trend persists but with a diminishing magnitude. Additionally, in the Low SDI and Low-middle SDI regions, attention should be paid to the effects of alcohol consumption, high systolic blood pressure, lead exposure, and high sodium diet on disease burden. Supplementary Figures S4, S5 displays the changes in ASMR and ASDR related to AF/AFL risk factors across 21 GBD regions and 5 SDI levels in males and females. Supplementary Table S3 in shows that Western Europe recorded the highest number of AF/AFL deaths associated with high systolic blood pressure in 2021, reaching 22,330 (95% UI: 7,887–37,015). East Asia, meanwhile, had the highest number of AF/AFL DALYs associated with high systolic blood pressure, reaching 512,597 (95% UI: 883,805–1,720,064). In terms of the SDI index, the High SDI region had the highest number of deaths in 2021, reaching 37,812 (95% UI: 13,104–63,082), as well as the highest number of DALYs, reaching 814,734 (95% UI: 266,923–1,379,270). It also had the highest ASMR and ASDR per 100,000 population. As the SDI index decreased, the number of deaths, DALYs, ASMR, and ASDR gradually declined across the regions.

### National trends in AF/AFL risk–attributable burden

**Figure 4:**
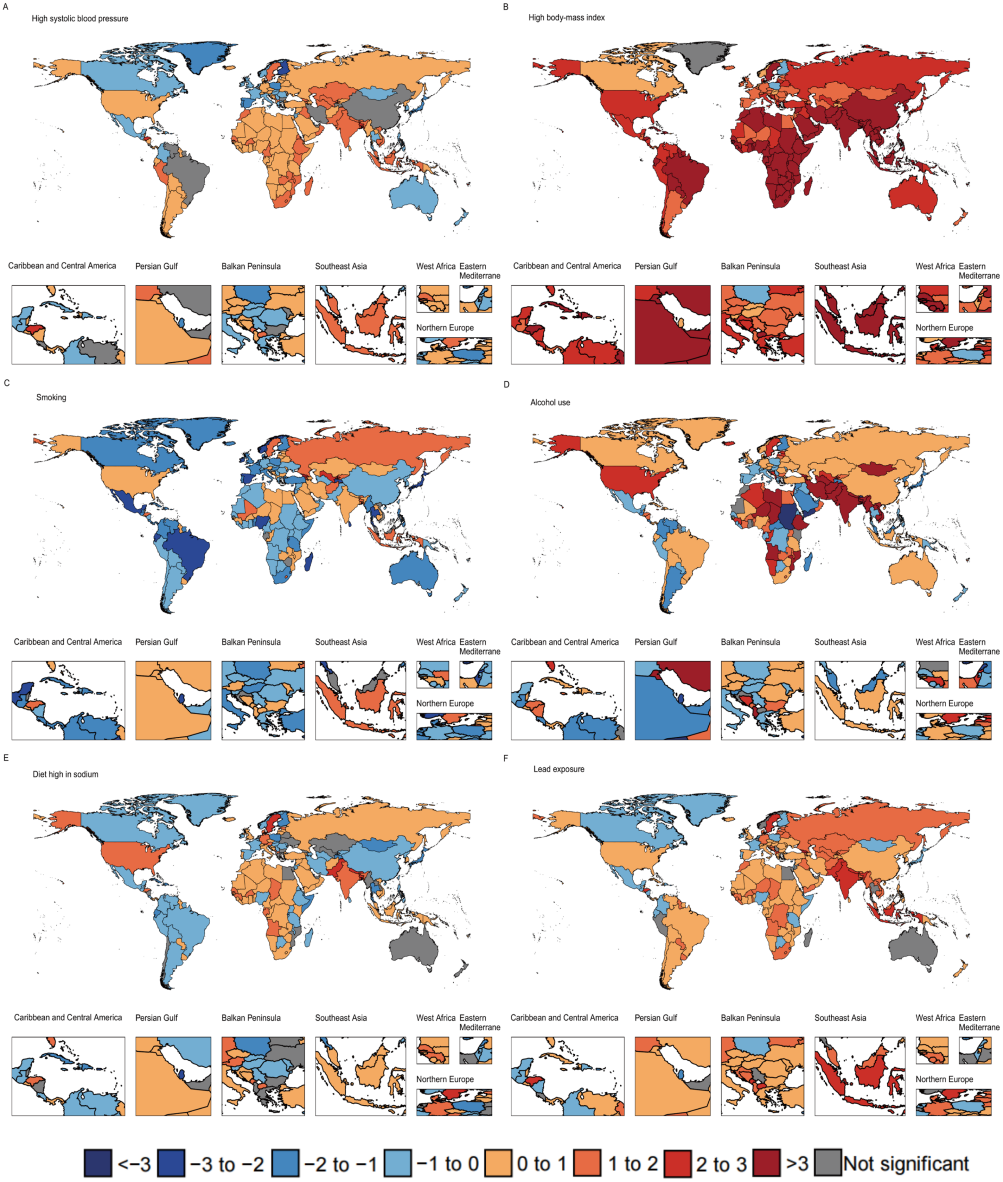
The average annual percentage change of atrial fibrillation/flutter age-standardized mortality rate attributable to high systolic blood pressure **(A)**, high body-mass index **(B)**, smoking **(C)**, alcohol use **(D)**, high-sodium diets **(E)**, and lead exposure **(F)** for both sexes combined in 204 countries and territories from 1990 to 2021.

Figure 4 presents the analysis of ASMR trends associated with risk factors for both sexes across 204 countries and territories. The results indicate that ASMR attributed to high body-mass index (198 countries, 97.1%), lead exposure (147 countries, 72.1%), high systolic blood pressure (144 countries, 70.6%), alcohol use (120 countries, 58.8%), and high-sodium diets (101 countries, 49.5%) increased significantly in the studied countries. In contrast, ASMR attributed to smoking decreased significantly in 140 countries (68.9%).

Although ASMR associated with high systolic blood pressure increased in most countries, the global trend declined overall. This may be attributed to smaller increases in countries with rising trends and larger decreases in a few countries, which significantly impacted the global trend. Specifically, Guam experienced the largest decrease in high systolic blood pressure-related ASMR (AAPC = −3.18, 95% CI: −3.58 to −2.82).

Between 1990 and 2021, the country with the largest increase in ASMR associated with high systolic blood pressure was Honduras (AAPC = 2.07, 95% CI: 1.94 to 2.18). The country with the largest increase in ASMR associated with high body-mass index was Bangladesh (AAPC = 16.42, 95% CI: 16.22 to 16.68) and the country with the largest decrease was Guam (AAPC = −2.26, 95% CI: −2.59 to −1.97). The country with the largest increase in ASMR associated with high-sodium diets was Bangladesh (AAPC = 2.89, 95% CI: 2.7 to 3.09), and the largest decrease was in Guam (AAPC = −4.46, 95% CI: −4.84 to −4.13). Iran had the largest increase in ASMR related to alcohol use (AAPC = 44.90, 95% CI: 43.27–46.4), while Sudan experienced the largest decrease (AAPC = −28.30, 95% CI: −29.67 to −26.86). Georgia showed the largest increase in ASMR related to lead exposure (AAPC = 3.04, 95% CI: 2.47–3.57), while Guam had the largest decrease (AAPC = −4.60, 95% CI: −4.98 to −4.25). The largest decrease in smoking-related ASMR occurred in San Marino (AAPC = −3.52, 95% CI: −4.02 to −3.27), whereas Georgia saw the largest increase (AAPC = 3.24, 95% CI: 2.75 to 3.74). Supplementary Figures S6, S7 in the show changes in ASMR trends related to risk attribution for males and females across 204 countries.

**Figure 5:**
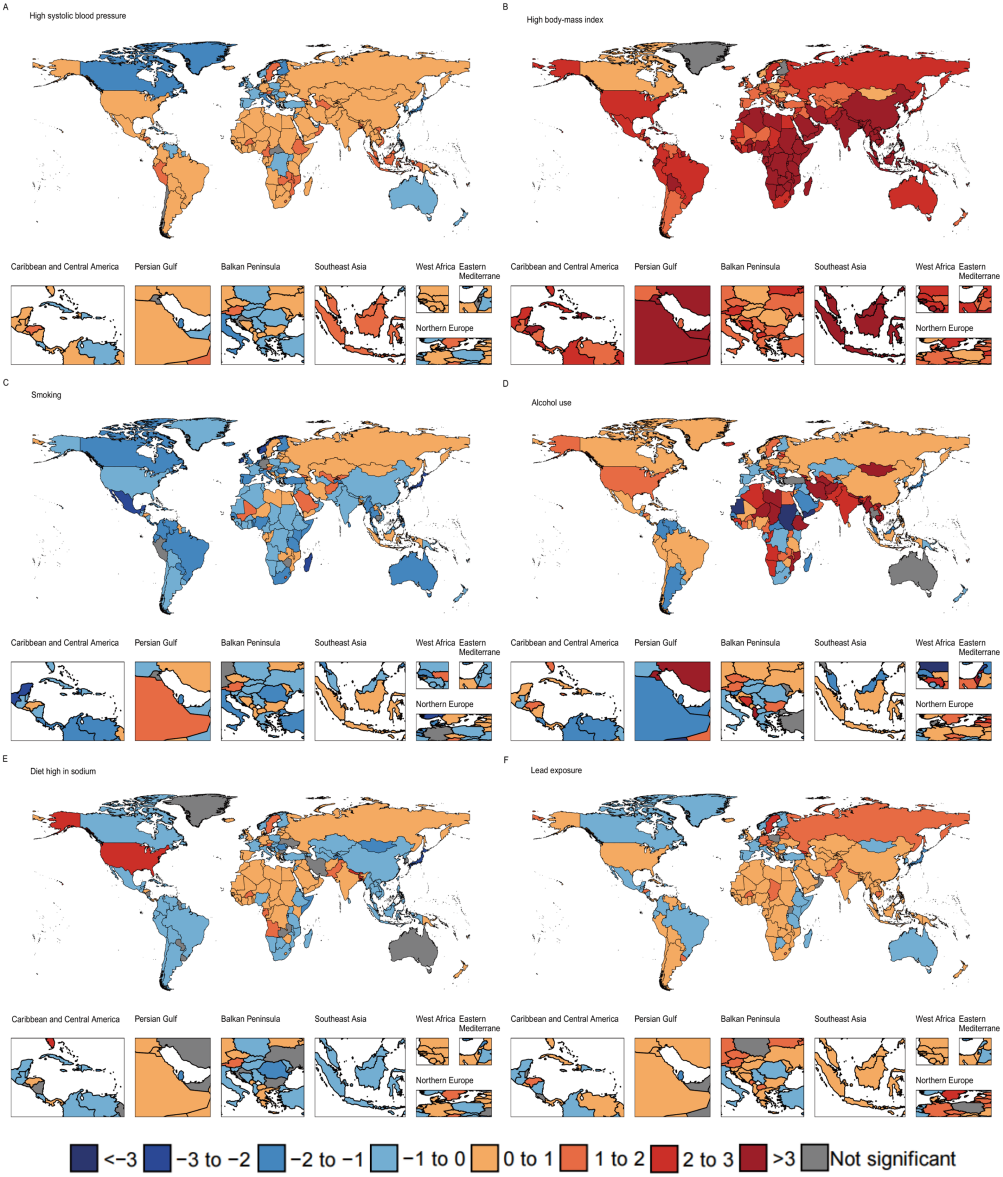
The average annual percentage change of atrial fibrillation/flutter age-standardized disability-adjusted life year rate attributable to high systolic blood pressure **(A)**, high body-mass index **(B)**, smoking **(C)**, alcohol use **(D)**, high-sodium diets **(E)**, and lead exposure **(F)** for both sexes combined in 204 countries and territories from 1990 to 2021.

Figure 5 presents the analysis of ASDR trends associated with risk factors for both sexes across 204 countries and territories. The results showed significant increases in ASDR due to high body-mass index (203 countries, 99.5%), high systolic blood pressure (146 countries, 71.6%), lead exposure (127 countries, 62.3%), alcohol use (120 countries, 58.8%), and high-sodium diets (85 countries, 41.7%) across the studied countries. In contrast, ASDR due to smoking decreased significantly in 150 countries (73.5%).

Between 1990 and 2021, the largest decrease in ASDR related to high systolic blood pressure was observed in Singapore (AAPC = −2.13, 95% CI: −2.14 to −2.11), while the largest increase occurred in Oman (AAPC = 1.80, 95% CI: 1.7 to 1.9). The largest increase in ASDR related to high body-mass index was observed in Viet Nam (AAPC = 21.04, 95% CI: 20.63 to 21.39). The largest increase in ASDR related to high-sodium diets occurred in Bhutan (AAPC = 2.11, 95% CI: 2.07 to 2.15), while the largest decrease occurred in Japan (AAPC = −1.80, 95% CI: −2.15 to −1.70). The largest increase in ASDR related to alcohol use was in Iran (AAPC = 42.73, 95% CI: 41.24 to 44.11), while the largest decrease occurred in Sudan (AAPC = −27.13, 95% CI: −28.31 to −25.93). The country with the largest increase in lead exposure-related ASDR was Georgia (AAPC = 2.19, 95% CI: 2.19 to 2.74), while the largest decrease was observed in Guam (AAPC = −2.02, 95% CI: −2.03 to −1.76). The country with the largest decrease in smoking-related ASDR was Ireland (AAPC = −2.61, 95% CI: −2.61 to −2.48), while the largest increase was observed in Georgia (AAPC = 1.63, 95% CI: 1.63 to 2.13).

According to Supplementary Table S4, in 2021, China, due to its large and aging population, was the most severely affected country, with the highest number of AF/AFL-related deaths and DALYs due to high systolic blood pressure, high-sodium diets, smoking, and lead exposure. The United States followed, with the highest number of AF/AFL deaths due to high body-mass index, as well as the highest DALYs. Supplementary Figures S8, S9 in the show changes in ASDR trends related to risk attribution for males and females across 204 countries.

## Discussion

### Principal findings

This study analyzed the geographic and temporal distribution of AF/AFL burden attributable to specific risk factors from 1990 to 2021 using the latest 2021 GBD data. The results show that, over the past 32 years, global deaths and DALYs from AF/AFL attributable to risk factors have increased for both men and women. However, the trends in the burden of AF/AFL attributable to risk factors have varied across different regions, countries, genders, and age groups. Notably, the effects of high body mass index and smoking on AF/AFL burden exhibited the most significant increasing and decreasing trends. Our findings offer key insights for regions and countries to implement targeted measures based on specific risk trends to reduce the AF/AFL burden.

Although the burden of AF/AFL associated with high systolic blood pressure has decreased over the past 30 years, it remains the leading cause of AF/AFL-related morbidity and mortality. In 2021, high systolic blood pressure was responsible for 103,423 deaths and 2,514,582 DALYs, accounting for approximately 30% of the global burden of AF/AFL. The trends in AF/AFL burden associated with high systolic blood pressure from 1990 to 2021 exhibit significant global variability, closely linked to levels of social development. The burden of AF/AFL related to high systolic blood pressure shifted from high and high−middle SDI regions to middle, low-middle, and low SDI regions, with a notable increase in the latter. This is partly due to limited awareness and resources for prevention, screening, and intervention, and partly because middle, low-middle, and low SDI regions show a stable or increasing burden of disease related to high systolic blood pressure (22–24). Without effective interventions, this growing burden will continue to worsen deaths and DALYs due to AF/AFL (25). Geographically, high systolic blood pressure has contributed to a significant increase in the burden of atrial fibrillation in the South Asia and Andean Latin America regions, highlighting the need for more effective hypertension control interventions in these areas. South Asia has made progress in reducing hypertension through home visits by trained government health workers, blood pressure monitoring, counseling services, physician training, and public sector care coordination (26). In contrast, the Andean Latin America region, where hypertension prevalence is elevated due to high altitude, is introducing new initiatives to control hypertension and other cardiovascular risks (27). The risk of cardiovascular diseases, including hypertension, is being addressed through the implementation of these new initiatives. Therefore, countries and regions must tailor global public health resources and strategies based on economic, geographic, and educational factors to effectively reduce the burden of AF/AFL by managing high systolic blood pressure.

High body-mass index is the second most important risk factor for the burden of AF/AFL, and there was a significant upward trend in AF/AFL under the influence of high body-mass index from 1990 to 2021, which is consistent with the increased incidence of high body-mass index reported around the world (28). Significant regional variations exist in the burden of AF/AFL associated with high body-mass index, closely linked to socioeconomic levels. While the burden of AF/AFL related to high body-mass index increased in all five SDI regions, the rise was more pronounced in low SDI regions compared to high SDI regions. Optimistically, obesity in high SDI regions is approaching its peak, with limited potential for further increase (28). In contrast, low SDI regions face a more pronounced burden of AF/AFL related to high body-mass index, driven by urbanization and lifestyle changes (29). Studies have shown that regions with higher socioeconomic status tend to consume healthier diets, rich in fruits and vegetables (30). Conversely, in underdeveloped areas with limited economic resources, people often rely on calorie-dense diets, favoring foods high in fat and sugar (31). Geographically, East Asia exhibits the most pronounced increase in the burden of atrial fibrillation related to high body-mass index. A pooled analysis of 20 prospective cohorts from the region revealed that high body-mass index significantly contributes to the rising burden of cardiovascular disease in East Asia (32). Analysis of 204 countries showed that Bangladesh and Vietnam had the highest increase in atrial fibrillation burden due to high body-mass index. These trends are linked to urbanization-driven dietary changes and the limited spread of fitness practices, resulting in greater weight gain and a rising burden of cardiovascular disease (33, 34).

From 1990 to 2021, the burden of smoking-related AF/AFL declined significantly, dropping from the third to the fourth leading risk factor in the ASMR rankings. The 2021 Global Burden of AF/AFL report indicated that smoking was the leading cause of AF/AFL deaths and ASMR in 2021, contributing 35 and 34.4%, respectively. Similarly, smoking was the top contributor to AF/AFL DALYs and ASDR, accounting for 43.9 and 43.3%, respectively (2). Geographically, trends in smoking-induced AF/AFL show significant variability, with notable declines primarily occurring outside Eastern Europe and Central Asia, The World Health Organization Framework Convention on Tobacco Control (FCTC) came into effect in 2005 (35). The FCTC aims to coordinate global efforts to combat the tobacco epidemic through measures such as marketing restrictions, tax increases, media campaigns promoting cessation, and smoke-free air laws (36). As highlighted in our study, these global successes in reducing smoking prevalence have contributed to reductions in ASMR and ASDR for smoking-related AF/AFL. In high-income countries, the decline in the burden of AF/AFL deaths related to smoking is the most pronounced, consistent with the global trend in smoking-related burden (37). Tobacco control measures have played a key role in the global reduction of smoking-related disease burden, with smoking prevalence decreasing by 27.5% in men and 37.7% in women (38). Therefore, reducing smoking prevalence is a critical strategy to alleviate the burden of atrial fibrillation.

The ASDR for alcohol use-related AF/AFL shows a gradual increase from 1990 to 2021, but remains relatively stable at the global level. Globally, alcohol use is responsible for approximately 12.7% of deaths and 1.8% of DALYs related to AF/AFL. Economic development is closely linked to alcohol consumption and the prevalence of drinkers. In many high SDI countries, early industrialization of alcohol production and a decrease in alcohol prices have led to an increase in both the number of drinkers and alcohol-related diseases (39). In middle- and low-income countries, industrialization has increased disposable income, enabling more people to purchase alcoholic beverages, which in turn has led to an increase in alcohol-related diseases (39, 40). Geographically, South Asia and Southeast Asia exhibit the most significant increase in the burden of AF/AFL related to alcohol consumption. This trend is influenced by various factors, including religion, family environment, economic development, and lifestyle habits (41). The Global Action Plan to Combat Non-communicable Diseases 2013–2020 is an integral component of the World Health Organization’s global strategy, adopted by the World Health Assembly in 2010. The plan prioritizes interventions aimed at reducing alcohol-related harm at the national level, focusing on cost-effective strategies. These strategies include increasing alcohol taxes, enhancing restrictions on alcohol access, and implementing comprehensive measures to restrict or ban alcohol advertising (42). During the period 2010–2017, the Philippines, Malaysia and Sri Lanka achieved effective alcohol control and reduced the burden of disease associated with alcohol consumption through a combination of specific taxes or the use of specific taxes alone (41).

Consistent with previous studies, high-sodium diet and lead exposure showed minimal variation, accounting for 35.5 and 31.7% of AF/AFL-related deaths, and 31.3 and 24.7% of AF-related disability adjustments, respectively. The global burden of AF/AFL attributable to these factors was significantly higher, though it remained stable or slightly decreased in some regions (2). A population-based epidemiological study suggests that a high-sodium diet may increase the risk of new AF/AFL events, as high-sodium intake contributes to AF/AFL by exacerbating inflammation and fibrosis in the atria (43). Another plausible explanation is that a high-sodium diet increases intracellular sodium levels and drives calcium into cells, which induces vascular smooth muscle contraction, raises peripheral vascular resistance, and subsequently leads to high systolic blood pressure, a major risk factor for AF/AFL (44). Studies have shown that the risk of AF/AFL is higher in cities with elevated pollution levels, where lead exposure exacerbates myocardial ischemia, increasing atrial pressure and triggering AF/AFL (45). The most significant reductions in the AF/AFL burden related to high sodium diets and lead exposure were observed in the High-income Asia Pacific region. This can be attributed to higher income levels, greater environmental awareness, better access to healthier low-nutrient foods, and improved air governance in high-income areas. In contrast, rising AF/AFL burdens associated with high sodium diets were seen in North America, while increased AF/AFL burdens related to lead exposure were observed in South Asia and Eastern Europe. Given these variations, policymakers should design interventions tailored to local risk factors to promote healthier diets and environmental governance.

### Risk exposure of AF/AFL burden among sexes and age groups

In the geographic and temporal distribution of risk exposure to the AF/AFL burden, significant differences exist across regions, genders, and age groups. High systolic blood pressure and high body-mass index contribute to a greater burden of AF/AFL in women than in men, as reflected in the number of deaths and DALYs. This suggests a gender disparity in AF/AFL associated with high systolic blood pressure and high body-mass index. Although men are more likely than women to have high systolic blood pressure, men also have a higher prevalence of high body-mass index due to poor eating habits and lower Healthy Eating Index scores, both of which increase the risk of AF/AFL (46). However, women may be more susceptible to adverse effects, such as torsades de pointes and bleeding, when using antiarrhythmic drugs. Additionally, women are less likely than men to receive invasive rhythm control therapy (47–49). Thus, women face a higher risk of AF/AFL-related death from risk factors than men and are more prone to complications. In contrast, smoking and alcohol consumption rates are much higher in men than in women, resulting in a greater AF/AFL burden in men (50). The increasing trend in ASMR from AF/AFL due to high systolic blood pressure was particularly significant among individuals aged 30–49 years, indicating a progressive shift towards younger populations in the prevalence of hypertension. A survey revealed that the number of cardiovascular disease deaths associated with high systolic blood pressure in young people worldwide increased by 43.0% in 2019 compared to 1990 (51). At the same time, a trend of decreasing AF/AFL DALYs caused by high systolic blood pressure in the elderly was observed, suggesting that the growing societal focus on hypertension in older adults, coupled with the maturation of the global healthcare system for the elderly, has led to improved prevention and control of hypertension-induced AF/AFL in this group. Temporal trends in the burden of AF/AFL associated with high body mass index and smoking exhibited significant increases and decreases across sexes and age groups, with notable regional variation. This may be attributed to regional differences in the rates of high body mass index and smoking. The greatest reduction in the burden of AF/AFL due to smoking occurred in women aged 30–34 years, and this downward trend reflects the effectiveness of global tobacco control policies (35). Studies also suggest that women under 35 attempt to quit smoking more frequently, likely due to easier access to nicotine gums and patches (52). Additionally, research shows that those who quit before 35 have a mortality rate similar to that of never-smokers (53). As a result, public health agencies have set this age as a key target for encouraging young smokers to quit. As they approach 35, smokers may become more aware of the health risks of smoking, further strengthening their motivation to quit (54). In contrast, the greatest increase in AF/AFL burden due to high body mass index was seen in men aged 30–34. Future interventions should therefore focus on men who continue smoking beyond 35 and those aged 30 to 34 with a high body mass index. Additionally, temporal trends in the burden of AF/AFL due to alcohol use differ between men and women. For example, in the 30–39 years age group, men show an increasing trend, while women exhibit a decreasing trend. A previous study found that 3.8% of deaths in women and 12.2% of deaths in men aged 15–49 years were attributable to alcohol use, with the burden being approximately three times higher in men than in women (50). The burden of AF/AFL from high-sodium diets and lead exposure followed similar age and gender trends, shifting from a decline to an increase with age. Notably, the highest rise in AF/AFL due to high-sodium diets occurred in women aged 95 and older, while lead exposure had the greatest impact on those aged 90–94. This age-related pattern in lead exposure aligns with broader environmental and physiological factors. Stringent environmental regulations have driven the phase-out of lead-containing products and increased lead waste recycling, leading to significantly lower blood lead levels in younger generations, approaching pre-industrialization levels (55, 56). However, older adults tend to accumulate more lead over their lifetime, and postmenopausal women, in particular, are more susceptible to bone loss. This process releases stored lead from bones into the bloodstream, causing blood lead levels to rise with age and subsequently increasing the risk of atrial AF/AFL and other cardiovascular conditions (57, 58). Given these findings, older women should consider adopting a low-sodium diet and be more mindful of the cardiovascular effects of air pollution. Addressing gender- and age-related disparities in AF/AFL burden requires targeted policies that not only promote healthier lifestyles but also foster international collaboration to mitigate the global impact of AF/AFL.

### Public health implications

By analyzing changes in major risk factors affecting AF/AFL across countries, this study provides policymakers and decision-makers with insights to guide public health resource allocation. Given limited resources, interventions targeting geographic variations and high-risk populations can effectively reduce the burden of AF/AFL and mitigate the socioeconomic costs of the disease. We emphasize prioritizing the AF/AFL burden based on specific risk factor attributions, aligning strategies across countries, including metabolic (e.g., blood pressure control, weight management), behavioral (e.g., tobacco and alcohol policy development, low-sodium diets), and environmental (e.g., air pollution control) interventions. Given the increased burden of AF/AFL associated with high body mass index in most countries, we recommend that public health policymakers and decision-makers prioritize interventions to curb the rise of AF/AFL through the design and implementation of effective policies. There is an urgent need to educate populations about risk factor prevention in countries with high AF/AFL burdens. Additionally, countries should improve data sharing and promote global disease surveillance and early warning systems, providing a stronger foundation for an effective response to the AF/AFL burden.

### Strengths and limitations

This study provides a comprehensive temporal and spatial analysis of the AF/AFL burden associated with specific risk factors, providing targeted insights for developing and adapting effective strategies. By focusing on key risk factors, including metabolism, behavior, and environment, the study assessed the temporal trends in AF/AFL burden. Based on these findings, future studies should stratify management by country, region, gender, and age to proactively address rising-risk factors. We anticipate that some limitations of the study will be addressed in future research: First, AF/AFL encompasses several subtypes, including paroxysmal, persistent, and permanent AF/AFL, each potentially having distinct risk factors, trends, and geographic patterns. However, specific stratification data for these subtypes are not available in the GBD. Second, data for certain countries and regions may be derived from models based on limited samples, which may not fully represent the entire region or country. Third, GBD 2021 newly introduced BPRF method accounts for interstudy heterogeneity and potential systematic biases, resulting in wider 95% UI. This broader UI may increase uncertainty in risk-outcome associations and potentially underestimate or overestimate true risks. Finally, we did not analyze the temporal and geographic variation in AF/AFL burden due to other risk factors, such as obstructive sleep apnea and comorbidities, because data on these variables were unavailable in the GBD database. This limitation may have led to an overestimation of the contribution of the risk factors included in this study to the AF/AFL burden.

## Conclusion

This study, using GBD2021 data, conducted a multi-level, longitudinal analysis of the AF/AFL attributable risk burden. The results show that over the past 32 years, the number of deaths and DALYs attributable to AF/AFL have significantly increased, but the trends of different risk factors vary under different stratifications. Globally, the burden of AF/AFL from high body-mass index, high sodium diet, and lead exposure has increased, while the burden from smoking and high systolic blood pressure has decreased, and alcohol use has remained stable. Among the five SDI regions, low-income areas have experienced the most significant increase in AF/AFL burden due to high body-mass index. Geographically, East Asia has shown the largest increase in AF/AFL burden due to high body-mass index, while Iran is the country with the largest increase in AF/AFL burden due to alcohol use. Age- and sex-stratified analysis revealed that AF/AFL burden from high systolic blood pressure and body-mass index has significantly increased in younger men and women respectively, while burden from high sodium diet and lead exposure has notably risen in elderly women. This study provides multi-dimensional evidence to support the development of effective intervention strategies and helps reduce the burden of AF/AFL.

## Data Availability

The datasets presented in this study can be found in online repositories. The names of the repository/repositories and accession number(s) can be found in the article/Supplementary material.
