# Supplementary material for: Global, regional, and national burden of atrial fibrillation/flutter attributable to metabolic, behavioral, and environmental risk factors, 1990–2021: a longitudinal observational study
Source: Front Nutr. 2025 May 15;12:1560334. doi: 10.3389/fnut.2025.1560334 (PMC12107444; doi:10.3389/fnut.2025.1560334)
Supplement: Supplementary file 1 [file Table_1.docx]

**Supplementary Information and Data**

Global, Regional, and National Burden of Atrial Fibrillation/Flutter Attributable to Metabolic, Behavioral, and Environmental Risk Factors, 1990–2021: A Longitudinal Observational Study

**Table of content**

Table S1. Global burden of atrial fibrillation attributable to metabolic, behavioral and environmental factors for males in 1990 and 2021

Table S2. Global burden of atrial fibrillation attributable to metabolic, behavioral and environmental factors for females in 1990 and 2021

Figure S1: Changes in the ranking of both sexes with atrial fibrillation/flutter (A) age-standardized mortality rates and (B) age-standardized disability-adjusted life-year rates attributable to specific risk factors, globally, 1990-2021. Dashed lines indicate decrease in rank. Solid lines indicate increase or no change in rank. Data in parentheses are 95% CIs.

Figure S2: The AAPCs of ASMR (A) and ASDR (B) for males at age groups in 1990-2021, globally.

Figure S3: The AAPCs of ASMR (A) and ASDR (B) for females at age groups in 1990-2021, globally.

Figure S4: The AAPC for AF/AFL risk attributable ASMR(A) and ASDR(B) for males in 21 GBD regions and 5 SDI levels, 1990-2021.

Figure S5: The AAPC for AF/AFL risk attributable ASMR(A) and ASDR(B) for females in 21 GBD regions and 5 SDI levels, 1990-2021.

Figure S6: The AAPC of AF/AFL ASMR attributable to specific risk factors for males at 204 countries and territories from 1990 to 2021.

Figure S7: The AAPC of AF/AFL ASMR attributable to specific risk factors for females at 204 countries and territories from 1990 to 2021.

Figure S8:The AAPC of AF/AFL ASDR attributable to specific risk factors for males at 204 countries and territories from 1990 to 2021.

Figure S9: The AAPC of AF/AFL ASDR attributable to specific risk factors for females at 204 countries and territories from 1990 to 2021.

Table S3. The burden of AF/AFL attributable to metabolic, behavioral, and environmental risk factors in 21 GBD regions and 5 SDI levels in 1990 and 2021.

Table S4. The burden of AF/AFL attributable to metabolic,behavioral,and environmental risk factors for both sexes combined in 204 countries and territories in 2021.

**Table S1. Global burden of atrial fibrillation attributable to metabolic, behavioral and environmental factors for males in 1990 and 2021**

| **Risk factor** | **Death(persons,95%UI^a^)** | | **ASMR^b^(95%UI)** | | **DALY^c^(95%UI)** | | **ASDR^d^(95%UI)** | |
| --- | --- | --- | --- | --- | --- | --- | --- | --- |
|  | **1990** | **2021** | **1990** | **2021** | **1990** | **2021** | **1990** | **2021** |
| **High systolic blood pressure** | 36854(13821-58445) | 103423(36820-170727) | 1.36(0.51-2.17) | 1.33(0.47-2.20) | 1049327(362164-1717378) | 2514582(849855-4226734) | 31.77(11.02-51.73) | 30.53(10.31-51.38) |
| **High body-mass index** | 5722(2352-9911) | 27237(11747-46605) | 0.21(0.09-0.36) | 0.35(0.15-0.60) | 175032(67999-298165) | 724574(303525-124637) | 5.19(2.02-8.75) | 8.71(3.65-15.06) |
| **Smoking** | 4769(2775-6865) | 10012(5851-14649) | 0.15(0.09-0.23) | 0.12(0.07-0.18) | 218722(128306-326791) | 396210(186932-469855) | 5.78(3.40-8.67) | 4.62(2.71-6.87) |
| **Alcohol use** | 4308(3000-5597) | 11908(8860-14981) | 0.15(0.10-0.20) | 0.15(0.11-0.19) | 155704(105255-206083) | 362698(263321-465594) | 4.40(2.94-5.79) | 4.32(3.15-5.57) |
| **Diet high in sodium** | 3350(385-10222) | 9529(781-30753) | 0.12(0.01-0.36) | 0.12(0.01-0.39) | 114175(15295-328547) | 282457(32790-846886) | 3.23(0.40-9.53) | 3.36(0.38-10.13) |
| **Lead exposure** | 2481(-357-6298) | 9053(-1392-22236) | 0.09(-0.01-0.22) | 0.12(-0.02-0.29) | 79858(-10305-205458) | 223015(-29502-563552) | 2.32(-0.30-5.95) | 2.70(-0.36-6.83) |

^a^UI:uncertainty interval.

^b^ASMR: age-standardized mortality rate per 100,000 people.

^c^DALY: disability-adjusted life year of persons.

^d^ASDR: age-standardized disability-adjusted life year rate per 100,000 people.

**Table S2. Global burden of atrial fibrillation attributable to metabolic, behavioral and environmental factors for females in 1990 and 2021**

| **Risk factor** | **Death(persons,95%UI^a^)** | | **ASMR^b^(95%UI)** | | **DALY^c^(95%UI)** | | **ASDR^d^(95%UI)** | |
| --- | --- | --- | --- | --- | --- | --- | --- | --- |
|  | **1990** | **2021** | **1990** | **2021** | **1990** | **2021** | **1990** | **2021** |
| **High systolic blood pressure** | 23699(9086-37551) | 64032(22374-106403) | 1.39(0.53-2.21) | 1.35(0.47-2.24) | 575205(203721-929977) | 1339240(448977-2216431) | 30.07(10.67-48.70) | 28.51(9.56-47.21) |
| **High body-mass index** | 4194(1752-7181) | 18059(7717-30760) | 0.25(0.01-0.41) | 0.38(0.16-0.65) | 111651(44456-189380) | 418636(44456-189380) | 5.75(2.29-9.72) | 8.93(3.77-15.30) |
| **Smoking** | 1466(853-2172) | 2708(1520-4079) | 0.08(0.05-0.12) | 0.06(0.03-0.07) | 48476(27862-73360) | 77712(232054-586952) | 2.40(1.37-3.63) | 1.67(0.95-2.55) |
| **Alcohol use** | 1593(1039-2122) | 3636(2508-4840) | 0.09(0.06-0.13) | 0.08(0.05-0.1) | 41309(27543-55327) | 77941(55350-101884) | 2.14(1.41-2.86) | 1.66(1.18-2.17) |
| **Diet high in sodium** | 1786(166-5725) | 4784(282-16600) | 0.10(0.009-0.33) | 0.10(0.06-0.35) | 50436(5525-157801) | 115951(10395-362281) | 2.54(0.27-8.03) | 2.48(0.22-7.73) |
| **Lead exposure** | 1331(-201-3502) | 4846(-767-12331) | 0.08(-0.01-0.20) | 0.10(0.02-0.26) | 35915(-4858-94304) | 103189(-14031-266078) | 1.84(-0.25-4.83) | 2.20(-0.30-5.67) |


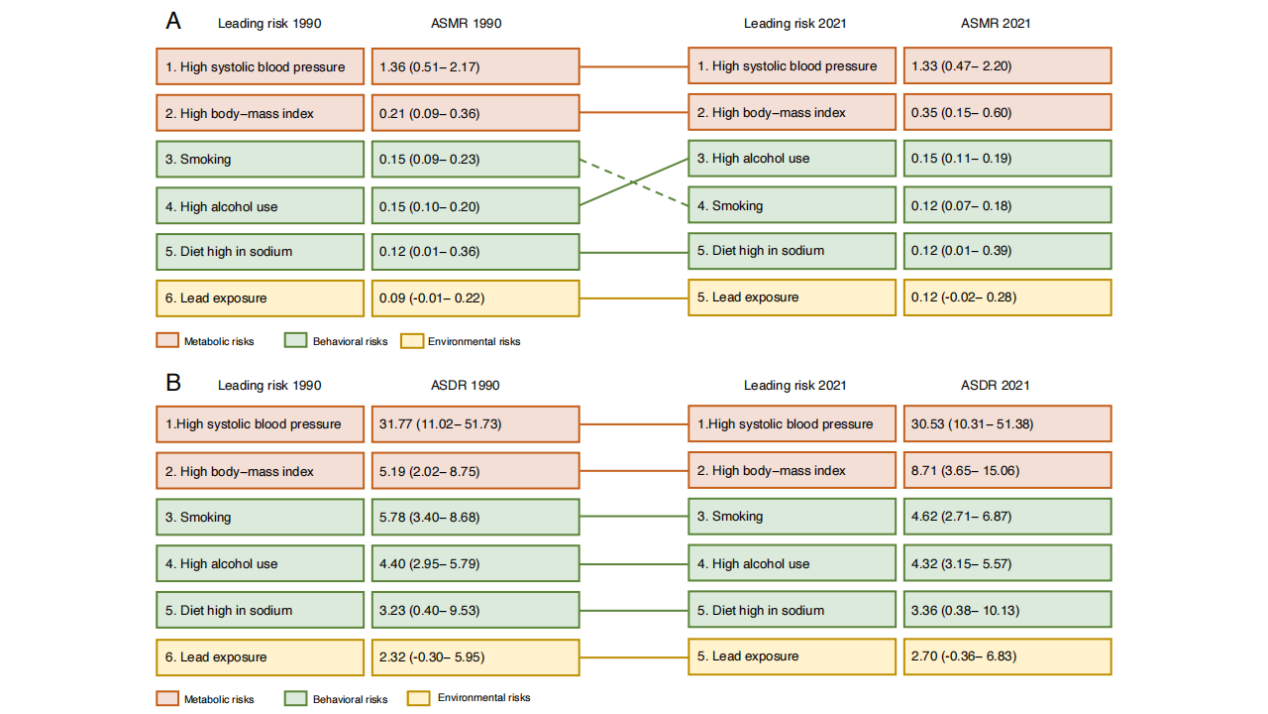
Figure S1: Changes in the ranking of both sexes with atrial fibrillation/flutter (A) age-standardized mortality rates and (B) age-standardized disability-adjusted life-year rates attributable to specific risk factors, globally, 1990-2021. Dashed lines indicate decrease in rank. Solid lines indicate increase or no change in rank. Data in parentheses are 95% CIs.


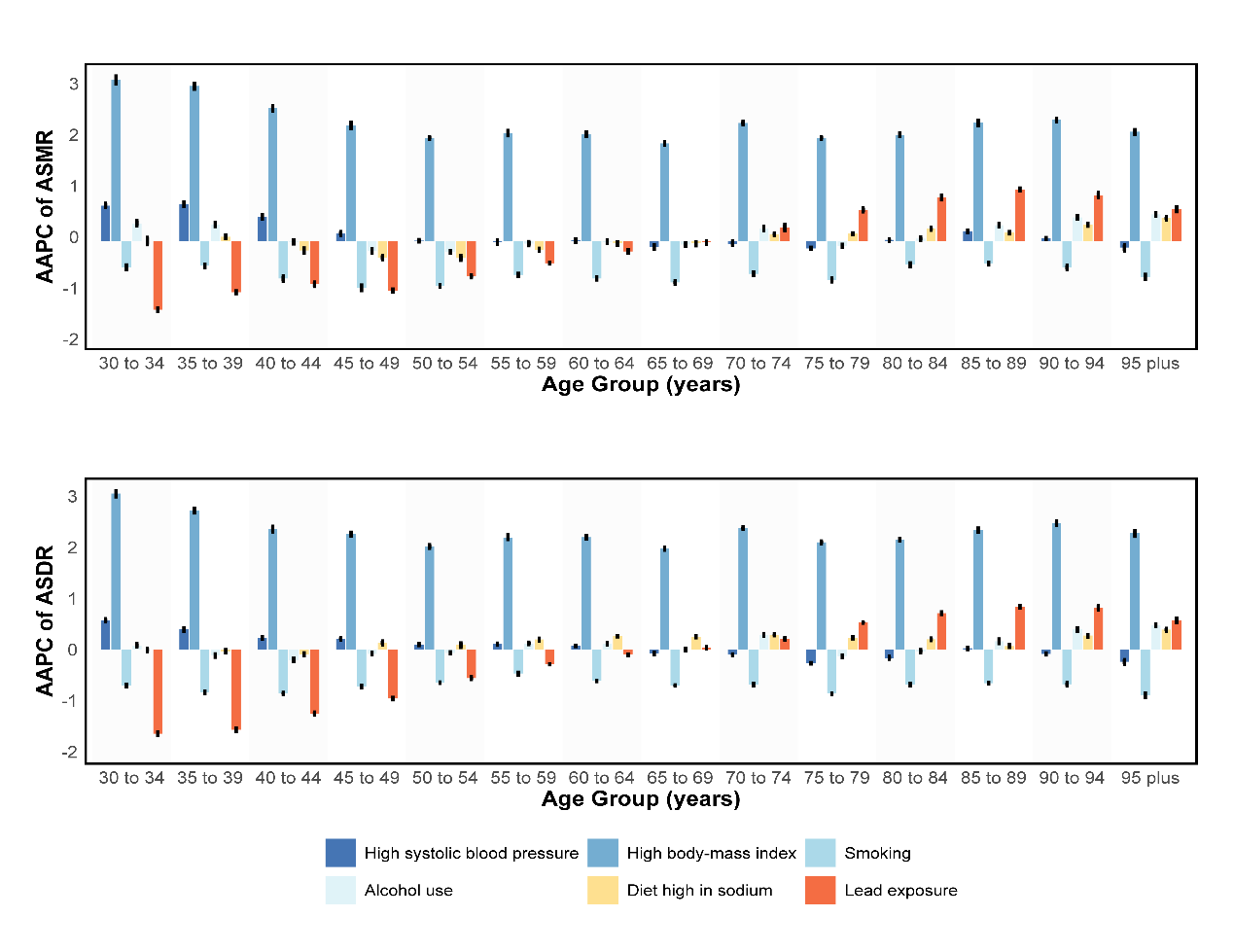


Figure S2: The AAPCs of ASMR (A) and ASDR (B) for males at age groups in 1990-2021, globally.


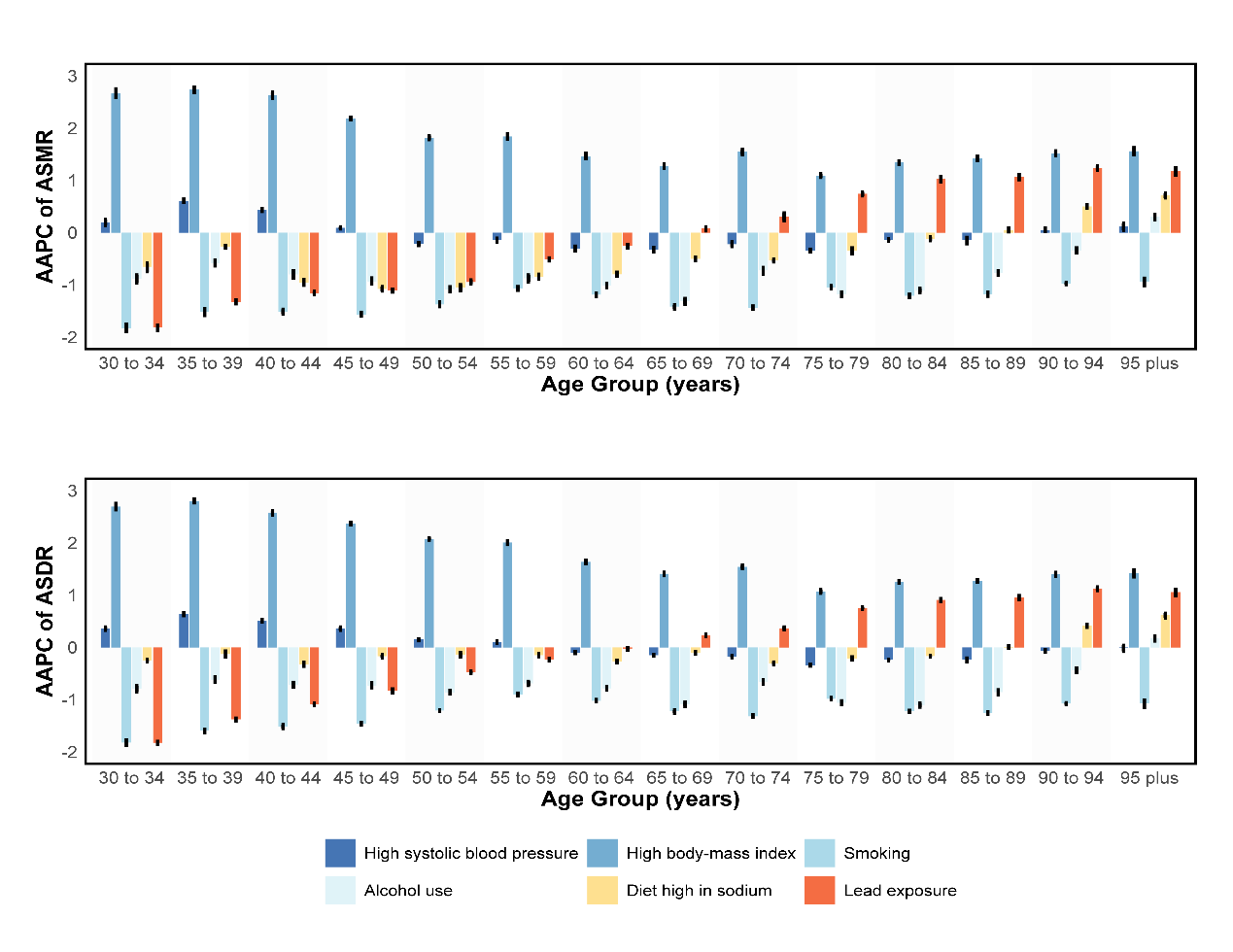


Figure S3: The AAPCs of ASMR (A) and ASDR (B) for females at age groups in 1990-2021, globally.


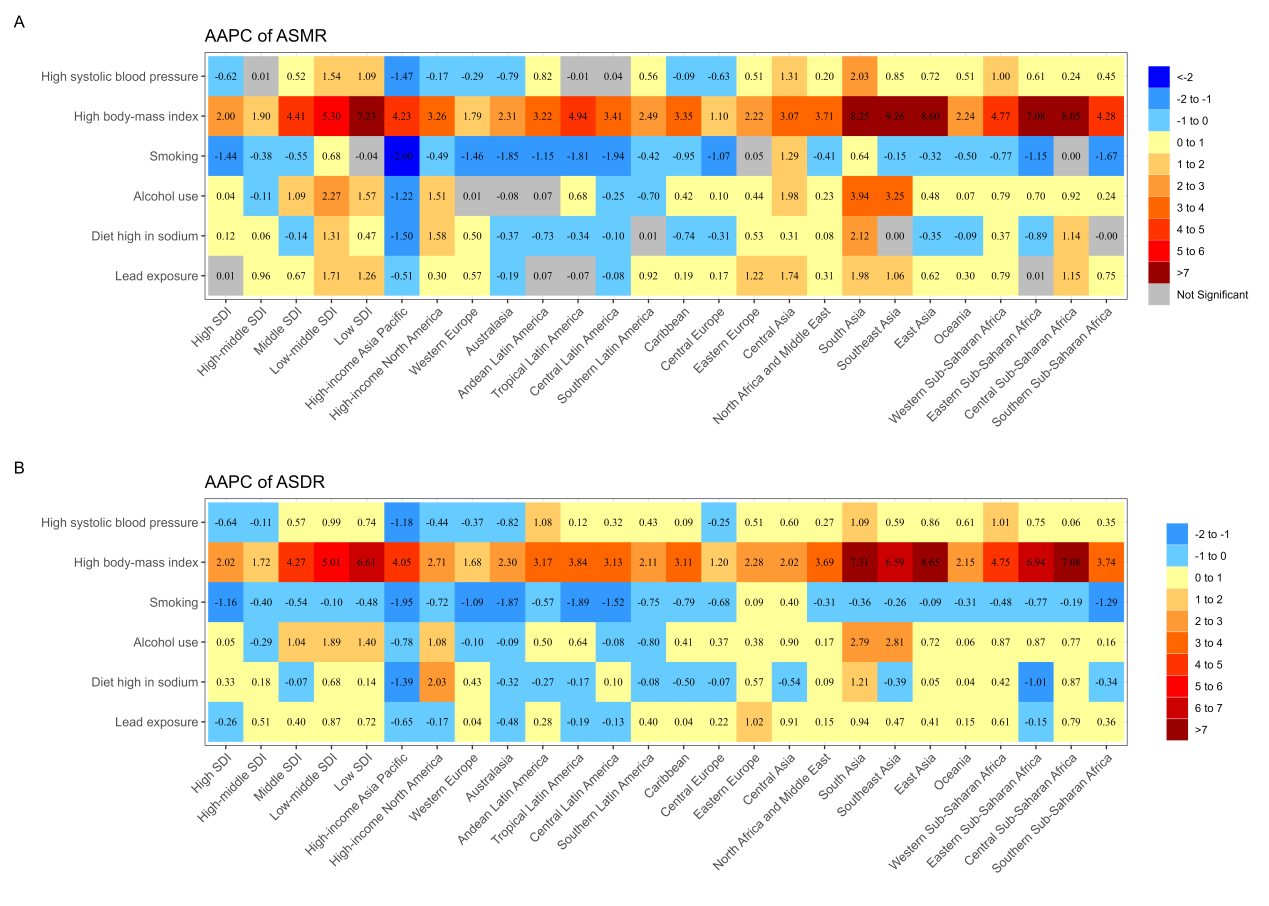


Figure S4: The AAPC for AF/AFL risk attributable ASMR(A) and ASDR(B) for males in 21 GBD regions and 5 SDI levels, 1990-2021.


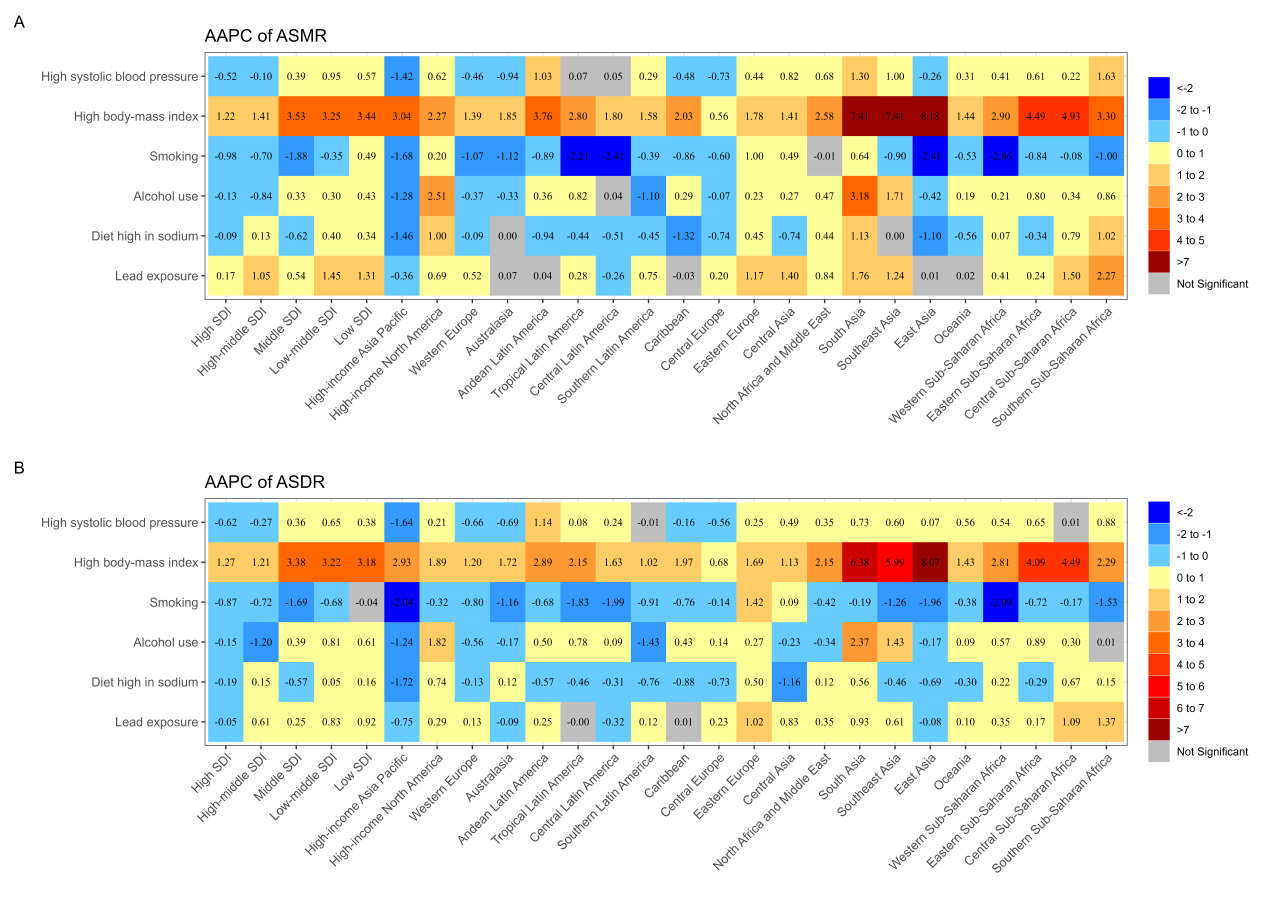


Figure S5: The AAPC for AF/AFL risk attributable ASMR(A) and ASDR(B) for females in 21 GBD regions and 5 SDI levels, 1990-2021.


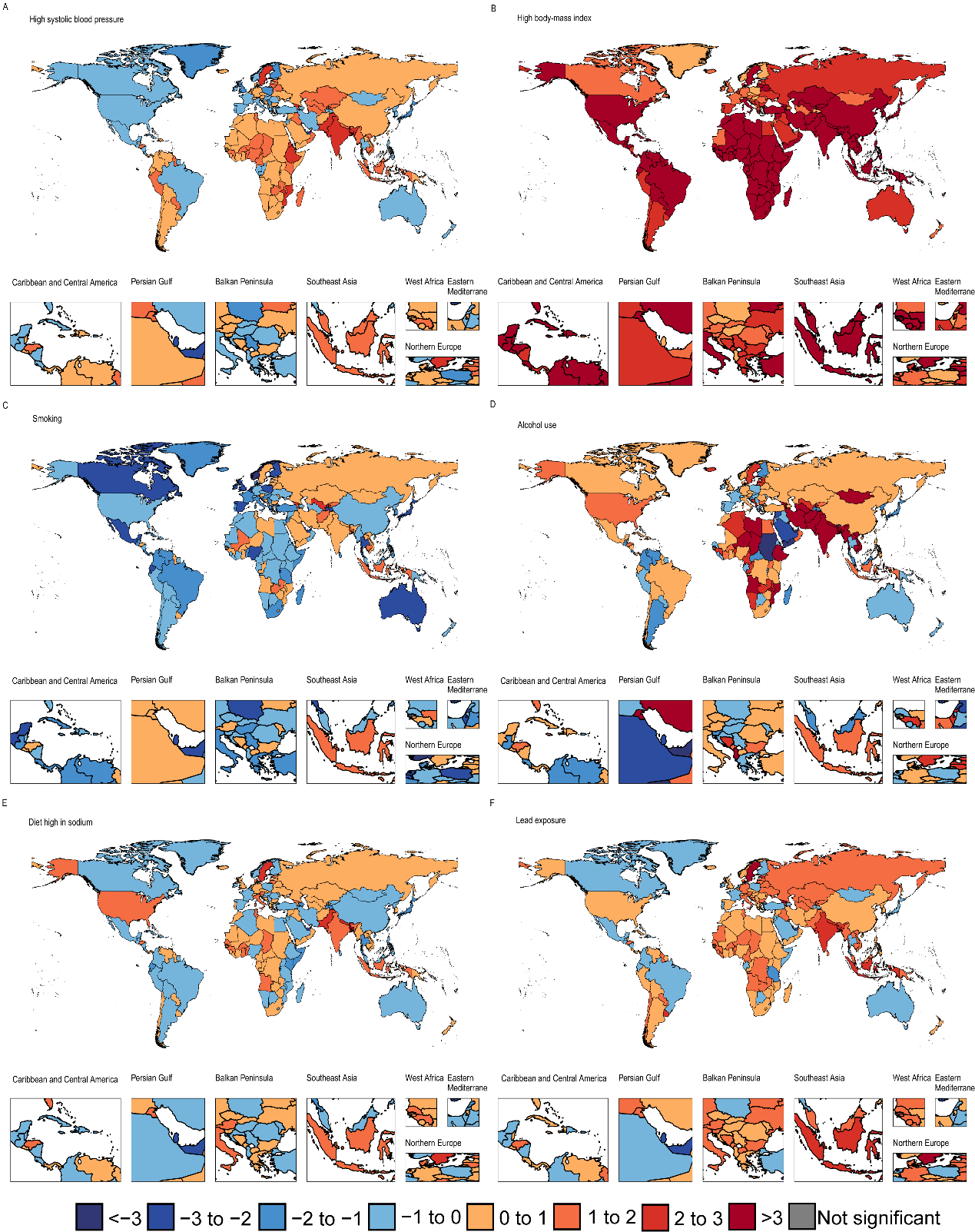

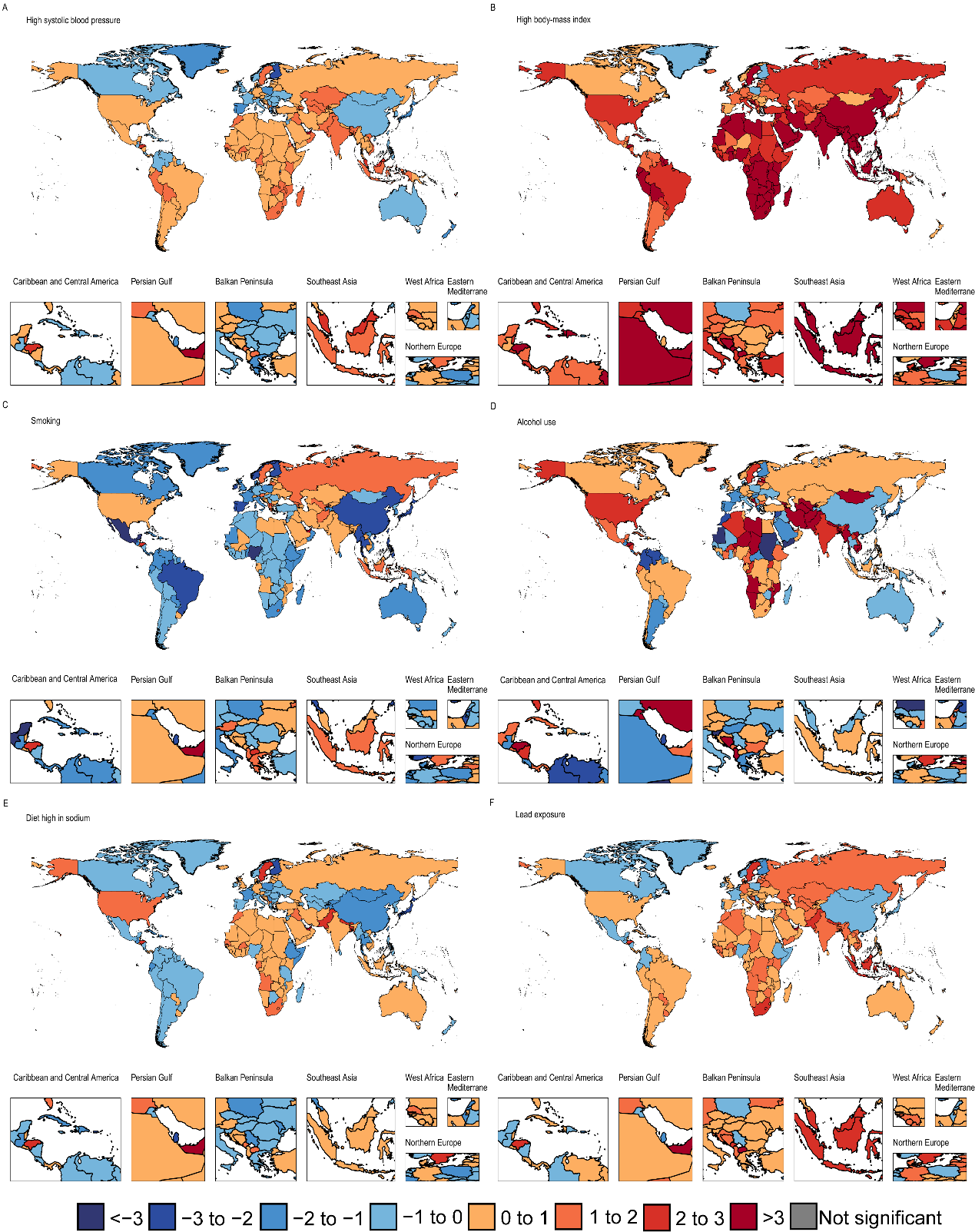


Figure S7: The AAPC of AF/AFL ASMR attributable to specific risk factors for females at 204 countries and territories from 1990 to 2021.


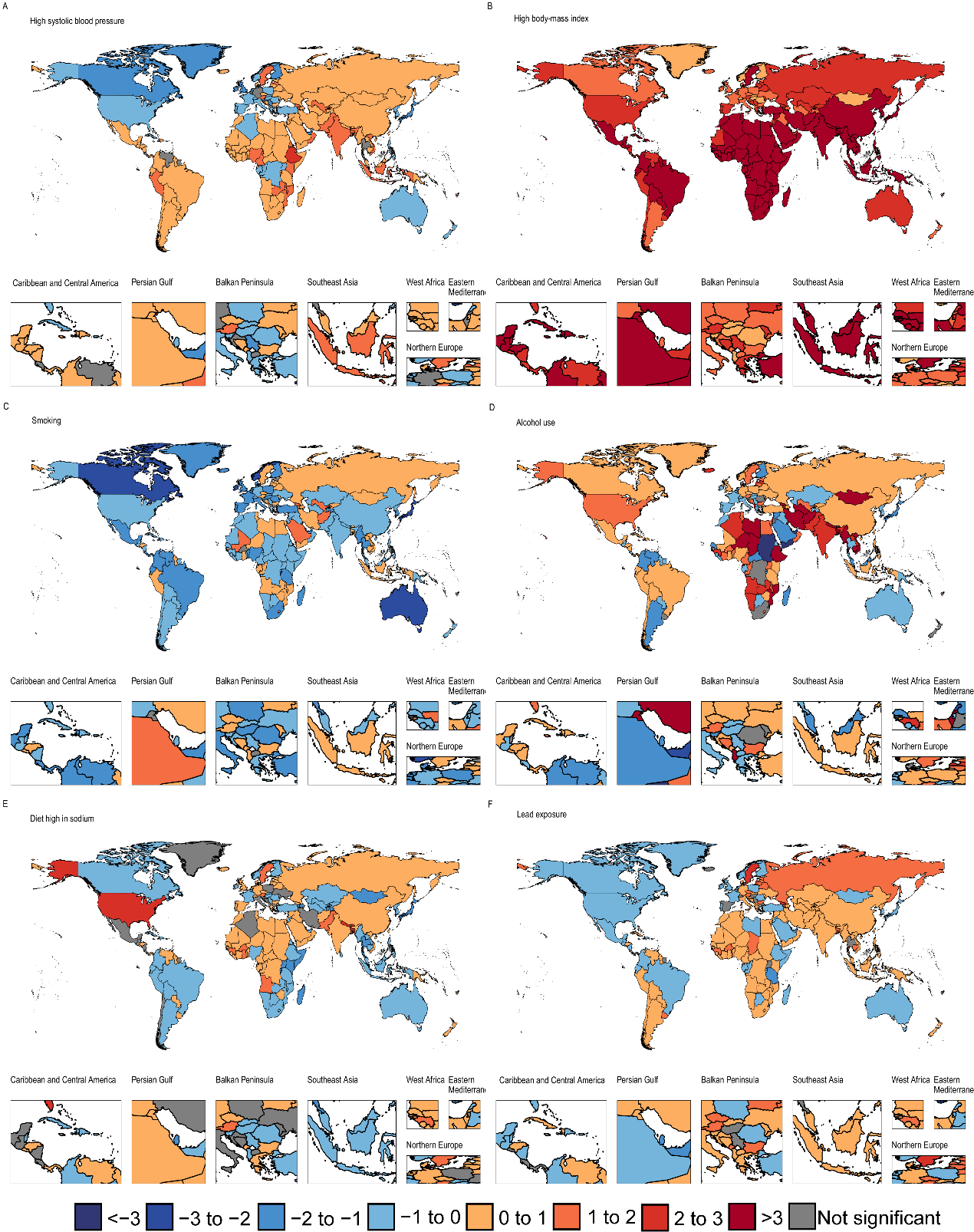


Figure S8:The AAPC of AF/AFL ASDR attributable to specific risk factors for males at 204 countries and territories from 1990 to 2021.


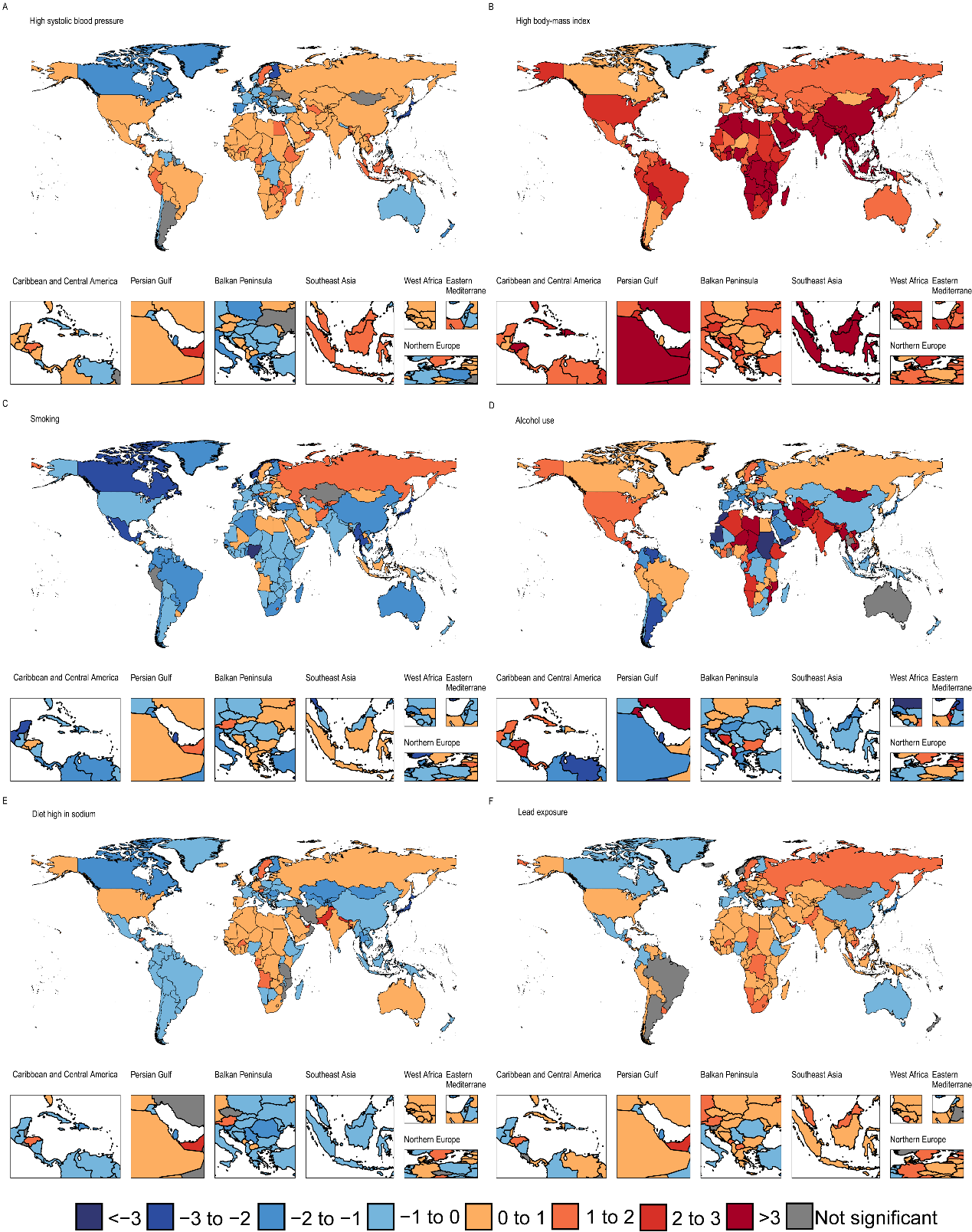


Figure S9: The AAPC of AF/AFL ASDR attributable to specific risk factors for females at 204 countries and territories from 1990 to 2021.

Table S3. The burden of AF/AFL attributable to metabolic, behavioral, and environmental risk factors in 21 GBD regions and 5 SDI levels in 1990 and 2021.

| Region | 1990 | | | | 2021 | | | | AAPC(1990-2021) | |
| --- | --- | --- | --- | --- | --- | --- | --- | --- | --- | --- |
|  | Death(95%UI) | ASMR(95%UI) | DALY(95%UI) | ASDR(95%UI) | Death(95%UI) | ASMR(95%UI) | DALY(95%UI) | ASDR(95%UI) | ASMR(95%CI) | ASDR(95%CI) |
| **High systolic blood pressure** |  |  |  |  |  |  |  |  |  |  |
| East Asia | 4551（1590-7851） | 1.31(0.44-2.38) | 131635（43431-229383） | 23.09（8.02-41.70） | 20383（7168-34125） | 1.28（0.45-2.18） | 512597(172064-883805) | 26.62（9.06-45.99） | -0.07(-0.12--0.02) | 0.34(0.31-0.37) |
| Central Asia | 222（81-363） | 0.60(0.22-0.98) | 8990（2978-14975） | 21.09（7.01-35.22） | 479（176-747） | 0.82（0.30-1.27） | 17657(6129-29135) | 25.42（8.77-41.50） | 0.97（0.82-1.10） | 0.55(0.48-0.61) |
| Oceania | 13（4-23） | 0.89(0.27-1.60) | 452（133-820） | 21.09（6.32-38.47） | 43（13-74） | 1.00（0.30-1.72） | 1522(492-2656) | 25.74（8.17-44.39） | 0.35（0.29-0.39） | 0.58(0.56-0.59) |
| Southeast Asia | 1878（692-3099） | 1.83（0.47-2.13） | 60384（20484-100515） | 31.09（10.65-51.36） | 7427（2645-11942） | 1.74（0.62-2.80） | 200403(68094-329303) | 38.02（12.88-62.55） | 0.95（0.93-0.97） | 0.61(0.60-0.62) |
| Central Europe | 2151（837-3305） | 1.83（0.71-2.83） | 57104（21068-90918） | 41.09（15.41-66.25） | 3620（1307-5850） | 1.48（0.54-2.39） | 87864(29741-142271) | 36.95（12.54-59.78） | -0.67(-0.70--0.64) | -0.38(-0.41--0.37) |
| High-income Asia Pacific | 1742（639-2759） | 1.05（0.38-1.68） | 53151（18348-86727） | 28.09（9.73-45.84） | 4761（1629-8064） | 0.69（0.25-1.17） | 101995(34286-174187) | 18.95（6.30-32.69） | -1.33(-1.41--1.23) | -1.27(-1.32--1.22) |
| Eastern Europe | 2785（1038-4508） | 1.31（0.49-2.13） | 84097（30134-138399） | 32.09（11.89-53.50） | 5341（1979-8418） | 1.47（0.55-2.32） | 131801(46357-214356) | 36.42（12.81-59.19） | 0.42（0.34-0.49） | 0.36(0.33-0.41) |
| Australasia | 495（174-787） | 2.38（0.83-3.81） | 11729（3847-19143） | 51.09（17.02-84.45） | 1200（411-2069） | 1.83（0.63-3.14） | 24614(7998-41987) | 41.39（13.38-70.92） | -0.94（-1.07--0.87） | -0.75(-0.83--0.69) |
| Southern Latin America | 389（136-635） | 1.08（0.38-1.79) | 9492（3111-16068） | 22.09（7.64-38.79） | 1120（391-1836） | 1.20（0.42-1.96） | 22110(7405-36512) | 24.18（8.07-39.98） | 0.37（0.29-0.45） | 0.19(0.14-0.25) |
| High-income North America | 4905（1795-7891） | 1.33(0.49-2.14） | 144080（48204-241299） | 39.09（13.08-65.19） | 11331（3722-19406） | 1.49（0.49-2.53） | 274437(86292-470100) | 38.51（12.02-66.10） | 0.35（0.30-0.39） | -0.05(-0.07--0.03) |
| Western Europe | 11378（4117-17877） | 1.95（0.7-3.07) | 282869（95372-458221） | 46.09（15.82-75.88） | 22330（7887-37015） | 1.71（0.61-2.83） | 445232(146353-745694) | 40.24（13.31-67.17） | -0.40(-0.45--0.36) | -0.49(-0.51--0.48) |
| Andean Latin America | 133（46-239） | 0.82(0.28-1.48) | 3376（1082-6171） | 18.09（6.13-34.51） | 594（202-995） | 1.09（0.37-1.83） | 14935(4853-25521) | 26.63（8.67-45.61） | 0.94（0.82-1.03） | 1.10(1.04-1.15) |
| Caribbean | 299（104-487） | 1.51(0.52-2.47) | 7662（2485-13041） | 33.09（10.72-55.83） | 760（278-1256） | 1.36（0.50-2.25） | 17751(5991-30233) | 32.49（10.98-55.26） | -0.33(-0.38--0.28) | -0.04(-0.07--0.01) |
| Central Latin America | 787（281-1282） | 1.40(0.49-2.26) | 22212（7463-37257） | 32.09（10.85-54.01） | 3230（1215-5205） | 1.43（0.54-2.30） | 83283(27829-138266) | 35.24（11.81-58.62） | 0.07（0.02-0.12） | 0.28(0.25-0.31) |
| Tropical Latin America | 804（296-1309） | 1.46(0.53-2.26） | 26394（8900-44495） | 35.09（12.09-60.80） | 3481（1208-5832） | 1.47（0.51-2.46） | 90906(29923-153999) | 36.77（12.10-62.38） | 0.06（0.00-0.11） | 0.08(0.05-0.10) |
| North Africa and Middle East | 1005（351-1690） | 1.03(0.36-1.75) | 26088（8941-43868） | 21.09（7.36-35.34） | 3389（1170-5411） | 1.17（0.40-1.87） | 80510(27034-133277) | 23.05（7.70-37.98） | 0.49（0.42-0.53） | 0.31(0.28-0.33) |
| South Asia | 2113（603-4068） | 0.65(0.19-1.26) | 84749（28035-148758） | 19.09（6.58-35.02） | 10592（3441-18323） | 1.07（0.35-1.87） | 311893(101195-528420) | 25.73（8.43-43.53） | 1.65（1.57-1.73） | 0.91(0.87-0.95) |
| Central Sub-Saharan Africa | 144（45-264） | 1.34(0.40-2.43) | 4609（1552-7948） | 29.09（9.91-50.77） | 403（119-735） | 1.43（0.42-2.70） | 11379(3517-19831) | 30.02（9.41-52.45） | 0.23（0.21-0.26） | 0.03(0.01-0.05) |
| Southern Sub-Saharan Africa | 169（60-284） | 0.93(0.32-1.57) | 5626（1904-9417） | 25.09（8.41-42.01） | 486（171-773） | 1.36（0.48-2.20） | 14247(4659-23530) | 30.96（10.28-51.11） | 1.29（1.20-1.36） | 0.70(0.65-0.74) |
| Eastern Sub-Saharan Africa | 302（87-580） | 0.75(0.22-1.48) | 10034（3069-18073） | 18.09（5.61-32.38） | 898（253-1705） | 0.91（0.26-1.73） | 29423(9373-50935) | 22.48（7.04-39.27） | 0.64（0.62-0.67） | 0.71(0.70-0.72) |
| Western Sub-Saharan Africa | 590（204-1020） | 1.23(0.43-2.11) | 14595（5075-24729） | 23.09（8.15-39.92） | 1554（573-2490） | 1.50（0.55-2.42） | 40025(13366-64680) | 29.18（9.77-47.13） | 0.65（0.63-0.67） | 0.75(0.74-0.77) |
| High-middle SDI | 9225（3422-14678） | 1.38(0.51-2.21) | 266609（92714-433422） | 31.09（11.15-51.26） | 24600（8988-40902） | 1.33（0.48-2.20） | 581873(198781-978419) | 29.90（10.26-50.31） | -0.11(-0.16--0.06) | -0.19(-0.22--0.16) |
| Low-middle SDI | 2971（960-5155） | 0.86(0.29-1.48) | 103235（34280-175459） | 22.09（7.55-38.32） | 12001（4258-19705） | 1.24（0.44-2.06） | 337217(114167-549421) | 28.68（9.72-46.61） | 1.22（1.15-1.30） | 0.83(0.79-0.86) |
| Low SDI | 973（280-1853） | 0.83(0.24-1.56) | 33178（10934-58233） | 20.09（6.79-35.42） | 3052（989-5434） | 1.06（0.35-1.92） | 91332(30104-158327) | 24.06（7.97-41.54） | 0.83（0.77-0.90） | 0.57(0.54-0.60) |
| High SDI | 17545（6429-27765） | 1.65(0.60-2.62) | 454994（154804-739649） | 40.09（13.90-66.38） | 37812（13104-63083） | 1.40（0.49-2.33） | 814734(266923-1379270) | 34.24（11.24-58.06） | -0.53(-0.56--0.50) | -0.57(-0.58--0.56) |
| Middle SDI | 6081（2219-9931） | 1.14(0.42-1.88) | 189763（64803-320745） | 25.09（8.66-42.66） | 25844（9119-42782） | 1.30（0.46-2.15） | 686700(228689-1149553) | 29.35（9.75-49.10） | 0.43（0.41-0.46） | 0.46(0.45-0.48) |
| **High body-mass index** |  |  |  |  |  |  |  |  |  |  |
| East Asia | 58（13-133） | 0.01(0.00-0.03) | 2358（578-4981） | 0.33（0.07-0.73） | 2544（995-4446） | 0.16（0.06-0.28） | 78959（30814-135780） | 3.90（1.53-6.67） | 8.28(8.18-8.37) | 8.22(8.18-8.26) |
| Oceania | 3（1-6） | 0.16(0.06-0.35) | 134（52-251） | 4.80（1.80-9.46） | 14（6-25） | 0.27（0.12-0.49） | 572（237-1011） | 8.11（3.35-14.25） | 1.65(1.58-1.70) | 1.68(1.65-1.70) |
| Southeast Asia | 22（4-47） | 0.01(0.00-0.03) | 1289（366-2504） | 0.49（0.13-1.00） | 508（194-902） | 0.11（0.04-0.20） | 19002（7629-32056） | 3.17（1.24-5.43） | 7.76(7.69-7.81) | 6.17(6.16-6.19) |
| Central Europe | 570（246-977） | 0.48(0.21-0.82) | 16331（6519-28128） | 11.70（4.65-20.00） | 1462（651-2582） | 0.60（0.27-1.06） | 36521（15070-64445） | 15.44（6.38-27.38） | 0.73(0.70-0.75) | 0.88(0.86-0.90) |
| Central Asia | 60（25-102） | 0.16(0.07-0.28) | 2559（1018-4384） | 6.00（2.40-10.22） | 163（71-276） | 0.27（0.12-0.46） | 6545（2672-11650） | 9.09（3.67-16.08） | 1.64(1.46-1.80) | 1.32(1.24-1.38) |
| High-income Asia Pacific | 31（9-69） | 0.02(0.00-0.04) | 1255（355-2644） | 0.64（0.18-1.36） | 342（122-652） | 0.05（0.02-0.10） | 8524（3032-15689） | 1.77（0.66-3.25） | 3.38(3.29-3.48) | 3.36(3.32-3.41) |
| Eastern Europe | 784（331-1304） | 0.36(0.16-0.60) | 24533（10151-40575） | 9.48（3.87-15.64） | 2283（980-3917） | 0.63（0.27-1.09） | 59342（25169-103404） | 16.43（6.97-28.51） | 1.83(1.74-1.92) | 1.82(1.79-1.86) |
| Australasia | 97（38-182） | 0.46(0.18-0.87) | 2627（1016-4866） | 11.55（4.51-21.35） | 563（233-1041） | 0.86（0.36-1.59） | 12414（5304-21747） | 21.40（9.07-37.72） | 1.91(1.83-1.98) | 1.97(1.90-2.04) |
| Western Europe | 2049（862-3497） | 0.35(0.15-0.60) | 53706（20725-90353） | 9.02（3.46-15.21） | 7145（2949-12840） | 0.55（0.23-1.00） | 148760（58925-264448） | 13.85（5.58-24.77） | 1.47(1.43-1.50) | 1.40(1.38-1.42) |
| Southern Latin America | 96（40-177） | 0.26(0.10-0.47) | 2901（1135-5268） | 6.69（2.60-12.02） | 417（176-758） | 0.45（0.19-0.81） | 9416（3834-16721） | 10.44（4.26-18.55） | 1.88(1.82-1.94) | 1.45(1.41-1.49) |
| Caribbean | 38（16-62） | 0.18(0.08-0.30) | 1268（524-2160） | 5.22（2.17-8.86） | 222（99-363） | 0.39（0.18-0.65） | 5958（2399-10212） | 10.89（4.38-18.69） | 2.49(2.42-2.55) | 2.40(2.38-2.43) |
| High-income North America | 1240（503-2412） | 0.34(0.14-0.66) | 42432（16693-79113） | 11.79（4.64-22.02） | 5732（2425-9904） | 0.76（0.32-1.31） | 165664（70084-282995） | 23.85（10.09-40.69） | 2.64(2.59-2.67) | 2.29(2.27-2.30) |
| Andean Latin America | 20（8-38） | 0.12(0.04-0.22) | 813（300-1537） | 4.13（1.51-7.63） | 182（73-330） | 0.33（0.13-0.60） | 5973（2405-10708） | 10.37（4.16-18.54） | 3.42(3.30-3.54) | 3.01(2.94-3.07) |
| Tropical Latin America | 115（44-208） | 0.19(0.07-0.35) | 5157（1987-9372） | 6.21（2.35-11.29） | 1266（509-2201） | 0.53（0.21-0.92） | 36495（14653-64474） | 14.59（5.85-25.88） | 3.46(3.41-3.51) | 2.81(2.79-2.83) |
| Central Latin America | 156（63-286） | 0.26(0.10-0.47) | 5618（2199-10138） | 7.53（2.93-13.50） | 1244（551-2211） | 0.54（0.24-0.96） | 36267（15390-64769） | 15.03（6.36-26.72） | 2.45(2.37-2.52) | 2.24(2.22-2.27) |
| North Africa and Middle East | 249（99-441） | 0.24(0.09-0.43) | 7059（2801-12196） | 5.30（2.09-9.21） | 1660（703-2811） | 0.56（0.24-0.96） | 42718（18785-73469） | 11.69（5.10-20.18） | 2.79(2.72-2.86) | 2.62(2.58-2.65) |
| South Asia | 30（8-66） | 0.01(0.00-0.02) | 1577（489-3091） | 0.30（0.08-0.61） | 823（345-1507） | 0.08（0.03-0.15） | 30488（11896-53295） | 2.30（0.90-4.10） | 7.81(7.72-7.90) | 6.79(6.74-6.84) |
| Eastern Sub-Saharan Africa | 8（2-18） | 0.02(0.00-0.04) | 392（121-790） | 0.56（0.17-1.14） | 91（34-171） | 0.08（0.03-0.15） | 3726（1474-6591） | 2.47（0.96-4.37） | 5.28(5.26-5.31) | 4.89(4.87-4.91) |
| Southern Sub-Saharan Africa | 36（16-63） | 0.19(0.08-0.34) | 1444（597-2409） | 6.03（2.47-10.09） | 207（94-342） | 0.57（0.26-0.94） | 6615（2824-11345） | 13.80（5.94-23.50） | 3.58(3.47-3.67) | 2.73(2.67-2.79) |
| Central Sub-Saharan Africa | 5（2-11） | 0.03(0.01-0.08) | 198（68-389） | 0.99（0.34-1.99） | 67（25-136） | 0.21（0.07-0.42） | 2220（807-4058） | 5.08（1.85-9.34） | 6.04(6.01-6.07) | 5.43(5.41-5.45) |
| Western Sub-Saharan Africa | 50（18-93） | 0.10(0.04-0.19) | 1380（542-2412） | 2.05（0.79-3.74） | 305（127-522） | 0.30（0.12-0.51） | 8394（3406-14015） | 5.90（2.42-9.83） | 3.48(3.46-3.51) | 3.46(3.44-3.47) |
| High SDI | 3342（1379-5954） | 0.31(0.13-0.56) | 95785（37100-167987） | 8.64（3.36-15.20） | 13036（5608-22825） | 0.49（0.21-0.86） | 324193（134750-566662） | 14.21（5.95-24.65） | 1.46(1.44-1.48) | 1.61(1.60-1.62) |
| Low SDI | 30（9-63） | 0.02(0.01-0.04) | 1236（416-2329） | 0.62（0.20-1.19） | 265（105-485） | 0.08（0.03-0.15） | 9595（3655-16707） | 2.21（0.85-3.91） | 4.50(4.47-4.53) | 4.22(4.20-4.23) |
| High-middle SDI | 1704（712-2821） | 0.25(0.10-0.41) | 53742（21649-91086） | 6.20（2.52-10.44） | 7209（3108-12447） | 0.39（0.17-0.67） | 182168（74547-317112） | 9.33（3.81-16.20） | 1.48(1.43-1.52) | 1.34(1.31-1.36) |
| Middle SDI | 436（173-749） | 0.08(0.03-0.13) | 16889（6475-28280） | 2.01（0.77-3.37） | 4907（2166-8209） | 0.24（0.11-0.41） | 150960（62576-256705） | 6.18（2.57-10.42） | 3.80(3.75-3.84) | 3.70(3.68-3.72) |
| Low-middle SDI | 197（77-350） | 0.06(0.02-0.10) | 7015（2799-11742） | 1.42（0.55-2.35） | 1782（757-2955） | 0.18（0.08-0.30） | 56694（23028-93081） | 4.57（1.84-7.43） | 3.93(3.84-4.00) | 3.85(3.82-3.89) |
| **Smoking** |  |  |  |  |  |  |  |  |  |  |
| Oceania | 337（194-513） | 0.15(0.08-0.23) | 20106（115-306） | 6.11(3.53-9.25) | 9（5-14） | 0.13（0.07-0.20） | 486（270-725） | 5.54（3.09-8.25） | -0.46(-0.48--0.43) | -0.32(-0.34--0.30) |
| Southeast Asia | 967（539-1478） | 0.20(0.11-0.32) | 15613（9105-23513） | 6.82(4.00-10.25) | 876（524-1279） | 0.19（0.11-0.28） | 36781（21532-53737） | 5.98（3.50-8.81） | -0.30(-0.33--0.26) | -0.43(-0.44--0.41) |
| East Asia | 4（2-6） | 0.21(0.12-0.33) | 45834（27111-68134） | 6.16(3.66-9.10) | 2769（1534-4226） | 0.16（0.09-0.24） | 116915（65111-175945） | 5.48（3.03-8.21） | -0.95(-1.00--0.90) | -0.41(-0.44--0.39) |
| Central Europe | 225（138-321） | 0.17(0.10-0.24) | 99237（5836-14937） | 6.68(3.91-9.95) | 296（171-434） | 0.13（0.07-0.19） | 2866（1582-4314） | 3.43（1.92-5.16） | -0.89(-0.93--0.85) | -0.47(-0.51--0.44) |
| Central Asia | 19（11-26） | 0.04(0.03-0.06) | 1402（794-2122） | 2.93(1.65-4.44) | 46（27-66） | 0.07（0.04-0.10） | 12406（7248-18516） | 5.80（3.39-8.62） | 1.35(1.17-1.49) | 0.51(0.44-0.56) |
| High-income Asia Pacific | 293（181-419） | 0.16(0.10-0.23) | 13641（8087-20241） | 6.75(4.00-10.07) | 472（271-722） | 0.08（0.05-0.12） | 15879（9153-24025） | 3.90（2.23-5.86） | -2.15(-2.21--2.09) | -1.74(-1.77--1.72) |
| Eastern Europe | 194（111-295） | 0.08(0.05-0.12) | 12252（6978-18452） | 4.40(2.51-6.58) | 345（208-499） | 0.10（0.06-0.14） | 17791（10528-26645） | 5.19（3.05-7.78） | 0.79(0.72-0.88) | 0.56(0.53-0.59) |
| Australasia | 41（25-61） | 0.18(0.11-0.27) | 1814（1069-2694） | 7.86(4.60-11.62) | 72（41-110） | 0.12（0.07-0.18） | 2467（1394-3835） | 4.81（2.73-7.41） | -1.42(-1.52--1.36) | -1.58(-1.67--1.53) |
| Western Europe | 1181（711-1737） | 0.20(0.12-0.30) | 45915（26504-70715） | 8.12(4.73-12.40) | 1632（944-2488） | 0.14（0.08-0.21） | 55242（31740-84788） | 6.23（3.61-9.39） | -1.11(-1.14--1.08) | -0.84(-0.86--0.82) |
| Southern Latin America | 33（20-49） | 0.08(0.05-0.12) | 1779（1036-2679） | 3.84(2.24-5.81) | 60（36-91） | 0.07（0.04-0.10） | 2541（1503-3767） | 2.99（1.77-4.47） | -0.41(-0.49--0.34) | -0.82(-0.84--0.78) |
| Caribbean | 36（21-54） | 0.17(0.10-0.25) | 1442（840-2182） | 5.76(3.34-8.72) | 67（38-102） | 0.12（0.07-0.18） | 2412（1369-3661） | 4.44（2.52-6.74） | -0.96(-1.03--0.90) | -0.81(-0.83--0.79) |
| High-income North America | 534（318-799） | 0.15(0.09-0.22) | 25126（14233-39126） | 7.26(4.13-11.14) | 1036（596-1573） | 0.15（0.08-0.22） | 40735（23448-61789） | 6.27（3.63-9.40） | -0.04(-0.06--0.01) | -0.47(-0.48--0.45) |
| Andean Latin America | 17（10-25） | 0.10(0.05-0.15) | 6687（387-1007） | 3.36(1.93-5.09) | 38（21-59） | 0.07（0.04-0.11） | 1630（895-2490） | 2.76（1.52-4.21） | -1.16(-1.25--1.10) | -0.61(-0.65--0.58) |
| Tropical Latin America | 149（91-217） | 0.23(0.14-0.35) | 78008（4516-11808） | 8.95(5.22-13.50) | 299（174-451） | 0.12（0.07-0.19） | 12561（7107-19351） | 4.89（2.76-7.55） | -2.01(-2.06--1.96) | -1.91(-1.95--1.88) |
| Central Latin America | 104（63-151） | 0.17(0.10-0.25) | 4397（2577-6567） | 5.61(3.27-8.36) | 197（117-293） | 0.08（0.05-0.13） | 8167（4601-12629） | 3.27（1.85-5.06） | -2.21(-2.26--2.16) | -1.74(-1.76--1.71) |
| North Africa and Middle East | 109（60-166） | 0.09(0.05-0.14) | 4413（2492-6763） | 2.88(1.64-4.39) | 281（162-420） | 0.09（0.05-0.13） | 11190（6492-16644） | 2.64（1.53-3.98） | -0.21(-0.25--0.18) | -0.29(-0.30--0.27) |
| South Asia | 419（204-671） | 0.11(0.05-0.19) | 21854（11853-33654） | 4.37(2.36-6.71) | 1335（777-1988） | 0.13（0.07-0.19） | 47969（27497-71346） | 3.62（2.10-5.34） | 0.33(-0.25-0.41) | -0.58(-0.62--0.53) |
| Eastern Sub-Saharan Africa | 47（21-75） | 0.10(0.05-0.16) | 1898（1001-2968） | 2.85(1.50-4.48) | 82（38-140） | 0.07（0.03-0.12） | 3529（1870-5714） | 2.19（1.15-3.49） | -1.17(-1.21--1.14) | -0.84(-0.85--0.82) |
| Southern Sub-Saharan Africa | 26（15-39） | 0.12(0.07-0.19) | 1208（697-1778） | 4.65(2.71-6.96) | 37（22-55） | 0.08（0.05-0.12） | 1770（1055-2664） | 3.04（1.79-4.56） | -1.33(-1.40--1.25) | -1.36(-1.40--1.32) |
| Central Sub-Saharan Africa | 10（5-17） | 0.06(0.03-0.10) | 45419（239-719） | 2.06(1.10-3.21) | 23（12-38） | 0.05（0.03-0.09） | 1037（582-1600） | 1.82（1.02-2.82） | -0.44(-0.47--0.41) | -0.39(-0.41--0.36) |
| Western Sub-Saharan Africa | 26（14-40） | 0.05(0.03-0.08) | 1017（545-1567） | 1.32(0.72-2.03) | 41（22-62） | 0.03（0.02-0.05） | 1838（992-2826） | 1.01（0.55-1.55） | -1.25(-1.28--1.23) | -0.87(-0.88--0.86) |
| High SDI | 1997（1203-2928） | 0.18(0.11-0.27) | 822028（47784-125028） | 7.53(4.39-11.39) | 3201（1848-4790） | 0.13（0.08-0.19） | 114875（66836-172129） | 5.65（3.28-8.43） | -1.07(-1.09--1.05) | -0.92(-0.93--0.91) |
| Low SDI | 121（56-192） | 0.08(0.04-0.13) | 5740（3155-8820） | 2.88(1.59-4.46) | 285（152-457） | 0.09（0.04-0.14） | 11280（6369-17288） | 2.51（1.42-3.85） | 0.04(-0.04-0.13) | -0.45(-0.47--0.42) |
| High-middle SDI | 1070（628-1537） | 0.14(0.08-0.20) | 53229（31102-79329） | 5.53(3.24-8.25) | 2367（1344-3493） | 0.12（0.07-0.18） | 99329（57533-147760） | 5.03（2.90-7.48） | -0.30(-0.36--0.25) | -0.33(-0.35--0.30) |
| Middle SDI | 1079（627-1569） | 0.17(0.09-0.25) | 52270（30599-77170） | 5.61(3.29-8.28) | 2724（1588-4034） | 0.13（0.07-0.19） | 116419（66895-173374） | 4.46（2.57-6.65） | -0.92(-0.95--0.90) | -0.75(-0.77--0.72) |
| Low-middle SDI | 497（265-767） | 0.12(0.06-0.20) | 24947（13852-38347） | 4.59(2.57-7.04) | 1426（843-2093） | 0.13（0.08-0.20） | 53909（30795-79712） | 4.10（2.36-6.04） | 0.28(0.21-0.35) | -0.36(-0.40--0.33) |
| **Alcohol use** |  |  |  |  |  |  |  |  |  |  |
| Oceania | 1（0-1） | 0.03(0.01-0.04) | 26（14-37） | 0.90(0.49-1.26) | 2（1-2） | 0.03（0.01-0.04） | 68（41-99） | 0.93（0.54-1.36） | 0.16(0.11-0.20) | (0.10(0.07-0.12) |
| Southeast Asia | 52（35-52） | 0.03(0.02-0.05) | 2294（1528-3173） | 1.01(0.68-1.38) | 359（264-359） | 0.08（0.06-0.11） | 13012（9234-17513） | 2.21（1.55-2.99） | 3.01(2.96-3.06) | (2.58(2.55-2.61) |
| East Asia | 371（236-371） | 0.08(0.05-0.11) | 17763（11923-24330） | 2.37(1.61-3.22) | 1573（1097-1573） | 0.09（0.06-0.13） | 63244（42860-86321） | 2.98（2.03-4.05） | 0.43(0.37-0.49) | (0.71(0.67-0.75) |
| Central Europe | 280（175-280） | 0.22(0.14-0.29) | 9682（6507-12939） | 6.72(4.49-8.99) | 557（380-557） | 0.23（0.16-0.30） | 17468（12320-22987） | 2.30（1.44-3.24） | 0.12(0.07-0.16) | 0.41(0.39-0.43) |
| Central Asia | 12（7-12） | 0.03(0.02-0.05) | 806（488-1146） | 1.73(1.02-2.49) | 34（21-34） | 0.05（0.03-0.08） | 1843（1178-2569） | 7.63（5.38-10.04） | 1.75(1.50-1.94) | (0.89(0.80-0.95) |
| High-income Asia Pacific | 268（197-268） | 0.16(0.11-0.20) | 10303（7092-13856） | 5.25(3.62-7.02) | 718（501-718） | 0.11（0.08-0.15） | 19195（13784-25642） | 4.16（2.95-5.55） | -1.01(-1.07--0.94) | (-0.76(-0.79--0.72) |
| Eastern Europe | 214（110-214） | 0.09(0.04-0.14) | 10782（6435-14935） | 3.89(2.30-5.43) | 405（218-405） | 0.11（0.06-0.17） | 16604（10202-23214） | 4.73（2.95-6.59） | 0.80(0.72-0.90) | (0.65(0.60-0.70) |
| Australasia | 89（48-89） | 0.42(0.22-0.62) | 2715（1613-3869） | 11.87(7.03-16.88) | 278（187-278） | 0.43（0.30-0.57） | 6813（4696-8969） | 12.01（8.31-15.77） | 0.04(-0.06-0.13) | 0.02(-0.08-0.10) |
| Southern Latin America | 76（54-76） | 0.20(0.14-0.26) | 2494（1750-3334） | 5.56(3.23-8.12) | 143（107-143） | 0.15（0.12-0.20） | 3752（2728-4939） | 4.20（3.06-5.55） | -0.81(-0.88--0.75) | (-0.98(-1.03--0.93) |
| Western Europe | 2125（1389-2125） | 0.36(0.23-0.47) | 64686（44057-86762） | 11.00(7.54-14.75) | 4589（3384-4589） | 0.36（0.27-0.46） | 109934（80260-141534） | 10.76（7.84-13.83） | 0.02(-0.02-0.06) | (-0.06(-0.08--0.04) |
| High-income North America | 478（261-478） | 0.13(0.07-0.20) | 19699（11203-29001） |  | 1843（1242-1843） | 0.25（0.17-0.33） | 58764（41590-76737） | 8.63（6.13-11.29） | 2.13(2.10-2.15) | (1.43(1.41-1.45) |
| Caribbean | 20（13-20） | 0.09(0.06-0.13) | 766（510-1053） | 3.08(2.05-4.24) | 57（41-57） | 0.10（0.07-0.14） | 1841（1291-2457） | 3.39（2.38-4.52） | 0.30(0.25-0.35) | 0.34(0.32-0.37) |
| Andean Latin America | 16（9-16） | 0.09(0.05-0.14) | 582（362-847） | 2.98(1.84-4.37) | 49（32-49） | 0.09（0.06-0.13） | 1966（1271-2754） | 3.37（2.17-4.71） | 0.00(-0.09-0.08) | 0.44(0.40-0.49) |
| Central Latin America | 61（45-61） | 0.10(0.07-0.13) | 2558（1772-3437） | 3.33(2.31-4.45) | 201（144-201） | 0.09（0.06-0.11） | 7661（5283-10358） | 3.10（2.15-4.19） | -0.49(-0.55--0.42) | -0.23(-0.24--0.21) |
| Tropical Latin America | 61（45-61） | 0.10(0.07-0.13) | 2992（1979-4157） | 3.53(2.33-4.86) | 290（214-290） | 0.12（0.09-0.16） | 10746（7611-14565） | 4.22（3.00-5.73） | 0.65(0.57-0.71) | (0.59(0.55-0.63) |
| North Africa and Middle East | 8（5-8） | 0.01(0.00-0.01) | 321（202-451） | 0.21(0.13-0.30) | 24（15-24） | 0.01（0.00-0.01） | 902（586-1229） | 0.22（0.14-0.30） | 0.32(0.26-0.36) | (0.16(0.13-0.19) |
| South Asia | 60（25-60） | 0.02(0.01-0.03) | 3337（1544-5330） | 0.64(0.29-1.02) | 478（304-478） | 0.04（0.03-0.06） | 18324（11298-25432） | 1.36（0.83-1.87） | 3.49(3.37-3.61) | (2.49(2.45-2.54) |
| Eastern Sub-Saharan Africa | 29（14-29） | 0.06(0.03-0.10) | 1092（627-1601） | 1.74(1.02-2.53) | 83（46-83） | 0.08（0.04-0.12） | 3209（2006-4680） | 2.18（1.35-3.22） | 0.59(0.56-0.62) | (0.76(0.74-0.79) |
| Southern Sub-Saharan Africa | 20（14-20） | 0.10(0.07-0.15) | 827（561-1125） | 3.38(2.30-4.59) | 47（33-47） | 0.12（0.08-0.16） | 1778（1235-2343） | 3.44（2.41-4.48） | 0.48(0.43-0.54) | (0.05(0.03-0.08) |
| Central Sub-Saharan Africa | 10（5-10） | 0.08(0.04-0.13) | 361（168-569） | 2.05(1.02-3.19) | 30（17-30） | 0.09（0.05-0.15） | 1050（597-1556） | 2.32（1.34-3.47） | 0.39(0.36-0.43) | (0.42(0.38-0.46) |
| Western Sub-Saharan Africa | 55（33-55） | 0.11(0.06-0.16) | 1616（1047-2256） | 2.36(1.51-3.29) | 149（97-149） | 0.14（0.09-0.19） | 4524（3122-6120） | 3.03（2.08-4.09） | 0.76(0.74-0.79) | (0.82(0.80-0.84) |
| High-middle SDI | 1074（730-1074） | 0.15(0.10-0.20) | 44049（28978-59127） | 4.83(3.19-6.45) | 2622（1896-2622） | 0.14（0.10-0.18） | 85588（60969-112185） | 4.35（3.11-5.69） | -0.14(-0.19--0.09) | (-0.34(-0.37--0.32) |
| High SDI | 2672（1769-2672） | 0.25(0.16-0.33) | 86191（57933-114674） | 7.79(5.24-10.31) | 6814（5056-6814） | 0.26（0.20-0.33） | 182363（133910-237186） | 8.20（5.97-10.62） | 0.19(0.16-0.22) | (0.17(0.16-0.19) |
| Low SDI | 56（28-56） | 0.04(0.02-0.06) | 2138（1231-3087） | 1.13(0.64-1.62) | 188（113-188） | 0.06（0.04-0.09） | 6882（4395-9725） | 1.60（1.02-2.27） | 1.27(1.22-1.32) | (1.18(1.15-1.21) |
| Middle SDI | 390（281-390） | 0.06(0.04-0.08) | 18100（12660-24570） | 1.98(1.40-2.66) | 1739（1312-1739） | 0.08（0.06-0.11） | 67542（48172-89959） | 2.64（1.90-3.55） | 0.92(0.89-0.95) | (0.93(0.91-0.95) |
| Low-middle SDI | 110（69-110） | 0.03(0.02-0.04) | 4985（3112-7174） | 0.94(0.60-1.35) | 530（375-530） | 0.05（0.04-0.07） | 19885（13532-26506） | 1.51（1.04-2.01） | 1.81(1.74-1.89) | (1.59(1.56-1.63) |
| **Diet high in sodium** |  |  |  |  |  |  |  |  |  |  |
| East Asia | 1042（178-2617） | 0.25(0.03-0.66) | 17763（11923-24330） | 5.89(1.04-14.20) | 3273(420-8905) | 0.19（0.02-0.54） | 112424（20389-270393） | 5.41（0.94-13.39） | -0.83(-0.89--0.78) | -0.30(-0.34--0.26) |
| Oceania | 3（0-7） | 0.19(0.02-0.52) | 26（14-37） | 4.46(0.41-12.24) | 7(0-20) | 0.17（0.01-0.49） | 234（19-692） | 4.31（0.33-12.62） | -0.33(-0.36--0.30) | -0.11(-0.13--0.09) |
| Southeast Asia | 309（41-796） | 0.20(0.02-0.52) | 2294（1528-3173） | 5.70(0.84-13.93) | 887(71-2586) | 0.20（0.01-0.60） | 27819（3073-77652） | 5.00（0.53-14.33） | 0.02(-0.01-0.04) | -0.43(-0.44--0.41) |
| Central Europe | 370（70-868） | 0.31(0.06-0.75) | 806（488-1146） | 2.63(0.23-7.61) | 659(101-1585) | 0.27（0.04-0.65） | 15892（2587-38795） | 6.69（1.10-16.39） | -0.49(-0.54--0.46) | -0.32(-0.34--0.30) |
| Central Asia | 29（3-82） | 0.08(0.01-0.23) | 9682（6507-12939） | 7.39(1.40-17.34) | 43(2-133) | 0.08（0.00-0.23） | 1388（54-4810） | 2.07（0.08-7.01） | -0.24(-0.42--0.08) | -0.76(-0.84--0.67) |
| High-income Asia Pacific | 280（39-684） | 0.16(0.02-0.41) | 10303（7092-13856） | 4.98(0.84-12.02) | 729(59-2051) | 0.11（0.01-0.30） | 16505（1534-44891） | 3.18（0.32-8.57） | -1.35(-1.42--1.28) | -1.40(-1.44--1.36) |
| Eastern Europe | 135（2-558） | 0.06(0.00-0.26) | 10782（6435-14935） | 1.84(0.06-7.11) | 271(5-1096) | 0.07（0.00-0.30） | 8070（293-29917） | 2.25（0.09-8.31） | 0.65(0.56-0.73) | 0.68(0.63-0.73) |
| Australasia | 12（0-67） | 0.06(0.00-0.32) | 2715（1613-3869） | 1.53(0.00-7.41) | 38(0-205) | 0.06（0.00-0.31） | 865（2-4273） | 1.53（0.00-7.45） | -0.03(-0.12-0.03) | -0.04(-0.11-0.01) |
| Southern Latin America | 38（1-129） | 0.10(0.00-0.35) | 2494（1750-3334） | 2.38(0.05-8.19) | 90(2-310) | 0.10（0.00-0.33） | 1920（36-6512） | 2.11（0.04-7.18） | -0.22(-0.31--0.15) | -0.35(-0.40--0.30) |
| Western Europe | 441（3-1972） | 0.08(0.00-0.34) | 64686（44057-86762） | 2.09(0.04-8.66) | 1086(7-4735) | 0.08（0.00-0.37） | 24483（291-98560） | 2.35（0.03-9.18） | 0.36(0.32-0.40) | 0.38(0.36-0.41) |
| High-income North America | 204（0-913） | 0.06(0.00-0.25) | 19699（11203-29001） | 1.76(0.01-7.77) | 679(5-2754) | 0.09（0.00-0.37） | 20971（275-80569） | 3.03（0.04-11.48） | 1.63(1.57-1.68) | 1.77(1.75-1.79) |
| Caribbean | 26（0-97） | 0.14(0.00-0.51) | 766（510-1053） | 2.69(0.02-10.19) | 56(0-221) | 0.10（0.00-0.39） | 1190（7-4885） | 2.17（0.01-8.93） | -1.06(-1.12--1.01) | -0.71(-0.74--0.68) |
| Andean Latin America | 23（1-76） | 0.14(0.00-0.48) | 582（362-847） | 3.28(0.08-10.87) | 60(1-204) | 0.11（0.00-0.37） | 1605（34-5443） | 2.85（0.06-9.67） | -0.85(-0.94--0.78) | -0.44(-0.50--0.39) |
| Central Latin America | 88（5-288） | 0.16(0.01-0.51) | 2558（1772-3437） | 3.71(0.26-12.19) | 315(22-1015) | 0.14（0.01-0.45） | 8480（628-26707） | 3.57（0.26-11.24） | -0.42(-0.46--0.37) | -0.15(-0.17--0.12) |
| Tropical Latin America | 86（3-279） | 0.16(0.01-0.50) | 2992（1979-4157） | 3.87(0.15-12.64) | 325(9-1084) | 0.14（0.00-0.46） | 8661（243-29175） | 3.50（0.10-11.78） | -0.40(-0.46--0.34) | -0.32(-0.35--0.29) |
| North Africa and Middle East | 15（0-95） | 0.01(0.00-0.09) | 321（202-451） | 0.37(0.00-2.24) | 47(0-311) | 0.02（0.00-0.10） | 1440（0-8836） | 0.38（0.00-2.36） | 0.28(0.25-0.32) | 0.12(0.10-0.14) |
| South Asia | 160（2-620） | 0.05(0.00-0.19) | 3337（1544-5330） | 1.54(0.02-5.82) | 778(13-3070) | 0.08（0.00-0.31） | 25024（634-91378） | 1.99（0.04-7.37） | 1.62(1.53-1.72) | 0.88(0.81-0.94) |
| Eastern Sub-Saharan Africa | 52（3-162） | 0.13(0.01-0.42) | 1092（627-1601） | 3.00(0.20-8.96) | 99(4-332) | 0.10（0.00-0.35） | 3053（123-10099） | 2.45（0.10-7.93） | -0.63(-0.65--0.62) | -0.65(-0.66--0.64) |
| Southern Sub-Saharan Africa | 5（0-26） | 0.03(0.00-0.14) | 827（561-1125） | 0.82(0.00-3.99) | 12(0-63) | 0.03（0.00-0.18） | 381（0-1891） | 0.80（0.00-4.00） | 0.71(0.64-0.77) | -0.04(-0.08-0.00) |
| Central Sub-Saharan Africa | 3（0-18） | 0.04(0.00-0.19) | 361（168-569） | 0.73(0.00-3.79) | 12(0-66) | 0.05（0.00-0.24） | 315（0-1738） | 0.90（0.00-4.76） | 0.85(0.83-0.87) | 0.67(0.66-0.68) |
| Western Sub-Saharan Africa | 28（0-130） | 0.06(0.00-0.27) | 1616（1047-2256） | 1.13(0.00-5.08) | 66(0-299) | 0.06（0.00-0.29） | 1736（4-7406） | 1.26（0.00-5.34） | 0.25(0.23-0.28) | 0.33(0.31-0.35) |
| High-middle SDI | 917（129-2607） | 0.13(0.02-0.37) | 44049（28978-59127） | 3.57(0.55-9.70) | 2493(274-7306) | 0.13（0.01-0.39） | 76422（11678-209105） | 3.87（0.59-10.65） | 0.15(0.09-0.21) | 0.23(0.19-0.26) |
| High SDI | 1112（89-3813） | 0.10(0.01-0.35) | 86191（57933-114674） | 2.89(0.27-9.55) | 2878(152-9912) | 0.11（0.01-0.37） | 71831（4551-239530） | 3.15（0.21-10.35） | 0.19(0.17-0.21) | 0.28(0.26-0.29) |
| Low SDI | 85（3-309） | 0.07(0.00-0.26) | 2138（1231-3087） | 1.74(0.07-6.25) | 222(5-836) | 0.08（0.00-0.29） | 6905（165-25630） | 1.81（0.04-6.71） | 0.43(0.37-0.49) | 0.15(0.12-0.18) |
| Middle SDI | 954（130-2596） | 0.16(0.02-0.46) | 18100（12660-24570） | 4.30(0.63-11.20) | 2948(294-8792) | 0.14（0.01-0.44） | 97289（13817-267396） | 3.93（0.51-11.02） | -0.40(-0.42--0.36) | -0.33(-0.36--0.30) |
| Low-middle SDI | 275（18-919） | 0.08(0.00-0.26) | 4985（3112-7174） | 2.20(0.16-7.11) | 974(42-3396) | 0.10（0.00-0.35） | 29664（1793-101195） | 2.45（0.13-8.42） | 0.81(0.67-0.95) | 0.35(0.32-0.38) |
| **Lead exposure** |  |  |  |  |  |  |  |  |  |  |
| Southeast Asia | 119（-18-324） | 0.08(-0.01-0.22) | 4417（-582-11545） | 2.14(-0.29-5.64) | 483(-72-1159) | 0.11（-0.02-0.27） | 13484（-1739-34002） | 2.52（-0.33-6.30） | 1.17(1.15-1.20) | 0.53(0.52-0.54) |
| Oceania | 1（-0-2） | 0.06(-0.01-0.17) | 29（-4-76） | 1.44(-0.19-3.77) | 3(-0-7) | 0.06（-0.01-0.17） | 79（-11-205） | 1.50（-0.21-3.83） | 0.17(0.14-0.19) | 0.13(0.12-0.15) |
| East Asia | 524（-73-1334） | 0.14(-0.02-0.36) | 17817（-2261-46085） | 2.91(-0.38-7.55) | 2357(-382-5905) | 0.15（-0.02-0.37） | 58933（-7972-151587） | 3.07（-0.43-7.95） | 0.20(0.15-0.24) | 0.17(0.14-0.19) |
| Central Asia | 10（-1-27） | 0.03(-0.00-0.07) | 427（-53-1146） | 1.01(-0.13-2.72) | 26(-4-64) | 0.05（-0.01-0.11） | 913（-114-2393） | 1.34（-0.17-3.47） | 1.58(1.40-1.76) | 0.89(0.80-0.98) |
| Central Europe | 86（-12-218） | 0.07(-0.01-0.19) | 2406（-304-6226） | 1.75(-0.22-4.51) | 192(-28-483) | 0.08（-0.01-0.20） | 4505（-592-11522） | 1.89（-0.25-4.86） | 0.20(0.16-0.22) | 0.24(0.20-0.26) |
| High-income Asia Pacific | 71（-10-181） | 0.04(-0.01-0.11) | 2295（-288-5952） | 1.21(-0.15-3.11) | 271(-41-681) | 0.04（-0.01-0.10） | 5504（-726-14141） | 0.99（-0.13-2.54） | -0.36(-0.44--0.29) | -0.59(-0.63--0.55) |
| Eastern Europe | 74（-11-194） | 0.04(-0.01-0.09) | 2328（-296-6042） | 0.91(-0.12-2.36) | 184(-27-454) | 0.05（-0.01-0.12） | 4533（-574-11577） | 1.25（-0.16-3.19） | 1.28(1.19-1.37) | 1.09(1.05-1.14) |
| Western Europe | 466（-67-1173） | 0.08(-0.01-0.20) | 12372（-1647-31828） | 2.09(-0.28-5.36) | 1272(-190-3177) | 0.10（-0.01-0.24） | 24606（-3332-62513） | 2.17（-0.29-5.60） | 0.57(0.52-0.61) | 0.13(0.11-0.15) |
| Australasia | 35（-5-88） | 0.17(-0.03-0.43) | 887（-119-2313） | 3.94(-0.53-10.18) | 117(-17-296) | 0.18（-0.03-0.45） | 2242（-296-5722） | 3.70（-0.49-9.49） | 0.04(-0.04-0.11) | -0.23(-0.30--0.16) |
| Southern Latin America | 15（-2-39） | 0.04(-0.01-0.11) | 448（-55-1151） | 1.05(-0.13-2.67) | 50(-7-124) | 0.05（-0.01-0.13） | 1029（-136-2585） | 1.13（-0.15-2.84） | 0.82(0.76-0.88) | 0.26(0.22-0.30) |
| High-income North America | 234（-35-574） | 0.06(-0.01-0.16) | 7212（-914-18437） | 1.97(-0.25-5.02) | 594(-89-1517) | 0.08（-0.01-0.20） | 14693（-1960-37550） | 2.05（-0.27-5.23） | 0.65(0.61-0.68) | 0.11(0.10-0.13) |
| Andean Latin America | 18（-3-48） | 0.11(-0.02-0.30) | 482（-63-1263） | 2.67(-0.35-6.99) | 61(-8-156) | 0.11（-0.02-0.29） | 1602（-206-4103） | 2.84（-0.37-7.29） | 0.02(-0.08-0.11) | 0.21(0.14-0.27) |
| Caribbean | 38（-5-96） | 0.19(-0.03-0.48) | 1021（-135-2574） | 4.36(-0.58-10.96) | 107(-16-271) | 0.19（-0.03-0.48） | 2368（-317-5883） | 4.33（-0.58-10.75） | 0.05(0.00-0.10) | -0.01(-0.03-0.02) |
| Central Latin America | 112（-16-277） | 0.20(-0.03-0.49) | 3211（-417-8165） | 4.65(-0.61-11.73) | 419(-63-1052) | 0.18（-0.03-0.46） | 10098（-1358-25197） | 4.29（-0.58-10.69） | -0.23(-0.30--0.17) | -0.26(-0.29--0.23) |
| South Asia | 344（-51-941） | 0.11(-0.02-0.29) | 14358（-1980-36680） | 3.29(-0.47-8.51) | 1797(-295-4572) | 0.19（-0.03-0.48） | 50411（-7101-128759） | 4.25（-0.61-10.80） | 1.87(1.78-1.95) | 0.87(0.83-0.91) |
| Tropical Latin America | 80（-12-197） | 0.15(-0.02-0.36) | 2761（-349-7062） | 3.71(-0.48-9.38) | 355(-53-890) | 0.15（-0.02-0.38） | 8715（-1110-22063） | 3.55（-0.45-8.98） | 0.11(0.04-0.20) | -0.14(-0.17--0.12) |
| North Africa and Middle East | 127（-19-323） | 0.13(-0.02-0.33) | 3522（-481-8851） | 2.78(-0.39-6.90) | 436(-62-1086) | 0.15（-0.02-0.39） | 10153（-1335-25455） | 2.99（-0.40-7.44） | 0.60(0.54-0.65) | 0.25(0.23-0.27) |
| Central Sub-Saharan Africa | 11（-2-31） | 0.10(-0.01-0.28) | 373（-56-989） | 2.32(-0.36-6.22) | 40(-6-107) | 0.14（-0.02-0.39） | 1109（-171-2852） | 2.98（-0.47-7.75） | 1.19(1.17-1.21) | 0.82(0.81-0.84) |
| Eastern Sub-Saharan Africa | 51（-7-139） | 0.13(-0.02-0.36) | 1650（-245-4298） | 3.01(-0.46-7.79) | 119(-18-310) | 0.13（-0.02-0.34） | 3521（-508-8956） | 2.91（-0.43-7.46） | 0.00(-0.03-0.03) | -0.10(-0.12--0.09) |
| Southern Sub-Saharan Africa | 10（-1-27） | 0.06(-0.01-0.15) | 367（-47-960） | 1.59(-0.20-4.17) | 31(-4-76) | 0.09（-0.01-0.21） | 912（-116-2358） | 1.99（-0.26-5.12） | 1.48(1.42-1.54) | 0.74(0.71-0.77) |
| Western Sub-Saharan Africa | 55（-9-150） | 0.11(-0.02-0.31) | 1476（-209-3876） | 2.28(-0.33-5.99) | 142(-22-362) | 0.14（-0.02-0.35） | 3605（-506-9329） | 2.66（-0.38-6.80） | 0.62(0.59-0.65) | 0.50(0.48-0.52) |
| High-middle SDI | 524（-75-1323） | 0.08(-0.01-0.19) | 16439（-2101-42275） | 1.92(-0.25-4.87) | 1954(-303-4956) | 0.11（-0.02-0.27） | 44581（-5926-112242） | 2.30（-0.31-5.80） | 1.07(1.02-1.12) | 0.59(0.56-0.61) |
| High SDI | 738（-106-1835） | 0.07(-0.01-0.17) | 20312（-2620-51855） | 1.84(-0.24-4.67) | 2017(-300-5034) | 0.07（-0.01-0.18） | 43025（-5725-110237） | 1.79（-0.24-4.60） | 0.21(0.18-0.23) | -0.08(-0.09--0.07) |
| Low-middle SDI | 420（-68-1141） | 0.12(-0.02-0.32) | 15659（-2137-39673） | 3.33(-0.46-8.51) | 1800(-282-4662) | 0.19（-0.03-0.49） | 48642（-6668-124412） | 4.23（-0.59-10.87） | 1.51(1.39-1.62) | 0.80(0.74-0.85) |
| Low SDI | 150（-23-411） | 0.13(-0.02-0.35) | 5404（-789-14116） | 3.25(-0.49-8.58) | 522(-83-1375) | 0.19（-0.03-0.49） | 14958（-2194-37534） | 4.10（-0.62-10.39） | 1.31(1.24-1.39) | 0.77(0.72-0.80) |
| Middle SDI | 644（-90-1654） | 0.12(-0.02-0.29) | 21950（-2769-55497） | 2.79(-0.36-7.10) | 2751(-434-6812) | 0.14（-0.02-0.35） | 71606（-9491-180990） | 3.09（-0.42-7.77） | 0.60(0.57-0.62) | 0.32(0.31-0.34) |

Table S4. The burden of AF/AFL attributable to metabolic,behavioral,and environmental risk factors for both sexes combined in 204 countries and territories in 2021.

| **Countries and  territories** | **High systolic blood pressure** | | | |
| --- | --- | --- | --- | --- |
|  | **Deaths (95%UI)** | **ASMR (95%UI)** | **DALYs (95%UI)** | **ASDR (95%UI)** |
| Taiwan (Province of China) | 454（151-775） | 0.96（0.33-1.66） | 10724(3637-18667) | 24.14(8.26-42.01) |
| Cambodia | 78（26-137） | 1.26（0.41-2.19） | 2231(698-4009) | 26.13(8.65-45.66) |
| Lao People's Democratic Republic | 39（14-68） | 1.56（0.54-2.75） | 1129(378-1938) | 33.38(11.44-58.50) |
| Democratic People's Republic of Korea | 342（101-630） | 1.48（0.43-2.75） | 8281(2522-14732) | 29.51(8.64-52.44) |
| Indonesia | 2730（1012-4409） | 2.49（0.92-4.04） | 84029(28825-136990) | 51.28(18.42-82.69) |
| Maldives | 3（1-5） | 1.26（0.40-2.19） | 83(27-140) | 30.05(9.64-49.61) |
| Philippines | 646（214-1144） | 1.26（0.41-2.26） | 18847(6298-33279) | 29.26(9.79-51.52) |
| Malaysia | 346（125-552） | 1.82（0.67-2.89） | 9857(3289-16091) | 41.82(13.95-68.59) |
| Myanmar | 518（183-859） | 1.63（0.57-2.71） | 13707(4654-22858) | 35.15(12.24-58.42) |
| Thailand | 1363（439-2445） | 1.23（0.40-2.19） | 28921(9094-50820) | 26.46(8.31-46.30) |
| Viet Nam | 1407（470-2362） | 2.04（0.69-3.45） | 33002(11256-54144) | 40.99(14.16-67.45) |
| Sri Lanka | 251（90-435） | 1.29（0.46-2.22） | 7445(2645-12692) | 31.20(11.02-53.18) |
| Timor-Leste | 7（3-12） | 1.39（0.52-2.40） | 222(77-371) | 32.39(11.44-54.35) |
| Fiji | 9（3-14） | 2.10（0.70-3.49） | 282(93-469) | 46.99(15.97-77.04) |
| Marshall Islands | 0（0-1） | 1.99（0.69-3.40） | 9(3-17) | 40.04(13.31-67.98) |
| Papua New Guinea | 21（6-39） | 0.71（0.19-1.42） | 804(248-1477) | 19.56(5.81-36.40) |
| Kiribati | 0（0-1） | 1.03（0.34-1.85） | 13(4-23) | 25.52(7.85-44.47) |
| Micronesia (Federated States of) | 1（0-1） | 2.13（0.69-3.87） | 21(6-37) | 40.02(12.40-70.52) |
| Solomon Islands | 2（1-4） | 1.24（0.34-2.70） | 66(21-122) | 27.14(8.04-50.43) |
| Samoa | 2（1-3） | 1.91（0.67-3.32） | 50(16-87) | 41.15(13.90-71.03) |
| Vanuatu | 2（1-3） | 1.81（0.64-3.03） | 60(20-100) | 44.65(15.90-72.77) |
| Tonga | 1（0-2） | 1.51（0.51-2.67） | 28(9-47) | 36.89(12.05-62.16) |
| Azerbaijan | 42（15-70） | 0.62（0.22-1.03） | 1938(643-3398) | 22.82(7.65-38.99) |
| Kazakhstan | 135（48-213） | 1.16（0.42-1.82） | 4799(1715-7877) | 32.04(11.38-52.01) |
| Armenia | 36（13-59） | 0.83（0.30-1.38） | 1112(382-1909) | 25.32(8.71-43.35) |
| Georgia | 120（45-191） | 1.78（0.66-2.83） | 2746(943-4522) | 43.21(14.72-71.07) |
| Kyrgyzstan | 23（9-39） | 0.71（0.27-1.19） | 843(296-1434) | 21.63(7.82-36.22) |
| Tajikistan | 13（5-21） | 0.38（0.13-0.62） | 806(258-1403) | 17.58(5.63-30.28) |
| Mongolia | 9（3-15） | 0.64（0.22-1.08） | 439(149-744) | 23.60(7.88-39.86) |
| Uzbekistan | 72（24-116） | 0.41（0.13-0.66） | 3972(1297-6976) | 17.87(5.87-31.15) |
| Turkmenistan | 29（11-50） | 1.00（0.35-1.71） | 1002(340-1654) | 29.65(10.20-48.65) |
| Albania | 63（21-105） | 1.63（0.52-2.71） | 1551(483-2531) | 36.07(11.32-58.58) |
| Bulgaria | 307（108-498） | 2.07（0.72-3.34） | 6837(2361-11332) | 45.06(15.49-74.18) |
| Bosnia and Herzegovina | 88（30-144） | 1.38（0.48-2.26） | 2222(730-3644) | 34.41(11.36-56.23) |
| China | 19587（6868-32943） | 1.29（0.45-2.22） | 493593(166429-854027) | 26.71(9.10-46.33) |
| Czechia | 372（129-618） | 1.55（0.54-2.57） | 10460(3523-17463) | 44.52(14.99-73.96) |
| North Macedonia | 51（16-92） | 2.50（0.83-4.29） | 1263(425-2086) | 46.30(15.17-77.21) |
| Croatia | 125（43-202） | 1.20（0.41-1.93） | 2617(902-4281) | 26.42(9.14-43.33) |
| Hungary | 310（119-477） | 1.38（0.53-2.13） | 7600(2743-11907) | 35.64(12.75-55.98) |
| Poland | 1130（403-1898） | 1.40（0.51-2.35） | 29244(9863-49782) | 38.24(12.85-65.07) |
| Montenegro | 46（17-77） | 5.84（2.04-9.76） | 810(297-1353) | 91.23(32.87-151.72) |
| Serbia | 290（98-455） | 1.65（0.56-2.61） | 5851(1945-9246) | 33.41(11.17-52.94) |
| Slovenia | 72（26-116） | 1.24（0.44-1.99） | 1600(542-2622) | 31.88(10.80-52.27) |
| Romania | 520（180-814） | 1.22（0.42-1.91） | 11464(3925-18410) | 28.07(9.60-45.14) |
| Slovakia | 193（67-319） | 2.05（0.71-3.39） | 5066(1653-8195) | 51.96(16.90-84.38) |
| Belarus | 237（89-377） | 1.42（0.53-2.26） | 5948(2082-9922) | 36.06(12.65-60.25) |
| Latvia | 78（30-128） | 1.57（0.58-2.55） | 1687(609-2739) | 37.97(13.43-62.14) |
| Republic of Moldova | 79（30-125） | 1.29（0.49-2.03） | 2255(810-3632) | 37.08(13.26-59.98) |
| Ukraine | 1110（355-1805） | 1.40（0.44-2.28） | 27295(9218-44945) | 34.00(11.40-55.83) |
| Estonia | 66（24-108） | 1.85（0.68-3.03） | 1250(424-2015) | 40.11(13.61-64.97) |
| Lithuania | 115（40-188） | 1.59（0.55-2.61） | 2478(861-3988) | 38.58(13.46-62.67) |
| Russian Federation | 3656（1375-5717） | 1.50（0.56-2.34） | 90887(31794-147137) | 37.13(12.94-60.12) |
| Japan | 3992（1350-6696） | 0.69（0.24-1.14） | 80867(27487-137357) | 18.58(6.30-31.37) |
| Singapore | 23（8-39） | 0.28（0.10-0.49） | 903(290-1615) | 10.72(3.46-19.10) |
| Brunei Darussalam | 3（1-6） | 1.76（0.59-3.11） | 92(30-159) | 34.66(11.18-60.07) |
| Republic of Korea | 743（237-1366） | 0.81（0.26-1.50） | 20133(6115-36721) | 21.43(6.53-39.16) |
| New Zealand | 212（75-353） | 2.18（0.77-3.64） | 3988(1245-6931) | 43.61(13.52-75.78) |
| Australia | 989（338-1717） | 1.77（0.61-3.06） | 20626(6616-35404) | 41.03(13.15-70.57) |
| Andorra | 2（1-4） | 1.16（0.39-2.03） | 54(18-93) | 32.46(11.21-56.40) |
| Belgium | 458（162-750） | 1.39（0.49-2.25） | 9277(3170-15251) | 34.15(11.53-56.59) |
| Denmark | 287（103-483） | 2.00（0.72-3.35） | 6275(2190-10572) | 47.75(16.61-81.08) |
| Austria | 519（183-878） | 2.13（0.75-3.60） | 11812(4142-19913) | 55.94(19.55-93.96) |
| France | 3054（1036-5250） | 1.42（0.47-2.42） | 57472(18789-94508) | 33.53(11.00-55.36) |
| Cyprus | 39（13-67） | 2.58（0.90-4.45） | 743(249-1237) | 40.63(14.02-68.48) |
| Greece | 407（131-699） | 1.14（0.37-1.94） | 8375(2570-14558) | 28.47(8.82-49.32) |
| Finland | 252（95-407） | 1.50（0.56-2.42） | 6043(2158-10198) | 41.95(14.68-70.56) |
| Germany | 7278（2490-11845） | 2.74（0.94-4.47） | 136807(45676-225557) | 59.40(19.87-98.75) |
| Ireland | 149（53-254） | 1.70（0.60-2.89） | 3058(1013-5159) | 36.12(11.87-61.02) |
| Italy | 2573（919-4402） | 1.19（0.43-2.02） | 55964(19078-94942) | 31.50(10.53-53.60) |
| Iceland | 15（5-26） | 2.08（0.74-3.45） | 288(99-480) | 43.94(15.09-72.99) |
| Malta | 18（6-31） | 1.52（0.53-2.60） | 369(125-631) | 33.38(11.36-57.34) |
| Israel | 241（83-397） | 1.64（0.56-2.69） | 6479(2076-10678) | 48.62(15.55-80.76) |
| Norway | 242（83-406） | 1.86（0.64-3.10） | 4643(1548-7682) | 40.77(13.74-67.85) |
| Luxembourg | 26（9-43） | 2.01（0.69-3.28） | 509(174-839) | 43.45(14.99-71.38) |
| Netherlands | 782（259-1323） | 1.88（0.62-3.17） | 16100(5259-27020) | 41.19(13.26-69.07) |
| Spain | 1937（604-3317） | 1.31（0.42-2.23） | 41562(13139-70452) | 35.59(11.68-59.73) |
| Switzerland | 217（78-382） | 0.85（0.30-1.46） | 4173(1457-7176) | 19.22(6.73-33.49) |
| Portugal | 320（108-537） | 0.95（0.32-1.59） | 7954(2551-13782) | 27.50(8.85-47.47) |
| Sweden | 909（324-1529） | 3.04（1.08-5.12） | 18068(6171-30718) | 68.85(23.33-117.85) |
| Chile | 376（138-591） | 1.40（0.52-2.21） | 7888(2771-12927) | 29.87(10.50-49.00) |
| United Kingdom | 2582（891-4254） | 1.57（0.54-2.57） | 48752(16273-81963) | 32.92(11.04-55.60) |
| Argentina | 655（232-1082） | 1.10（0.39-1.82） | 12585(4302-20602) | 21.55(7.32-35.26) |
| Uruguay | 89（32-148） | 1.21（0.43-2.00） | 1635(597-2764) | 25.13(8.93-43.00) |
| Canada | 1083（362-1798） | 1.22（0.41-2.03） | 26657(8841-46120) | 33.12(10.86-57.32) |
| Antigua and Barbuda | 1（0-2） | 1.88（0.63-3.16） | 35(12-60) | 38.21(13.19-64.86) |
| United States of America | 10247（3364-17534） | 1.52（0.50-2.59） | 247749(78684-423739) | 39.19(12.36-67.08) |
| Bahamas | 5（2-9） | 1.77（0.62-2.99） | 137(46-231) | 38.96(13.15-65.60) |
| Barbados | 9（3-16） | 1.83（0.63-3.06） | 206(72-347) | 39.33(13.64-66.14) |
| Belize | 3（1-5） | 1.31（0.42-2.17） | 83(27-142) | 32.36(10.52-54.88) |
| Cuba | 287（101-508） | 1.29（0.45-2.26） | 6097(2069-10780) | 28.98(9.74-51.39) |
| Dominican Republic | 130（44-217） | 1.39（0.47-2.33） | 3122(1043-5344) | 32.51(10.83-55.55) |
| Guyana | 7（2-12） | 1.60（0.57-2.74） | 196(68-333) | 36.80(12.65-62.19) |
| Dominica | 2（1-3） | 2.25（0.80-3.87） | 34(12-58) | 43.92(15.67-76.13) |
| Grenada | 2（1-3） | 2.03（0.70-3.40） | 39(14-67) | 41.15(14.33-68.99) |
| Jamaica | 57（19-100） | 1.54（0.52-2.78） | 1171(402-1999) | 35.54(12.08-61.21) |
| Saint Vincent and the Grenadines | 2（1-4） | 1.99（0.67-3.34） | 51(17-86) | 39.18(12.93-65.54) |
| Trinidad and Tobago | 29（11-51） | 1.67（0.59-2.89） | 758(252-1287) | 40.52(13.58-69.28) |
| Haiti | 67（20-122） | 1.64（0.50-3.05） | 2180(690-3762) | 39.34(12.69-67.50) |
| Saint Lucia | 5（2-8） | 2.10（0.72-3.65） | 93(30-162) | 40.45(12.89-69.72) |
| Bolivia (Plurinational State of) | 93（29-169） | 1.50（0.48-2.70） | 2256(712-4108) | 30.54(9.74-54.40) |
| Suriname | 7（2-13） | 1.32（0.42-2.41） | 175(62-300) | 29.84(10.53-51.26) |
| Peru | 357（121-608） | 1.06（0.36-1.80） | 9125(3014-15511) | 27.65(9.16-47.00) |
| Colombia | 808（279-1297） | 1.41（0.48-2.25） | 19508(6406-32354) | 35.03(11.49-58.23) |
| Ecuador | 144（52-249） | 1.06（0.38-1.83） | 3553(1190-6231) | 23.53(8.00-40.75) |
| El Salvador | 121（43-210） | 1.64（0.58-2.83） | 2448(864-4128) | 36.54(12.77-61.19) |
| Costa Rica | 84（30-138） | 1.45（0.52-2.39） | 2038(696-3418) | 36.70(12.52-61.54) |
| Honduras | 93（33-161） | 2.26（0.77-3.92） | 2287(779-3973) | 45.04(15.17-76.50) |
| Guatemala | 93（32-163） | 1.09（0.36-1.89） | 2820(957-4856) | 28.27(9.41-48.40) |
| Nicaragua | 44（15-73） | 1.16（0.40-2.00） | 1345(431-2259) | 30.97(10.11-52.09) |
| Panama | 71（24-120） | 1.50（0.50-2.50） | 1623(534-2778) | 35.83(11.71-61.44) |
| Venezuela (Bolivarian Republic of) | 404（143-685） | 1.55（0.56-2.61） | 10405(3572-17739) | 37.64(13.10-63.84) |
| Paraguay | 91（31-155） | 1.81（0.61-3.06） | 2167(712-3665) | 40.50(13.16-68.06) |
| Brazil | 3390（1176-5663） | 1.46（0.51-2.44） | 88739(29204-150453) | 36.69(12.07-62.41) |
| Algeria | 362（125-625） | 1.93（0.69-3.32） | 7258(2486-12522) | 29.88(10.19-50.83) |
| Bahrain | 5（1-9） | 1.76（0.48-3.34） | 134(44-236) | 29.58(9.43-53.15) |
| Iran (Islamic Republic of) | 538（189-917） | 0.90（0.32-1.55） | 13515(4485-22734) | 20.50(6.81-34.49) |
| Egypt | 293（101-485） | 1.04（0.36-1.73） | 9330(3050-15468) | 22.56(7.47-37.60) |
| Iraq | 224（78-389） | 1.66（0.57-2.89） | 5704(1897-9756) | 32.58(10.97-54.89) |
| Jordan | 31（11-54） | 0.80（0.28-1.43） | 998(319-1749) | 18.78(6.04-32.51) |
| Kuwait | 19（7-32） | 0.97（0.34-1.60） | 452(144-756) | 20.77(6.68-34.58) |
| Lebanon | 64（21-111） | 0.91（0.30-1.59） | 1281(412-2187) | 19.56(6.32-33.43) |
| Libya | 37（11-70） | 1.02（0.32-1.92） | 988(321-1748) | 23.50(7.52-41.69) |
| Morocco | 348（119-585） | 1.48（0.51-2.49） | 8081(2820-13480) | 28.90(9.93-47.79) |
| Palestine | 18（6-31） | 1.36（0.44-2.37） | 401(130-689) | 23.44(7.55-40.83) |
| Oman | 15（5-24） | 1.48（0.50-2.58） | 395(132-662) | 29.12(10.19-48.79) |
| Qatar | 3（1-6） | 1.30（0.29-2.53） | 122(39-221) | 25.31(8.13-46.82) |
| Saudi Arabia | 70（23-117） | 0.99（0.34-1.68） | 2455(771-4285) | 21.40(7.09-36.60) |
| Syrian Arab Republic | 98（33-177） | 1.37（0.44-2.42） | 2473(814-4237) | 25.92(8.54-44.39) |
| Tunisia | 122（36-228） | 1.21（0.35-2.24） | 2494(811-4470) | 21.85(7.10-39.06) |
| United Arab Emirates | 9（3-15） | 1.15（0.41-2.02） | 477(152-840) | 25.70(9.16-43.17) |
| Turkey | 856（306-1458） | 1.13（0.40-1.94） | 16477(5604-27875) | 19.97(6.80-34.07) |
| Mexico | 1513（558-2514） | 1.42（0.52-2.35） | 40808(13404-68748) | 35.13(11.71-58.81) |
| Yemen | 83（28-150） | 1.06（0.36-1.92） | 2138(731-3639) | 21.18(7.29-36.64) |
| Afghanistan | 51（16-94） | 0.92（0.30-1.70） | 1396(475-2475) | 20.26(6.90-35.88) |
| Bangladesh | 1337（419-2350） | 1.38（0.44-2.46） | 33031(10482-57224) | 28.57(9.16-49.61) |
| Bhutan | 6（2-11） | 1.21（0.36-2.22） | 141(45-246) | 26.23(8.22-45.74) |
| India | 8182（2615-14216） | 1.01（0.33-1.74） | 247046(80153-423101) | 25.01(8.16-42.45) |
| Nepal | 117（33-223） | 0.78（0.21-1.48） | 3347(1009-6104) | 17.76(5.19-33.45) |
| Pakistan | 950（328-1720） | 1.36（0.47-2.48） | 28327(9933-48126) | 31.33(11.02-54.01) |
| Angola | 90（29-157） | 1.56（0.49-2.79） | 2632(856-4516) | 32.91(10.57-56.77) |
| Central African Republic | 13（3-25） | 1.32（0.36-2.66） | 419(136-755) | 29.24(9.42-52.96) |
| Congo | 27（9-45） | 1.96（0.65-3.32） | 726(238-1197) | 39.10(12.84-64.15) |
| Democratic Republic of the Congo | 254（70-511） | 1.32（0.36-2.65） | 7146(2096-12814) | 28.00(8.30-50.56) |
| Equatorial Guinea | 5（2-9） | 1.82（0.59-3.13） | 135(46-228) | 37.29(12.59-62.34) |
| Gabon | 14（4-23） | 2.30（0.71-3.97） | 321(98-536) | 42.41(13.02-70.58) |
| Burundi | 21（5-48） | 0.83（0.21-1.87） | 769(235-1451) | 21.38(6.38-41.04) |
| Comoros | 3（1-7） | 1.06（0.30-2.14） | 96(30-172) | 24.21(7.54-44.57) |
| Djibouti | 3（1-6） | 1.23（0.40-2.27） | 116(39-205) | 26.96(9.09-47.62) |
| Eritrea | 14（4-30） | 1.22（0.33-2.63） | 443(136-803) | 25.05(7.76-47.00) |
| Ethiopia | 155（43-317） | 0.54（0.14-1.11） | 5918(1845-10777) | 16.26(5.07-29.99) |
| Kenya | 129（38-235） | 1.07（0.32-1.97） | 4265(1356-7347) | 25.29(7.97-43.11) |
| Madagascar | 82（24-151） | 1.55（0.46-2.91） | 2408(792-4342) | 31.55(9.87-56.28) |
| Malawi | 41（13-74） | 0.97（0.30-1.76） | 1446(449-2493) | 25.36(7.97-43.23) |
| Mauritius | 25（8-42） | 1.62（0.54-2.68） | 614(205-1002) | 36.28(11.95-59.52) |
| Mozambique | 82（26-153） | 1.38（0.42-2.59） | 2533(809-4507) | 30.44(9.98-53.59) |
| Rwanda | 30（7-62） | 0.90（0.20-1.86） | 991(276-1860) | 21.40(6.04-40.22) |
| Seychelles | 1（1-3） | 1.76（0.62-3.00） | 37(13-63) | 37.13(12.87-63.90) |
| Somalia | 17（4-39） | 0.65（0.16-1.46） | 761(222-1437) | 18.30(5.60-34.56) |
| United Republic of Tanzania | 164（44-316） | 1.00（0.27-1.93） | 4931(1513-8717) | 23.77(7.42-42.24) |
| Uganda | 78（20-158） | 0.90（0.22-1.85） | 2582(754-4595) | 22.83(6.54-40.60) |
| Zambia | 57（16-120） | 1.44（0.41-2.92） | 1520(452-2956) | 29.36(8.69-55.55) |
| Botswana | 9（3-15） | 1.04（0.35-1.85） | 284(95-479) | 25.53(8.35-42.98) |
| Lesotho | 7（2-12） | 1.10（0.37-1.92） | 209(68-354) | 25.55(8.57-43.14) |
| Namibia | 9（3-15） | 1.17（0.36-2.01） | 272(87-448) | 26.10(8.37-43.35) |
| South Africa | 416（146-665） | 1.38（0.49-2.24） | 11910(3889-19690) | 31.36(10.35-51.82) |
| Eswatini | 4（1-6） | 1.20（0.39-2.08） | 124(41-205) | 29.73(9.78-50.13) |
| Zimbabwe | 42（14-72） | 1.24（0.42-2.10） | 1448(471-2391) | 29.55(9.84-48.82) |
| Benin | 35（12-60） | 1.19（0.40-2.02） | 916(293-1519) | 24.12(7.89-39.38) |
| Burkina Faso | 87（29-146） | 1.65（0.57-2.81） | 1931(687-3326) | 29.38(10.57-49.49) |
| Cameroon | 115（37-187） | 1.82（0.59-2.95） | 2919(987-4691) | 34.19(11.35-54.50) |
| Cabo Verde | 8（3-13） | 1.86（0.64-3.25） | 134(46-231) | 32.58(11.19-56.43) |
| Chad | 33（11-58） | 1.12（0.36-1.94） | 906(292-1569) | 22.45(7.28-38.94) |
| Côte d'Ivoire | 83（29-134） | 1.58（0.54-2.59） | 2227(735-3681) | 30.29(9.97-49.42) |
| Gambia | 10（4-17） | 1.74（0.60-3.04） | 234(82-388) | 32.08(11.24-53.38) |
| Ghana | 122（43-204） | 1.49（0.53-2.52） | 3335(1115-5595) | 29.37(9.85-48.49) |
| Guinea | 45（15-77） | 1.26（0.42-2.17） | 1058(350-1782) | 24.39(8.16-40.94) |
| Guinea-Bissau | 5（2-8） | 1.68（0.57-2.97） | 135(45-228) | 31.06(10.57-52.93) |
| Liberia | 17（6-30） | 1.54（0.53-2.66） | 418(146-721) | 29.10(10.23-50.01) |
| Mali | 43（14-74） | 1.04（0.35-1.77） | 1267(411-2162) | 21.62(7.32-36.52) |
| Mauritania | 25（8-44） | 1.83（0.59-3.22） | 536(178-912) | 32.46(11.04-54.37) |
| Niger | 36（10-69） | 0.97（0.28-1.81） | 1166(352-2108) | 21.01(6.53-38.11) |
| Nigeria | 745（260-1234） | 1.50（0.52-2.49） | 19322(6447-32128) | 29.92(10.19-49.90) |
| Sao Tome and Principe | 1（0-2） | 1.70（0.60-2.84） | 26(9-42) | 32.00(11.33-52.83) |
| Senegal | 85（31-141） | 1.83（0.67-3.03） | 1885(621-3130) | 32.94(11.26-54.01) |
| Sierra Leone | 31（11-51） | 1.43（0.50-2.31） | 844(297-1375) | 29.95(10.68-48.47) |
| Togo | 28（9-46） | 1.71（0.58-2.81） | 765(243-1290) | 32.01(10.56-52.41) |
| American Samoa | 1（0-1） | 1.98（0.71-3.49） | 18(6-31) | 44.71(15.54-77.25) |
| Bermuda | 2（1-4） | 1.25（0.44-2.16） | 46(15-81) | 30.15(9.84-53.14) |
| Cook Islands | 0（0-1） | 1.82（0.63-3.24） | 10(4-17) | 40.51(14.16-69.12) |
| Greenland | 1（0-1） | 2.04（0.69-3.49） | 26(9-47) | 47.29(16.22-83.73) |
| Guam | 1（0-3） | 0.62（0.21-1.06） | 58(19-100) | 27.02(8.98-46.35) |
| Monaco | 2（1-3） | 1.35（0.46-2.26） | 39(13-66) | 33.92(11.61-57.11) |
| Nauru | 0（0-0） | 3.05（0.93-6.94） | 3(1-5) | 63.84(20.22-115.61) |
| Niue | 0（0-0） | 2.40（0.77-3.97） | 1(0-2) | 49.92(16.39-81.81) |
| Northern Mariana Islands | 1（0-1） | 2.33（0.79-3.90） | 19(7-32) | 47.82(16.34-81.56) |
| Palau | 0（0-0） | 1.43（0.48-2.42） | 6(2-10) | 34.12(11.69-57.34) |
| Puerto Rico | 115（42-195） | 1.15（0.42-1.91） | 2644(891-4495) | 31.45(10.99-53.76) |
| Saint Kitts and Nevis | 1（0-2） | 2.21（0.77-3.70） | 23(8-40) | 43.21(14.96-73.90) |
| San Marino | 1（0-2） | 0.96（0.29-1.78） | 26(8-45) | 29.07(9.22-49.66) |
| Tokelau | 0（0-0） | 2.10（0.67-3.89） | 1(0-1) | 43.82(14.76-76.93) |
| Tuvalu | 0（0-0） | 2.08（0.71-3.53） | 4(1-6) | 43.47(15.57-73.43) |
| United States Virgin Islands | 2（1-4） | 1.40（0.48-2.53） | 58(19-101) | 32.14(10.77-56.10) |
| South Sudan | 18（5-36） | 0.87（0.23-1.74） | 620(194-1144) | 21.65(6.87-40.17) |
| Sudan | 141（50-240） | 1.15（0.40-1.94） | 3865(1326-6537) | 25.76(8.70-43.71) |
| **Countries and  territories** | **High body-mass index** | | | |
|  | **Deaths (95%UI)** | **ASMR (95%UI)** | **DALYs (95%UI)** | **ASDR (95%UI)** |
| Democratic People's Republic of Korea | 57(16-135) | 0.26(0.06-0.62) | 1090(350-2340) | 4.20(1.22-9.43) |
| China | 2378(942-4212) | 0.15(0.06-0.27) | 75252(29280-129907) | 3.84(1.51-6.57) |
| Taiwan (Province of China) | 109(43-198) | 0.23(0.09-0.42) | 2617(1021-4470) | 5.91(2.31-10.04) |
| Cambodia | 2(0-5) | 0.02(0.00-0.06) | 115(35-215) | 0.91(0.25-1.86) |
| Lao People's Democratic Republic | 2(1-5) | 0.07(0.02-0.15) | 95(34-183) | 2.22(0.74-4.24) |
| Indonesia | 106(33-203) | 0.07(0.02-0.15) | 5364(1974-9863) | 2.32(0.77-4.42) |
| Malaysia | 56(22-96) | 0.28(0.11-0.49) | 1916(805-3134) | 7.53(3.09-12.44) |
| Maldives | 0(0-0) | 0.04(0.01-0.10) | 10(4-19) | 2.79(1.04-5.03) |
| Philippines | 80(32-151) | 0.14(0.05-0.28) | 3167(1199-5548) | 4.28(1.59-7.66) |
| Myanmar | 14(4-32) | 0.03(0.01-0.09) | 751(251-1430) | 1.50(0.48-2.97) |
| Thailand | 184(55-387) | 0.17(0.05-0.35) | 5402(2156-9724) | 4.92(1.96-8.90) |
| Sri Lanka | 33(11-71) | 0.16(0.05-0.36) | 1130(426-2121) | 4.53(1.67-8.83) |
| Viet Nam | 25(4-65) | 0.03(0.01-0.10) | 873(246-1790) | 0.94(0.25-2.06) |
| Timor-Leste | 0(0-0) | 0.00(0.00-0.01) | 2(0-5) | 0.25(0.00-0.59) |
| Kiribati | 0(0-0) | 0.37(0.15-0.73) | 9(4-16) | 13.20(5.43-23.16) |
| Fiji | 5(2-8) | 1.13(0.48-1.96) | 165(76-273) | 26.44(11.98-44.22) |
| Micronesia (Federated States of) | 0(0-1) | 0.96(0.38-1.79) | 18(7-30) | 25.20(10.19-43.20) |
| Samoa | 1(0-2) | 0.87(0.33-1.70) | 34(15-58) | 24.70(10.40-42.89) |
| Marshall Islands | 0(0-0) | 0.94(0.38-1.78) | 8(3-14) | 23.95(10.22-43.05) |
| Papua New Guinea | 3(1-7) | 0.06(0.02-0.12) | 187(70-370) | 2.90(1.07-5.64) |
| Tonga | 1(0-1) | 0.81(0.33-1.45) | 19(8-32) | 24.35(10.45-41.44) |
| Armenia | 12(5-22) | 0.29(0.12-0.51) | 424(176-759) | 9.66(3.97-17.33) |
| Solomon Islands | 1(0-1) | 0.17(0.05-0.38) | 25(10-49) | 6.73(2.42-13.48) |
| Vanuatu | 0(0-1) | 0.24(0.09-0.49) | 15(6-28) | 8.61(3.35-16.03) |
| Georgia | 34(15-59) | 0.51(0.22-0.89) | 839(356-1428) | 13.32(5.63-22.84) |
| Azerbaijan | 15(6-26) | 0.21(0.08-0.37) | 788(312-1454) | 8.60(3.37-15.50) |
| Kyrgyzstan | 9(4-15) | 0.26(0.10-0.43) | 385(153-671) | 9.00(3.60-15.50) |
| Kazakhstan | 51(23-86) | 0.43(0.19-0.74) | 1852(775-3282) | 12.14(5.13-21.28) |
| Mongolia | 2(1-4) | 0.14(0.06-0.25) | 109(44-192) | 5.53(2.23-9.56) |
| Turkmenistan | 8(3-15) | 0.26(0.10-0.48) | 301(116-561) | 8.33(3.16-15.23) |
| Tajikistan | 4(2-8) | 0.12(0.05-0.21) | 280(109-513) | 5.76(2.34-10.21) |
| Bosnia and Herzegovina | 31(13-53) | 0.48(0.21-0.83) | 788(333-1358) | 12.25(5.16-21.05) |
| Uzbekistan | 27(11-50) | 0.15(0.06-0.28) | 1567(604-2924) | 6.87(2.63-12.59) |
| Croatia | 49(21-84) | 0.48(0.20-0.82) | 1068(454-1867) | 11.00(4.62-19.29) |
| Albania | 22(8-42) | 0.56(0.22-1.10) | 525(203-1043) | 12.20(4.70-24.34) |
| Hungary | 117(52-202) | 0.53(0.24-0.91) | 3150(1329-5492) | 15.09(6.45-26.11) |
| Bulgaria | 119(51-211) | 0.82(0.35-1.44) | 2729(1142-4882) | 18.25(7.66-32.61) |
| Czechia | 151(61-271) | 0.63(0.26-1.13) | 4585(1865-8303) | 19.72(8.02-35.59) |
| Montenegro | 21(9-38) | 2.72(1.14-4.89) | 395(172-695) | 44.18(18.82-77.58) |
| Romania | 187(79-319) | 0.44(0.19-0.75) | 4313(1762-7403) | 10.75(4.39-18.43) |
| North Macedonia | 20(8-38) | 0.96(0.42-1.79) | 509(205-939) | 18.40(7.59-33.88) |
| Poland | 500(211-899) | 0.62(0.26-1.11) | 12557(5127-22383) | 16.28(6.64-29.05) |
| Serbia | 110(46-196) | 0.62(0.26-1.12) | 2339(974-4143) | 13.40(5.62-23.79) |
| Slovenia | 31(13-53) | 0.53(0.23-0.91) | 701(290-1218) | 14.12(5.83-24.61) |
| Belarus | 91(40-165) | 0.55(0.24-0.99) | 2409(1008-4180) | 14.62(6.11-25.45) |
| Slovakia | 84(38-145) | 0.89(0.40-1.54) | 2330(1022-4081) | 23.84(10.51-41.55) |
| Latvia | 31(13-55) | 0.62(0.26-1.11) | 683(282-1196) | 15.62(6.49-27.61) |
| Republic of Moldova | 34(15-59) | 0.56(0.24-0.95) | 1008(432-1726) | 16.60(7.12-28.50) |
| Ukraine | 469(194-813) | 0.59(0.25-1.03) | 12019(5073-20789) | 15.05(6.35-26.14) |
| Estonia | 25(10-45) | 0.70(0.29-1.26) | 495(204-899) | 16.33(6.87-29.65) |
| Lithuania | 47(20-85) | 0.66(0.28-1.16) | 1034(434-1846) | 16.13(6.69-28.91) |
| Japan | 246(84-508) | 0.04(0.02-0.09) | 5415(1909-10251) | 1.39(0.51-2.62) |
| Singapore | 4(1-8) | 0.05(0.02-0.09) | 250(98-446) | 2.87(1.12-5.08) |
| Russian Federation | 1586(672-2740) | 0.65(0.28-1.12) | 41695(17494-72255) | 17.03(7.16-29.53) |
| Brunei Darussalam | 1(0-1) | 0.15(0.06-0.26) | 25(10-43) | 6.22(2.48-10.46) |
| Republic of Korea | 91(29-198) | 0.10(0.03-0.22) | 2834(1071-5417) | 3.04(1.16-5.84) |
| New Zealand | 80(33-147) | 0.84(0.35-1.53) | 1750(719-3115) | 19.61(8.15-35.06) |
| Australia | 483(197-897) | 0.87(0.35-1.61) | 10664(4621-18755) | 21.73(9.39-38.12) |
| Andorra | 1(0-1) | 0.29(0.11-0.59) | 14(5-28) | 8.74(3.21-17.02) |
| Belgium | 132(52-234) | 0.40(0.16-0.71) | 2684(1080-4624) | 10.03(4.05-17.44) |
| Denmark | 68(26-125) | 0.48(0.18-0.87) | 1564(594-2783) | 12.25(4.70-21.71) |
| France | 907(371-1584) | 0.43(0.18-0.75) | 17684(6785-30575) | 10.61(4.08-18.58) |
| Austria | 150(64-291) | 0.62(0.26-1.19) | 3404(1392-6439) | 16.34(6.57-30.83) |
| Cyprus | 11(4-19) | 0.72(0.29-1.33) | 210(84-384) | 11.66(4.78-21.20) |
| Finland | 83(34-149) | 0.49(0.21-0.88) | 1967(782-3511) | 13.77(5.52-25.10) |
| Greece | 147(57-287) | 0.42(0.16-0.82) | 3204(1267-6114) | 11.56(4.54-21.80) |
| Ireland | 47(19-83) | 0.54(0.22-0.95) | 1032(409-1857) | 12.38(4.91-22.30) |
| Germany | 2071(864-3980) | 0.80(0.33-1.53) | 41697(16784-76351) | 19.02(7.63-34.65) |
| Iceland | 5(2-10) | 0.72(0.28-1.35) | 104(41-192) | 16.19(6.43-30.32) |
| Israel | 74(32-134) | 0.50(0.22-0.91) | 2133(907-3791) | 16.18(6.91-28.94) |
| Luxembourg | 10(4-17) | 0.73(0.28-1.26) | 182(72-317) | 15.51(6.19-26.92) |
| Portugal | 113(47-207) | 0.33(0.14-0.62) | 2734(1035-5042) | 9.49(3.65-17.65) |
| Italy | 888(360-1681) | 0.41(0.17-0.77) | 19002(7515-35242) | 10.76(4.26-19.93) |
| Netherlands | 203(78-374) | 0.49(0.19-0.90) | 4268(1614-7622) | 11.10(4.22-20.13) |
| Malta | 5(2-10) | 0.45(0.19-0.83) | 114(47-205) | 10.47(4.34-18.92) |
| Norway | 52(22-89) | 0.40(0.17-0.68) | 1062(420-1807) | 9.51(3.73-16.16) |
| Argentina | 254(103-475) | 0.43(0.17-0.80) | 5542(2208-10136) | 9.60(3.83-17.48) |
| United Kingdom | 1053(418-1955) | 0.65(0.26-1.20) | 21278(8803-39200) | 14.77(6.11-27.19) |
| Sweden | 227(90-423) | 0.76(0.31-1.41) | 4882(1906-9042) | 19.49(7.62-36.40) |
| Spain | 830(328-1526) | 0.56(0.23-1.04) | 18188(7321-32506) | 15.88(6.49-28.41) |
| Switzerland | 62(25-113) | 0.24(0.10-0.43) | 1197(471-2096) | 5.58(2.18-9.75) |
| Chile | 133(53-238) | 0.50(0.20-0.89) | 3255(1342-5785) | 12.42(5.12-22.08) |
| Canada | 453(182-822) | 0.52(0.21-0.94) | 12932(5263-23457) | 16.63(6.86-30.37) |
| Uruguay | 30(13-56) | 0.42(0.17-0.77) | 618(240-1081) | 9.97(3.94-17.55) |
| United States of America | 5278(2228-9082) | 0.79(0.34-1.36) | 152716(64970-258787) | 24.74(10.59-41.79) |
| Bahamas | 2(1-4) | 0.67(0.26-1.29) | 64(26-116) | 17.04(6.75-31.29) |
| Belize | 2(1-3) | 0.65(0.27-1.14) | 47(21-81) | 17.36(7.57-29.90) |
| Antigua and Barbuda | 0(0-1) | 0.58(0.23-1.10) | 13(5-23) | 13.34(5.18-24.36) |
| Dominica | 1(0-1) | 1.17(0.52-2.05) | 19(8-34) | 24.69(10.88-42.85) |
| Grenada | 0(0-1) | 0.48(0.18-0.91) | 12(5-21) | 11.74(4.73-20.96) |
| Barbados | 3(1-6) | 0.64(0.27-1.22) | 83(34-151) | 15.98(6.45-28.97) |
| Haiti | 5(2-10) | 0.09(0.03-0.21) | 193(79-360) | 2.90(1.12-5.39) |
| Cuba | 90(38-156) | 0.41(0.17-0.70) | 2249(902-3779) | 10.94(4.43-18.41) |
| Dominican Republic | 22(8-43) | 0.23(0.08-0.45) | 795(301-1425) | 8.04(3.04-14.45) |
| Guyana | 2(1-3) | 0.39(0.16-0.71) | 62(25-111) | 10.60(4.28-18.83) |
| Saint Lucia | 1(0-2) | 0.44(0.16-0.83) | 25(10-45) | 10.62(4.24-18.87) |
| Jamaica | 20(9-36) | 0.55(0.25-0.99) | 479(209-820) | 14.66(6.38-25.04) |
| Saint Vincent and the Grenadines | 0(0-1) | 0.35(0.13-0.65) | 11(4-20) | 8.40(3.38-14.90) |
| Mexico | 642(279-1139) | 0.59(0.26-1.05) | 19766(8322-35974) | 16.54(6.95-29.97) |
| Suriname | 1(0-3) | 0.24(0.08-0.48) | 44(16-82) | 7.12(2.48-13.26) |
| Trinidad and Tobago | 10(4-17) | 0.55(0.24-0.99) | 283(118-506) | 14.89(6.26-26.54) |
| Bolivia (Plurinational State of) | 26(10-48) | 0.38(0.15-0.69) | 860(346-1512) | 10.25(4.13-18.21) |
| Ecuador | 65(25-123) | 0.46(0.18-0.88) | 2078(847-3642) | 13.27(5.36-23.26) |
| Peru | 91(36-167) | 0.27(0.11-0.50) | 3035(1251-5432) | 9.10(3.74-16.24) |
| Colombia | 250(109-459) | 0.43(0.19-0.79) | 6576(2605-11806) | 11.76(4.67-21.09) |
| Costa Rica | 27(11-49) | 0.46(0.19-0.84) | 738(292-1308) | 13.23(5.25-23.50) |
| El Salvador | 50(21-93) | 0.67(0.28-1.26) | 1096(473-1978) | 16.58(7.20-29.86) |
| Guatemala | 35(15-65) | 0.42(0.18-0.77) | 1160(487-2095) | 11.46(4.81-20.61) |
| Honduras | 27(11-52) | 0.64(0.25-1.21) | 784(306-1433) | 14.43(5.80-26.84) |
| Nicaragua | 17(7-31) | 0.44(0.17-0.83) | 598(244-1067) | 13.36(5.41-23.98) |
| Venezuela (Bolivarian Republic of) | 159(66-304) | 0.60(0.25-1.15) | 4686(2004-8452) | 16.40(6.93-29.51) |
| Panama | 36(15-63) | 0.76(0.32-1.31) | 864(363-1510) | 19.04(7.99-33.31) |
| Paraguay | 27(10-56) | 0.53(0.20-1.09) | 788(313-1476) | 14.27(5.63-26.86) |
| Brazil | 1238(497-2151) | 0.53(0.21-0.92) | 35706(14339-63076) | 14.60(5.86-25.86) |
| Bahrain | 3(1-5) | 0.96(0.30-1.94) | 89(37-162) | 16.90(6.33-32.41) |
| Algeria | 144(56-264) | 0.72(0.29-1.30) | 3222(1326-5946) | 12.19(5.16-22.43) |
| Iran (Islamic Republic of) | 274(116-485) | 0.46(0.19-0.81) | 7061(2942-12081) | 10.54(4.38-18.06) |
| Egypt | 209(93-351) | 0.73(0.32-1.24) | 6842(3074-11425) | 16.07(7.06-26.75) |
| Iraq | 99(41-183) | 0.73(0.30-1.37) | 2602(1105-4655) | 14.47(6.11-26.28) |
| Jordan | 22(10-39) | 0.56(0.24-1.01) | 766(345-1307) | 13.62(6.10-23.16) |
| Kuwait | 13(6-23) | 0.65(0.29-1.14) | 368(172-625) | 15.45(7.00-25.78) |
| Lebanon | 37(15-68) | 0.53(0.22-0.97) | 743(301-1357) | 11.43(4.62-20.81) |
| Libya | 21(8-40) | 0.57(0.21-1.09) | 600(235-1088) | 13.72(5.32-25.06) |
| Morocco | 89(35-168) | 0.36(0.14-0.69) | 2372(966-4355) | 7.94(3.19-14.64) |
| Palestine | 12(5-21) | 0.94(0.39-1.60) | 289(121-496) | 16.35(6.77-27.68) |
| Qatar | 2(1-4) | 0.88(0.22-1.81) | 99(44-171) | 17.43(6.71-32.18) |
| Oman | 7(3-13) | 0.71(0.30-1.29) | 231(96-399) | 15.13(6.39-26.18) |
| Saudi Arabia | 55(24-95) | 0.70(0.30-1.22) | 2120(971-3574) | 15.59(6.84-26.63) |
| Syrian Arab Republic | 65(26-116) | 0.87(0.35-1.60) | 1734(741-3027) | 17.30(7.27-30.38) |
| Tunisia | 56(21-105) | 0.54(0.20-1.06) | 1195(479-2093) | 10.29(4.10-18.52) |
| Turkey | 459(191-800) | 0.60(0.25-1.05) | 9315(4013-16305) | 11.07(4.71-19.19) |
| United Arab Emirates | 7(3-12) | 0.77(0.31-1.39) | 437(200-739) | 17.91(8.18-30.72) |
| Yemen | 24(9-45) | 0.29(0.10-0.57) | 678(269-1172) | 6.24(2.50-11.30) |
| Bangladesh | 36(8-92) | 0.03(0.01-0.09) | 1386(462-2903) | 1.06(0.33-2.31) |
| Afghanistan | 12(4-23) | 0.19(0.06-0.37) | 413(161-739) | 5.14(1.98-9.27) |
| Bhutan | 0(0-1) | 0.09(0.03-0.19) | 19(7-35) | 3.25(1.17-5.82) |
| India | 675(284-1274) | 0.08(0.03-0.16) | 24727(9521-43934) | 2.32(0.88-4.19) |
| Pakistan | 107(42-205) | 0.13(0.05-0.27) | 4137(1556-6994) | 3.90(1.48-6.89) |
| Nepal | 4(1-9) | 0.02(0.00-0.05) | 219(72-429) | 0.94(0.29-1.78) |
| Angola | 8(3-17) | 0.11(0.03-0.24) | 326(123-610) | 3.09(1.13-5.88) |
| Congo | 5(2-10) | 0.30(0.10-0.60) | 169(70-298) | 7.33(2.81-13.57) |
| Central African Republic | 1(0-3) | 0.09(0.03-0.21) | 55(20-112) | 2.71(0.97-5.76) |
| Democratic Republic of the Congo | 47(15-99) | 0.22(0.07-0.47) | 1510(530-2879) | 5.27(1.87-10.04) |
| Equatorial Guinea | 1(1-3) | 0.47(0.18-0.87) | 42(16-73) | 10.60(3.89-18.41) |
| Gabon | 4(2-8) | 0.64(0.26-1.23) | 117(48-211) | 13.68(5.70-24.17) |
| Burundi | 1(0-2) | 0.02(0.00-0.06) | 30(8-64) | 0.69(0.18-1.52) |
| Comoros | 0(0-1) | 0.12(0.04-0.25) | 16(6-29) | 3.59(1.36-6.63) |
| Djibouti | 0(0-0) | 0.02(0.00-0.07) | 5(1-10) | 0.78(0.17-1.72) |
| Eritrea | 1(0-1) | 0.04(0.01-0.09) | 28(10-53) | 1.13(0.38-2.27) |
| Ethiopia | 7(3-15) | 0.02(0.01-0.05) | 388(143-746) | 0.92(0.33-1.76) |
| Kenya | 14(6-26) | 0.10(0.04-0.18) | 697(289-1203) | 3.28(1.30-5.66) |
| Madagascar | 8(3-17) | 0.14(0.04-0.31) | 284(106-540) | 3.27(1.22-6.11) |
| Malawi | 4(1-8) | 0.09(0.03-0.20) | 140(52-260) | 2.38(0.88-4.52) |
| Mauritius | 5(2-8) | 0.30(0.12-0.53) | 138(56-237) | 7.84(3.17-13.41) |
| Mozambique | 6(2-12) | 0.07(0.02-0.16) | 273(91-514) | 2.45(0.80-4.73) |
| Rwanda | 1(0-3) | 0.03(0.00-0.08) | 66(20-133) | 1.12(0.32-2.39) |
| Seychelles | 0(0-1) | 0.46(0.17-0.84) | 13(5-22) | 11.90(4.78-20.42) |
| Somalia | 1(0-3) | 0.02(0.00-0.06) | 74(25-145) | 1.13(0.35-2.33) |
| United Republic of Tanzania | 33(11-64) | 0.19(0.07-0.38) | 1156(470-2052) | 5.26(2.14-9.39) |
| Uganda | 4(1-9) | 0.04(0.01-0.09) | 221(81-427) | 1.53(0.53-3.13) |
| Zambia | 9(3-21) | 0.19(0.06-0.43) | 331(125-658) | 5.26(1.95-10.16) |
| Botswana | 3(1-6) | 0.41(0.16-0.74) | 111(45-189) | 9.87(3.93-16.74) |
| Lesotho | 3(1-5) | 0.43(0.17-0.77) | 91(40-161) | 10.49(4.67-18.52) |
| Namibia | 4(2-7) | 0.50(0.21-0.90) | 114(46-195) | 10.97(4.46-19.03) |
| South Africa | 184(84-308) | 0.60(0.27-1.00) | 5810(2482-9939) | 14.72(6.33-25.11) |
| Eswatini | 2(1-4) | 0.79(0.35-1.31) | 75(33-124) | 18.71(8.09-30.59) |
| Zimbabwe | 10(4-19) | 0.24(0.09-0.44) | 414(172-715) | 6.93(2.76-12.24) |
| Benin | 4(1-8) | 0.12(0.04-0.25) | 167(65-295) | 3.66(1.42-6.50) |
| Burkina Faso | 1(0-4) | 0.02(0.00-0.06) | 50(11-119) | 0.57(0.10-1.44) |
| Cameroon | 34(14-60) | 0.53(0.20-0.95) | 973(413-1779) | 10.62(4.45-18.59) |
| Cabo Verde | 1(0-3) | 0.33(0.12-0.63) | 27(11-48) | 6.52(2.63-11.29) |
| Chad | 3(1-6) | 0.09(0.03-0.21) | 104(38-195) | 2.26(0.75-4.45) |
| Côte d'Ivoire | 13(5-24) | 0.24(0.09-0.45) | 432(174-742) | 5.20(2.19-9.01) |
| Gambia | 1(0-2) | 0.20(0.07-0.39) | 38(15-65) | 4.62(1.76-8.15) |
| Ghana | 23(9-43) | 0.28(0.10-0.55) | 745(288-1268) | 6.04(2.22-10.96) |
| Guinea | 5(1-10) | 0.12(0.04-0.26) | 138(52-254) | 2.87(1.04-5.38) |
| Guinea-Bissau | 1(0-1) | 0.15(0.05-0.30) | 19(8-33) | 3.48(1.41-6.17) |
| Liberia | 3(1-6) | 0.26(0.09-0.51) | 115(47-204) | 6.57(2.55-11.76) |
| Mali | 3(1-5) | 0.05(0.01-0.11) | 118(42-214) | 1.55(0.53-2.74) |
| Mauritania | 10(4-17) | 0.71(0.27-1.26) | 205(81-351) | 12.45(4.77-21.59) |
| Niger | 2(1-5) | 0.05(0.01-0.12) | 106(38-209) | 1.49(0.49-3.05) |
| Nigeria | 183(76-319) | 0.38(0.16-0.65) | 4651(1867-7982) | 7.25(2.93-12.45) |
| Sao Tome and Principe | 0(0-0) | 0.35(0.13-0.67) | 6(3-11) | 7.17(2.92-13.00) |
| Senegal | 9(4-17) | 0.19(0.07-0.35) | 277(108-490) | 4.27(1.71-7.54) |
| Sierra Leone | 2(1-5) | 0.10(0.03-0.20) | 88(34-158) | 2.71(1.05-4.98) |
| Togo | 4(2-8) | 0.23(0.09-0.47) | 134(51-237) | 4.95(1.88-8.93) |
| American Samoa | 0(0-1) | 1.03(0.42-1.83) | 13(6-21) | 28.01(12.58-47.89) |
| Bermuda | 1(0-2) | 0.58(0.23-1.09) | 24(10-43) | 16.02(6.63-29.42) |
| Cook Islands | 0(0-0) | 0.85(0.34-1.52) | 6(3-11) | 23.84(10.06-41.88) |
| Greenland | 0(0-1) | 0.83(0.33-1.47) | 13(6-24) | 22.01(9.17-39.28) |
| Guam | 1(0-1) | 0.25(0.10-0.46) | 26(11-47) | 12.15(5.06-22.07) |
| Monaco | 1(0-1) | 0.49(0.20-0.90) | 15(6-27) | 13.05(5.06-24.52) |
| Nauru | 0(0-0) | 1.34(0.50-2.85) | 2(1-3) | 34.50(14.40-61.45) |
| Niue | 0(0-0) | 0.93(0.37-1.69) | 0(0-1) | 23.93(10.10-42.38) |
| Northern Mariana Islands | 0(0-1) | 1.12(0.48-1.96) | 13(6-21) | 27.47(12.16-46.85) |
| Palau | 0(0-0) | 0.66(0.27-1.23) | 4(2-7) | 18.86(8.09-33.37) |
| Puerto Rico | 51(22-94) | 0.52(0.22-0.94) | 1314(561-2319) | 16.57(7.10-29.04) |
| Saint Kitts and Nevis | 0(0-1) | 0.75(0.30-1.35) | 9(4-17) | 16.23(6.45-29.25) |
| San Marino | 0(0-1) | 0.33(0.12-0.64) | 9(3-17) | 10.56(3.97-19.18) |
| Tokelau | 0(0-0) | 0.86(0.35-1.64) | 0(0-1) | 22.41(9.52-40.33) |
| Tuvalu | 0(0-0) | 0.74(0.30-1.47) | 2(1-4) | 21.12(9.04-37.35) |
| United States Virgin Islands | 1(0-2) | 0.71(0.29-1.30) | 31(13-55) | 17.27(7.14-30.81) |
| South Sudan | 0(0-1) | 0.01(0.00-0.03) | 15(2-37) | 0.37(0.04-0.91) |
| Sudan | 49(18-86) | 0.38(0.14-0.68) | 1499(594-2601) | 9.32(3.59-15.90) |
| **Countries and  territories** | **Alcohol use** | | | |
|  | **Deaths (95%UI)** | **ASMR (95%UI)** | **DALYs (95%UI)** | **ASDR (95%UI)** |
| Taiwan (Province of China) | 31(20-42) | 0.07(0.04-0.09) | 1069(719-1462) | 2.47(1.67-3.39) |
| China | 1528(1063-2166) | 0.09(0.06-0.13) | 61537(41531-84129) | 3.01(2.05-4.10) |
| Cambodia | 7(5-11) | 0.10(0.06-0.15) | 341(221-471) | 3.08(2.04-4.20) |
| Democratic People's Republic of Korea | 14(10-20) | 0.05(0.03-0.07) | 638(417-907) | 1.96(1.31-2.76) |
| Lao People's Democratic Republic | 4(2-6) | 0.13(0.07-0.21) | 169(106-247) | 4.08(2.54-5.99) |
| Maldives | 0(0-0) | 0.01(0.00-0.02) | 1(0-2) | 0.31(0.09-0.69) |
| Indonesia | 7(2-13) | 0.00(0.00-0.01) | 393(119-740) | 0.17(0.05-0.33) |
| Malaysia | 4(2-7) | 0.02(0.01-0.03) | 183(96-300) | 0.68(0.36-1.13) |
| Philippines | 72(51-97) | 0.13(0.09-0.18) | 3082(2101-4192) | 4.10(2.77-5.59) |
| Thailand | 95(66-136) | 0.09(0.06-0.12) | 2972(2082-3959) | 2.72(1.91-3.63) |
| Myanmar | 23(14-36) | 0.07(0.04-0.10) | 856(574-1205) | 1.90(1.28-2.67) |
| Viet Nam | 135(85-204) | 0.18(0.11-0.28) | 4512(3065-6539) | 4.98(3.36-7.14) |
| Sri Lanka | 9(5-14) | 0.04(0.03-0.07) | 427(280-585) | 1.65(1.08-2.29) |
| Timor-Leste | 0(0-1) | 0.06(0.04-0.10) | 15(9-22) | 1.94(1.15-2.81) |
| Kiribati | 0(0-0) | 0.01(0.00-0.02) | 0(0-1) | 0.28(0.03-0.75) |
| Micronesia (Federated States of) | 0(0-0) | 0.05(0.02-0.09) | 1(1-2) | 1.54(0.80-2.52) |
| Fiji | 0(0-0) | 0.05(0.03-0.08) | 13(8-19) | 1.75(1.08-2.50) |
| Marshall Islands | 0(0-0) | 0.06(0.02-0.11) | 1(0-1) | 1.72(0.85-2.78) |
| Samoa | 0(0-0) | 0.04(0.02-0.07) | 2(1-3) | 1.37(0.62-2.21) |
| Papua New Guinea | 1(0-1) | 0.02(0.01-0.04) | 35(18-56) | 0.69(0.35-1.11) |
| Tonga | 0(0-0) | 0.02(0.00-0.03) | 0(0-1) | 0.53(0.16-1.06) |
| Solomon Islands | 0(0-0) | 0.01(0.00-0.03) | 2(1-3) | 0.47(0.17-0.84) |
| Armenia | 2(1-3) | 0.04(0.02-0.06) | 87(50-125) | 1.99(1.16-2.87) |
| Vanuatu | 0(0-0) | 0.04(0.02-0.07) | 3(1-4) | 1.52(0.79-2.28) |
| Georgia | 11(6-17) | 0.18(0.10-0.27) | 323(197-471) | 5.36(3.35-7.78) |
| Azerbaijan | 3(2-5) | 0.04(0.02-0.06) | 236(139-348) | 2.33(1.33-3.43) |
| Kazakhstan | 9(5-14) | 0.06(0.04-0.11) | 519(327-747) | 2.95(1.81-4.27) |
| Mongolia | 1(1-1) | 0.05(0.03-0.09) | 66(41-95) | 2.90(1.76-4.24) |
| Kyrgyzstan | 1(1-2) | 0.03(0.02-0.04) | 82(51-115) | 1.63(0.96-2.32) |
| Turkmenistan | 2(1-3) | 0.06(0.03-0.08) | 105(64-153) | 2.64(1.58-3.86) |
| Tajikistan | 0(0-0) | 0.00(0.00-0.00) | 34(20-52) | 0.47(0.28-0.72) |
| Uzbekistan | 4(3-6) | 0.02(0.01-0.03) | 391(239-577) | 1.45(0.85-2.16) |
| Albania | 5(3-7) | 0.11(0.06-0.18) | 164(105-233) | 3.74(2.40-5.33) |
| Bosnia and Herzegovina | 10(6-14) | 0.15(0.09-0.22) | 310(208-414) | 4.91(3.31-6.57) |
| Croatia | 17(11-24) | 0.17(0.11-0.24) | 472(308-648) | 5.01(3.33-6.82) |
| Hungary | 40(26-53) | 0.18(0.12-0.24) | 1267(867-1699) | 6.28(4.29-8.34) |
| Bulgaria | 62(43-82) | 0.42(0.28-0.55) | 1584(1143-2085) | 10.78(7.83-14.15) |
| Czechia | 81(56-106) | 0.34(0.23-0.44) | 2806(1965-3613) | 12.29(8.60-15.93) |
| Montenegro | 7(4-11) | 0.87(0.52-1.32) | 143(99-196) | 15.55(10.55-21.58) |
| North Macedonia | 6(4-10) | 0.27(0.15-0.39) | 211(135-300) | 6.81(4.40-9.47) |
| Romania | 75(49-103) | 0.18(0.12-0.25) | 2087(1423-2839) | 5.38(3.66-7.29) |
| Poland | 173(115-231) | 0.22(0.15-0.29) | 6087(4107-8175) | 8.29(5.61-11.10) |
| Serbia | 35(22-48) | 0.20(0.13-0.28) | 876(586-1158) | 5.14(3.46-6.75) |
| Slovenia | 6(2-12) | 0.12(0.04-0.21) | 193(76-331) | 4.13(1.64-7.05) |
| Belarus | 23(12-36) | 0.14(0.07-0.21) | 892(515-1292) | 5.51(3.21-7.94) |
| Slovakia | 31(20-42) | 0.32(0.21-0.44) | 1013(688-1325) | 10.45(7.07-13.67) |
| Latvia | 10(5-16) | 0.21(0.11-0.32) | 300(177-429) | 7.57(4.70-10.69) |
| Republic of Moldova | 10(5-16) | 0.16(0.09-0.25) | 385(233-551) | 6.43(3.96-9.25) |
| Ukraine | 80(37-134) | 0.10(0.05-0.17) | 3074(1758-4536) | 3.96(2.29-5.82) |
| Estonia | 7(3-11) | 0.20(0.10-0.33) | 179(99-271) | 6.58(3.81-9.74) |
| Lithuania | 13(6-22) | 0.19(0.10-0.31) | 386(219-580) | 6.69(3.93-9.74) |
| Japan | 574(398-779) | 0.11(0.08-0.14) | 13686(9542-18384) | 3.67(2.53-4.92) |
| Russian Federation | 262(140-407) | 0.11(0.06-0.17) | 11387(7034-15986) | 4.75(2.99-6.66) |
| Brunei Darussalam | 0(0-0) | 0.01(0.00-0.02) | 1(0-2) | 0.22(0.02-0.45) |
| Republic of Korea | 143(97-195) | 0.16(0.11-0.21) | 5449(3833-7427) | 5.91(4.16-8.04) |
| New Zealand | 47(32-63) | 0.50(0.34-0.66) | 1078(758-1416) | 12.18(8.60-16.11) |
| Singapore | 1(0-1) | 0.01(0.01-0.01) | 58(35-85) | 0.66(0.40-0.97) |
| Andorra | 0(0-1) | 0.24(0.15-0.34) | 14(9-19) | 8.44(5.64-12.02) |
| Australia | 231(156-312) | 0.42(0.29-0.56) | 5735(3878-7582) | 11.98(8.19-15.84) |
| Belgium | 88(61-119) | 0.28(0.19-0.37) | 2109(1474-2850) | 8.40(5.85-11.58) |
| Denmark | 59(42-79) | 0.42(0.30-0.55) | 1494(1067-1997) | 11.87(8.47-15.74) |
| France | 650(458-872) | 0.32(0.23-0.42) | 14864(10573-19570) | 9.51(6.77-12.66) |
| Austria | 103(73-137) | 0.44(0.32-0.58) | 2828(2021-3719) | 14.28(10.17-18.66) |
| Cyprus | 5(4-7) | 0.31(0.21-0.43) | 135(95-178) | 6.74(4.72-8.95) |
| Greece | 72(49-94) | 0.21(0.15-0.28) | 1756(1177-2326) | 6.75(4.53-9.13) |
| Finland | 37(25-50) | 0.23(0.16-0.30) | 1094(765-1486) | 8.40(5.91-11.29) |
| Ireland | 24(17-33) | 0.28(0.20-0.38) | 644(450-850) | 7.87(5.49-10.51) |
| Germany | 1621(1161-2072) | 0.63(0.46-0.80) | 37312(27186-48220) | 17.71(12.91-23.00) |
| Iceland | 3(2-4) | 0.36(0.25-0.49) | 60(42-80) | 9.70(6.85-12.87) |
| Israel | 10(5-15) | 0.07(0.04-0.10) | 390(236-593) | 3.14(1.91-4.77) |
| Luxembourg | 6(4-7) | 0.44(0.32-0.58) | 128(92-164) | 11.27(8.13-14.37) |
| Netherlands | 160(113-211) | 0.39(0.28-0.51) | 3769(2729-4854) | 10.00(7.23-12.92) |
| Italy | 592(406-790) | 0.28(0.20-0.37) | 14752(10344-20053) | 8.93(6.25-12.18) |
| Portugal | 71(51-91) | 0.22(0.16-0.28) | 2007(1432-2623) | 7.47(5.33-9.79) |
| Sweden | 147(96-209) | 0.50(0.33-0.71) | 3513(2384-4857) | 14.62(10.07-20.09) |
| Malta | 2(1-3) | 0.18(0.12-0.24) | 55(37-74) | 5.32(3.65-7.23) |
| United Kingdom | 545(392-714) | 0.34(0.25-0.44) | 12456(9076-16356) | 8.93(6.47-11.77) |
| Norway | 33(22-45) | 0.26(0.17-0.35) | 789(540-1073) | 7.34(4.98-9.96) |
| Spain | 306(211-409) | 0.22(0.15-0.29) | 8530(6070-11359) | 8.14(5.78-10.78) |
| Switzerland | 49(35-66) | 0.20(0.14-0.26) | 1128(784-1467) | 5.57(3.85-7.29) |
| Argentina | 78(57-104) | 0.13(0.10-0.18) | 2020(1455-2716) | 3.54(2.55-4.75) |
| Uruguay | 11(8-15) | 0.16(0.11-0.21) | 256(180-345) | 4.29(2.97-5.77) |
| Chile | 54(40-70) | 0.20(0.15-0.26) | 1476(1072-1961) | 5.66(4.11-7.54) |
| United States of America | 1671(1127-2239) | 0.25(0.17-0.34) | 52816(37568-69595) | 8.72(6.19-11.47) |
| Bahamas | 0(0-1) | 0.11(0.06-0.18) | 13(7-21) | 3.43(1.65-5.51) |
| Canada | 172(108-238) | 0.20(0.13-0.28) | 5940(3842-8228) | 7.90(5.14-10.89) |
| Antigua and Barbuda | 0(0-0) | 0.13(0.09-0.18) | 4(3-5) | 3.85(2.66-5.21) |
| Barbados | 1(1-1) | 0.17(0.11-0.24) | 25(17-34) | 4.80(3.31-6.51) |
| Belize | 0(0-0) | 0.09(0.06-0.12) | 9(6-13) | 3.10(2.08-4.34) |
| Dominica | 0(0-0) | 0.17(0.11-0.24) | 4(3-5) | 4.82(3.33-6.56) |
| Cuba | 23(15-32) | 0.11(0.07-0.14) | 678(455-928) | 3.37(2.27-4.63) |
| Dominican Republic | 8(5-12) | 0.09(0.06-0.13) | 295(200-419) | 2.97(2.00-4.23) |
| Grenada | 0(0-0) | 0.15(0.11-0.19) | 5(4-7) | 4.49(3.23-6.04) |
| Guyana | 1(0-1) | 0.11(0.08-0.16) | 23(16-32) | 3.70(2.56-5.22) |
| Haiti | 7(4-12) | 0.17(0.09-0.28) | 283(176-403) | 4.61(2.79-6.70) |
| Jamaica | 3(2-4) | 0.08(0.05-0.12) | 84(56-116) | 2.64(1.76-3.65) |
| Saint Lucia | 0(0-1) | 0.22(0.15-0.31) | 14(9-18) | 5.73(3.91-7.73) |
| Saint Vincent and the Grenadines | 0(0-0) | 0.26(0.18-0.35) | 9(6-12) | 6.56(4.66-8.59) |
| Suriname | 0(0-1) | 0.09(0.05-0.13) | 18(12-25) | 2.88(1.88-4.01) |
| Trinidad and Tobago | 2(1-3) | 0.13(0.08-0.18) | 79(53-107) | 4.14(2.78-5.56) |
| Bolivia (Plurinational State of) | 9(5-14) | 0.13(0.08-0.20) | 325(209-465) | 3.84(2.46-5.48) |
| Ecuador | 6(4-9) | 0.04(0.03-0.06) | 302(197-426) | 1.85(1.20-2.62) |
| Peru | 34(21-51) | 0.10(0.06-0.15) | 1340(829-1921) | 4.00(2.47-5.74) |
| Colombia | 28(17-40) | 0.05(0.03-0.07) | 1090(710-1551) | 1.96(1.28-2.80) |
| Costa Rica | 5(3-8) | 0.09(0.06-0.14) | 184(121-256) | 3.33(2.17-4.64) |
| El Salvador | 5(3-7) | 0.07(0.04-0.10) | 149(99-207) | 2.35(1.56-3.26) |
| Guatemala | 4(3-6) | 0.04(0.03-0.07) | 175(116-256) | 1.65(1.09-2.43) |
| Honduras | 4(3-6) | 0.10(0.06-0.15) | 155(101-211) | 2.70(1.72-3.70) |
| Mexico | 126(89-169) | 0.11(0.08-0.15) | 4798(3297-6456) | 3.90(2.70-5.28) |
| Nicaragua | 2(1-3) | 0.06(0.04-0.08) | 112(73-158) | 2.36(1.54-3.36) |
| Panama | 5(3-8) | 0.11(0.07-0.16) | 166(111-225) | 3.70(2.48-5.03) |
| Venezuela (Bolivarian Republic of) | 21(13-30) | 0.08(0.05-0.11) | 833(541-1169) | 2.81(1.80-3.95) |
| Paraguay | 11(8-16) | 0.22(0.15-0.32) | 376(265-525) | 6.66(4.69-9.33) |
| Brazil | 279(205-362) | 0.12(0.09-0.15) | 10370(7351-14066) | 4.17(2.96-5.66) |
| Bahrain | 0(0-0) | 0.01(0.00-0.01) | 2(1-3) | 0.23(0.14-0.33) |
| Algeria | 2(1-3) | 0.01(0.01-0.02) | 68(40-98) | 0.23(0.13-0.34) |
| Iran (Islamic Republic of) | 3(2-5) | 0.01(0.00-0.01) | 141(87-212) | 0.19(0.12-0.29) |
| Egypt | 1(0-1) | 0.00(0.00-0.00) | 41(25-62) | 0.08(0.05-0.11) |
| Iraq | 1(0-1) | 0.00(0.00-0.01) | 22(15-30) | 0.10(0.07-0.15) |
| Jordan | 0(0-0) | 0.00(0.00-0.00) | 6(4-9) | 0.10(0.06-0.15) |
| Lebanon | 1(1-2) | 0.02(0.01-0.03) | 32(19-48) | 0.51(0.31-0.76) |
| Kuwait | 0(0-0) | 0.00(0.00-0.00) | 1(0-1) | 0.02(0.00-0.05) |
| Libya | 0(0-0) | 0.00(0.00-0.00) | 5(3-8) | 0.10(0.05-0.16) |
| Morocco | 0(0-0) | 0.00(0.00-0.00) | 15(9-23) | 0.04(0.02-0.06) |
| Palestine | 0(0-0) | 0.01(0.01-0.02) | 7(4-9) | 0.28(0.18-0.41) |
| Oman | 0(0-0) | 0.00(0.00-0.01) | 3(1-4) | 0.11(0.06-0.18) |
| Qatar | 0(0-0) | 0.00(0.00-0.01) | 2(1-3) | 0.15(0.08-0.23) |
| Syrian Arab Republic | 1(0-1) | 0.01(0.00-0.02) | 23(10-38) | 0.21(0.09-0.35) |
| Saudi Arabia | 0(0-0) | 0.00(0.00-0.00) | 9(1-18) | 0.04(0.00-0.09) |
| Tunisia | 1(1-3) | 0.01(0.01-0.02) | 49(32-70) | 0.39(0.25-0.56) |
| Turkey | 13(8-19) | 0.02(0.01-0.02) | 432(276-594) | 0.48(0.30-0.65) |
| United Arab Emirates | 0(0-0) | 0.02(0.01-0.03) | 30(16-48) | 0.68(0.36-1.05) |
| Yemen | 0(0-0) | 0.00(0.00-0.00) | 11(6-18) | 0.08(0.04-0.14) |
| Bangladesh | 5(0-12) | 0.01(0.00-0.01) | 181(5-374) | 0.14(0.00-0.30) |
| Afghanistan | 0(0-0) | 0.00(0.00-0.00) | 1(0-3) | 0.01(0.00-0.03) |
| Bhutan | 0(0-0) | 0.01(0.00-0.02) | 1(0-3) | 0.23(0.06-0.55) |
| India | 460(293-648) | 0.05(0.03-0.07) | 17569(10811-24477) | 1.61(0.99-2.24) |
| Nepal | 7(2-12) | 0.04(0.01-0.07) | 256(97-441) | 1.21(0.45-2.11) |
| Pakistan | 7(3-12) | 0.01(0.00-0.02) | 317(134-534) | 0.28(0.11-0.48) |
| Angola | 9(6-14) | 0.13(0.08-0.20) | 346(229-488) | 3.49(2.31-4.90) |
| Central African Republic | 1(0-1) | 0.05(0.02-0.10) | 25(10-45) | 1.36(0.55-2.47) |
| Congo | 3(2-5) | 0.23(0.12-0.37) | 106(60-159) | 5.11(2.93-7.79) |
| Equatorial Guinea | 1(0-1) | 0.21(0.12-0.34) | 20(11-30) | 4.93(2.89-7.60) |
| Democratic Republic of the Congo | 14(5-27) | 0.06(0.02-0.12) | 500(186-872) | 1.64(0.61-3.06) |
| Gabon | 2(1-3) | 0.28(0.17-0.41) | 53(34-74) | 6.25(4.02-8.72) |
| Comoros | 0(0-0) | 0.01(0.00-0.02) | 1(0-2) | 0.26(0.08-0.54) |
| Burundi | 3(1-5) | 0.09(0.04-0.17) | 109(60-168) | 2.56(1.39-4.10) |
| Djibouti | 0(0-0) | 0.00(0.00-0.01) | 0(0-1) | 0.07(0.02-0.17) |
| Eritrea | 0(0-1) | 0.02(0.01-0.03) | 12(5-23) | 0.46(0.17-0.89) |
| Ethiopia | 16(7-33) | 0.05(0.02-0.11) | 706(346-1195) | 1.78(0.86-3.07) |
| Kenya | 12(7-20) | 0.08(0.04-0.13) | 511(322-738) | 2.52(1.56-3.65) |
| Madagascar | 3(1-6) | 0.04(0.02-0.08) | 111(49-195) | 1.10(0.48-1.95) |
| Malawi | 3(1-4) | 0.05(0.03-0.09) | 106(63-166) | 1.57(0.91-2.45) |
| Mauritius | 1(1-2) | 0.07(0.04-0.10) | 39(24-58) | 2.18(1.34-3.21) |
| Mozambique | 3(1-5) | 0.04(0.01-0.07) | 108(47-192) | 1.03(0.46-1.83) |
| Rwanda | 4(1-7) | 0.10(0.04-0.18) | 161(86-251) | 2.86(1.49-4.50) |
| Seychelles | 0(0-0) | 0.06(0.04-0.09) | 2(2-3) | 2.10(1.35-3.01) |
| Somalia | 0(0-0) | 0.00(0.00-0.00) | 0(0-0) | 0.00(0.00-0.00) |
| United Republic of Tanzania | 22(12-37) | 0.13(0.07-0.21) | 734(464-1078) | 3.36(2.12-4.95) |
| Uganda | 10(5-16) | 0.10(0.05-0.16) | 411(239-617) | 3.05(1.80-4.61) |
| Zambia | 8(3-15) | 0.17(0.08-0.32) | 233(116-428) | 3.87(1.95-6.95) |
| Botswana | 0(0-1) | 0.05(0.03-0.07) | 23(15-33) | 1.61(1.06-2.32) |
| Lesotho | 0(0-1) | 0.06(0.03-0.10) | 20(11-32) | 1.96(1.02-3.16) |
| Namibia | 2(1-2) | 0.18(0.11-0.27) | 54(33-78) | 4.66(2.75-6.68) |
| South Africa | 41(29-54) | 0.12(0.09-0.17) | 1539(1075-2023) | 3.65(2.57-4.79) |
| Eswatini | 0(0-0) | 0.09(0.05-0.13) | 12(8-17) | 2.56(1.69-3.56) |
| Zimbabwe | 3(2-4) | 0.07(0.04-0.10) | 130(74-189) | 2.16(1.25-3.23) |
| Benin | 1(1-2) | 0.04(0.02-0.07) | 53(25-84) | 1.18(0.56-1.90) |
| Burkina Faso | 13(8-22) | 0.24(0.14-0.39) | 326(210-487) | 4.62(2.94-6.87) |
| Cameroon | 15(9-21) | 0.22(0.13-0.32) | 457(297-633) | 4.76(3.08-6.55) |
| Cabo Verde | 1(0-1) | 0.14(0.08-0.24) | 13(8-20) | 3.16(1.92-4.65) |
| Chad | 2(0-5) | 0.06(0.01-0.14) | 77(20-155) | 1.62(0.41-3.28) |
| Côte d'Ivoire | 12(7-17) | 0.21(0.13-0.31) | 377(237-534) | 4.63(2.91-6.48) |
| Gambia | 1(0-1) | 0.10(0.05-0.15) | 17(10-26) | 2.15(1.21-3.22) |
| Ghana | 10(5-15) | 0.10(0.05-0.17) | 355(195-529) | 2.64(1.46-4.04) |
| Guinea | 1(1-2) | 0.04(0.01-0.06) | 41(19-68) | 0.85(0.38-1.44) |
| Guinea-Bissau | 0(0-1) | 0.10(0.05-0.16) | 12(7-18) | 2.27(1.32-3.38) |
| Liberia | 1(1-2) | 0.10(0.05-0.16) | 40(24-59) | 2.33(1.40-3.57) |
| Mali | 1(1-2) | 0.03(0.01-0.05) | 48(28-74) | 0.73(0.43-1.13) |
| Mauritania | 0(0-0) | 0.00(0.00-0.00) | 0(0-0) | 0.00(0.00-0.00) |
| Niger | 0(0-0) | 0.00(0.00-0.01) | 7(2-17) | 0.11(0.02-0.25) |
| Nigeria | 87(55-128) | 0.17(0.11-0.25) | 2573(1723-3582) | 3.74(2.52-5.21) |
| Sao Tome and Principe | 0(0-0) | 0.16(0.09-0.23) | 3(2-4) | 3.56(2.19-4.99) |
| Senegal | 0(0-1) | 0.01(0.00-0.02) | 15(4-32) | 0.23(0.06-0.48) |
| Sierra Leone | 2(1-3) | 0.08(0.05-0.12) | 65(39-94) | 2.07(1.26-2.99) |
| Togo | 1(1-2) | 0.06(0.03-0.09) | 44(25-69) | 1.43(0.81-2.23) |
| American Samoa | 0(0-0) | 0.01(0.00-0.02) | 0(0-0) | 0.23(0.03-0.61) |
| Bermuda | 0(0-0) | 0.16(0.10-0.22) | 7(5-10) | 5.14(3.53-7.28) |
| Cook Islands | 0(0-0) | 0.23(0.13-0.36) | 2(1-3) | 7.11(4.46-10.07) |
| Greenland | 0(0-0) | 0.35(0.19-0.54) | 7(4-10) | 10.75(6.30-15.78) |
| Guam | 0(0-0) | 0.04(0.01-0.08) | 4(1-8) | 1.98(0.37-3.95) |
| Monaco | 0(0-1) | 0.18(0.00-0.43) | 6(0-14) | 5.79(0.16-12.68) |
| Nauru | 0(0-0) | 0.19(0.09-0.31) | 0(0-0) | 4.85(2.75-6.98) |
| Niue | 0(0-0) | 0.11(0.04-0.18) | 0(0-0) | 3.66(1.48-5.61) |
| Northern Mariana Islands | 0(0-0) | 0.08(0.01-0.18) | 1(0-3) | 2.55(0.27-5.08) |
| Palau | 0(0-0) | 0.05(0.01-0.11) | 0(0-1) | 2.01(0.46-4.10) |
| Puerto Rico | 7(5-10) | 0.08(0.05-0.11) | 219(148-300) | 3.00(2.03-4.07) |
| Saint Kitts and Nevis | 0(0-0) | 0.08(0.00-0.24) | 1(0-4) | 2.14(0.01-6.08) |
| San Marino | 0(0-0) | 0.19(0.00-0.37) | 6(0-11) | 7.15(0.02-12.44) |
| Tokelau | 0(0-0) | 0.08(0.03-0.17) | 0(0-0) | 2.45(1.12-4.32) |
| Tuvalu | 0(0-0) | 0.04(0.02-0.08) | 0(0-0) | 1.37(0.62-2.34) |
| United States Virgin Islands | 0(0-1) | 0.17(0.00-0.35) | 8(0-17) | 4.64(0.13-9.34) |
| South Sudan | 0(0-0) | 0.00(0.00-0.01) | 3(0-7) | 0.07(0.01-0.20) |
| Sudan | 0(0-0) | 0.00(0.00-0.00) | 0(0-0) | 0.00(0.00-0.00) |
| **Countries and  territories** | **Diet high in sodium** | | | |
|  | **Deaths (95%UI)** | **ASMR (95%UI)** | **DALYs (95%UI)** | **ASDR (95%UI)** |
| China | 3129(408-8541) | 0.19(0.02-0.54) | 108958(19945-260666) | 5.43(0.95-13.42) |
| Taiwan (Province of China) | 85(6-226) | 0.18(0.01-0.48) | 1729(108-5052) | 3.85(0.24-11.27) |
| Cambodia | 12(1-35) | 0.18(0.01-0.52) | 435(45-1168) | 4.46(0.40-12.30) |
| Democratic People's Republic of Korea | 59(6-166) | 0.24(0.02-0.73) | 1737(249-4475) | 5.90(0.81-15.75) |
| Lao People's Democratic Republic | 6(0-16) | 0.21(0.01-0.62) | 185(19-513) | 5.15(0.48-14.53) |
| Maldives | 0(0-1) | 0.17(0.01-0.51) | 12(1-33) | 4.41(0.41-12.01) |
| Indonesia | 250(24-693) | 0.20(0.02-0.59) | 9632(1195-26414) | 5.13(0.56-14.40) |
| Malaysia | 39(3-113) | 0.20(0.01-0.59) | 1246(135-3455) | 5.06(0.51-14.20) |
| Philippines | 102(8-299) | 0.19(0.01-0.57) | 3349(359-9126) | 4.96(0.50-13.69) |
| Thailand | 208(13-643) | 0.19(0.01-0.58) | 5144(420-14234) | 4.67(0.38-12.93) |
| Myanmar | 70(5-191) | 0.21(0.01-0.59) | 2103(215-5676) | 5.13(0.47-13.90) |
| Viet Nam | 162(10-483) | 0.23(0.01-0.69) | 4473(409-12281) | 5.27(0.46-14.50) |
| Sri Lanka | 32(2-93) | 0.16(0.01-0.48) | 1077(100-2914) | 4.35(0.38-12.03) |
| Timor-Leste | 1(0-3) | 0.19(0.01-0.55) | 34(3-93) | 4.78(0.44-13.56) |
| Kiribati | 0(0-0) | 0.15(0.01-0.46) | 2(0-6) | 3.93(0.24-11.77) |
| Micronesia (Federated States of) | 0(0-0) | 0.34(0.02-1.03) | 3(0-10) | 6.60(0.43-20.08) |
| Fiji | 1(0-3) | 0.26(0.01-0.79) | 33(3-95) | 5.72(0.42-16.60) |
| Marshall Islands | 0(0-0) | 0.29(0.02-0.87) | 1(0-4) | 6.10(0.45-17.53) |
| Samoa | 0(0-1) | 0.16(0.00-0.55) | 3(0-11) | 2.61(0.02-9.96) |
| Papua New Guinea | 4(0-12) | 0.16(0.01-0.46) | 145(11-424) | 4.10(0.31-11.55) |
| Tonga | 0(0-0) | 0.20(0.01-0.61) | 4(0-11) | 4.89(0.31-14.56) |
| Solomon Islands | 0(0-1) | 0.20(0.01-0.69) | 11(1-32) | 4.57(0.33-14.07) |
| Armenia | 4(0-12) | 0.09(0.00-0.27) | 98(4-321) | 2.26(0.10-7.48) |
| Vanuatu | 0(0-1) | 0.19(0.01-0.56) | 6(1-18) | 4.87(0.36-13.85) |
| Georgia | 11(1-34) | 0.16(0.01-0.50) | 221(10-752) | 3.42(0.14-11.55) |
| Kyrgyzstan | 2(0-7) | 0.07(0.00-0.23) | 74(3-261) | 1.93(0.07-6.62) |
| Azerbaijan | 4(0-12) | 0.06(0.00-0.18) | 157(5-550) | 1.92(0.07-6.39) |
| Kazakhstan | 9(0-30) | 0.08(0.00-0.27) | 313(10-1078) | 2.14(0.07-7.21) |
| Mongolia | 1(0-3) | 0.06(0.00-0.20) | 34(1-114) | 1.93(0.08-6.25) |
| Turkmenistan | 3(0-8) | 0.09(0.00-0.28) | 78(3-275) | 2.38(0.09-8.17) |
| Tajikistan | 1(0-4) | 0.04(0.00-0.12) | 68(2-235) | 1.58(0.06-5.31) |
| Uzbekistan | 8(0-26) | 0.05(0.00-0.16) | 346(12-1215) | 1.68(0.06-5.70) |
| Albania | 12(2-29) | 0.30(0.05-0.75) | 291(54-703) | 6.76(1.28-16.35) |
| Bosnia and Herzegovina | 17(3-40) | 0.27(0.05-0.63) | 432(70-1027) | 6.72(1.09-15.95) |
| Croatia | 23(4-56) | 0.22(0.04-0.54) | 497(89-1201) | 5.05(0.92-12.18) |
| Hungary | 45(9-104) | 0.20(0.04-0.47) | 1275(263-2938) | 6.12(1.27-14.03) |
| Bulgaria | 62(11-149) | 0.42(0.07-1.01) | 1382(262-3306) | 9.07(1.71-21.56) |
| Czechia | 78(14-178) | 0.32(0.06-0.74) | 2267(404-5368) | 9.56(1.72-22.67) |
| Montenegro | 9(2-21) | 1.14(0.19-2.71) | 157(28-362) | 17.76(3.18-40.61) |
| North Macedonia | 10(2-25) | 0.47(0.08-1.17) | 244(44-600) | 8.89(1.59-20.72) |
| Romania | 87(15-199) | 0.21(0.04-0.47) | 1967(360-4605) | 4.86(0.91-11.34) |
| Poland | 205(23-542) | 0.25(0.03-0.67) | 4831(563-13409) | 6.29(0.73-17.51) |
| Serbia | 53(10-124) | 0.30(0.06-0.71) | 1068(206-2511) | 6.10(1.17-14.40) |
| Slovenia | 14(2-32) | 0.24(0.04-0.56) | 309(54-715) | 6.19(1.12-14.34) |
| Belarus | 7(0-35) | 0.04(0.00-0.21) | 186(0-873) | 1.14(0.00-5.38) |
| Slovakia | 35(6-84) | 0.37(0.06-0.89) | 940(167-2211) | 9.65(1.72-22.60) |
| Latvia | 3(0-14) | 0.06(0.00-0.28) | 78(1-314) | 1.82(0.02-7.27) |
| Republic of Moldova | 2(0-12) | 0.04(0.00-0.19) | 69(0-314) | 1.15(0.00-5.19) |
| Ukraine | 33(0-164) | 0.04(0.00-0.20) | 877(1-4118) | 1.11(0.00-5.16) |
| Estonia | 1(0-7) | 0.04(0.00-0.21) | 26(0-144) | 0.88(0.00-4.75) |
| Lithuania | 5(0-23) | 0.08(0.00-0.32) | 117(0-482) | 1.82(0.00-7.38) |
| Japan | 528(30-1509) | 0.09(0.01-0.26) | 10845(713-30963) | 2.54(0.17-7.16) |
| Singapore | 5(0-13) | 0.06(0.00-0.16) | 214(19-582) | 2.51(0.21-6.82) |
| Russian Federation | 219(5-833) | 0.09(0.00-0.34) | 6717(289-23647) | 2.76(0.13-9.61) |
| Brunei Darussalam | 1(0-2) | 0.29(0.02-0.83) | 17(2-43) | 5.98(0.59-16.21) |
| Republic of Korea | 197(25-519) | 0.22(0.03-0.57) | 5429(746-13840) | 5.77(0.79-14.80) |
| New Zealand | 10(0-45) | 0.10(0.00-0.47) | 222(1-989) | 2.49(0.01-10.90) |
| Andorra | 0(0-0) | 0.04(0.00-0.19) | 2(0-10) | 1.35(0.00-6.13) |
| Australia | 28(0-160) | 0.05(0.00-0.29) | 643(0-3363) | 1.35(0.00-6.85) |
| Belgium | 35(0-125) | 0.11(0.00-0.38) | 726(9-2551) | 2.68(0.04-9.34) |
| Denmark | 14(0-61) | 0.10(0.00-0.42) | 340(2-1410) | 2.72(0.02-11.02) |
| France | 145(0-647) | 0.07(0.00-0.30) | 2864(5-12108) | 1.71(0.00-7.24) |
| Austria | 48(1-152) | 0.20(0.00-0.63) | 1149(34-3616) | 5.45(0.17-17.27) |
| Cyprus | 2(0-8) | 0.12(0.00-0.54) | 40(0-168) | 2.12(0.01-9.06) |
| Greece | 25(0-109) | 0.07(0.00-0.30) | 530(2-2321) | 1.90(0.01-8.04) |
| Finland | 12(0-51) | 0.07(0.00-0.31) | 318(2-1282) | 2.36(0.02-9.09) |
| Ireland | 5(0-24) | 0.06(0.00-0.27) | 118(0-531) | 1.42(0.00-6.38) |
| Germany | 311(1-1329) | 0.12(0.00-0.51) | 7156(50-27706) | 3.41(0.03-12.80) |
| Iceland | 1(0-4) | 0.13(0.00-0.51) | 20(0-76) | 3.14(0.03-11.68) |
| Israel | 11(0-48) | 0.08(0.00-0.33) | 340(1-1384) | 2.63(0.01-10.54) |
| Luxembourg | 1(0-6) | 0.10(0.00-0.44) | 26(0-112) | 2.29(0.01-9.58) |
| Netherlands | 36(0-162) | 0.09(0.00-0.39) | 775(2-3392) | 2.04(0.01-8.72) |
| Portugal | 14(0-66) | 0.04(0.00-0.20) | 405(4-1795) | 1.57(0.02-6.56) |
| Italy | 175(2-724) | 0.08(0.00-0.34) | 4272(97-15802) | 2.62(0.08-9.37) |
| Sweden | 47(0-207) | 0.16(0.00-0.70) | 1015(4-4186) | 4.09(0.02-16.33) |
| Malta | 1(0-4) | 0.09(0.00-0.35) | 24(0-92) | 2.30(0.05-8.58) |
| United Kingdom | 141(1-612) | 0.09(0.00-0.37) | 2736(20-11302) | 1.84(0.02-7.54) |
| Norway | 10(0-44) | 0.08(0.00-0.35) | 213(1-889) | 1.94(0.01-8.03) |
| Spain | 38(0-240) | 0.03(0.00-0.17) | 1136(6-5903) | 1.15(0.01-5.56) |
| Argentina | 56(1-196) | 0.09(0.00-0.33) | 1130(18-3884) | 1.94(0.03-6.67) |
| Switzerland | 12(0-53) | 0.05(0.00-0.21) | 254(1-1088) | 1.23(0.01-5.22) |
| Uruguay | 8(0-28) | 0.11(0.00-0.39) | 157(3-550) | 2.46(0.05-8.58) |
| Chile | 26(1-88) | 0.10(0.00-0.33) | 634(15-2128) | 2.41(0.06-8.12) |
| Canada | 71(0-283) | 0.08(0.00-0.33) | 2228(19-8353) | 2.89(0.03-10.63) |
| United States of America | 608(4-2447) | 0.09(0.00-0.37) | 18740(257-71478) | 3.05(0.05-11.45) |
| Bahamas | 0(0-1) | 0.12(0.00-0.48) | 9(0-34) | 2.50(0.01-10.12) |
| Antigua and Barbuda | 0(0-0) | 0.13(0.00-0.54) | 2(0-9) | 2.53(0.01-10.18) |
| Belize | 0(0-1) | 0.10(0.00-0.40) | 6(0-22) | 2.22(0.01-8.97) |
| Barbados | 0(0-2) | 0.07(0.00-0.34) | 8(0-37) | 1.47(0.00-7.12) |
| Dominica | 0(0-0) | 0.16(0.00-0.62) | 2(0-9) | 2.85(0.01-11.57) |
| Cuba | 23(0-91) | 0.10(0.00-0.40) | 456(2-1876) | 2.15(0.01-8.88) |
| Dominican Republic | 10(0-38) | 0.10(0.00-0.41) | 207(1-859) | 2.17(0.01-8.97) |
| Grenada | 0(0-0) | 0.13(0.00-0.57) | 2(0-10) | 2.55(0.01-11.01) |
| Guyana | 0(0-2) | 0.11(0.00-0.45) | 12(0-50) | 2.37(0.01-9.56) |
| Haiti | 4(0-16) | 0.12(0.00-0.44) | 122(0-490) | 2.49(0.01-9.76) |
| Jamaica | 4(0-17) | 0.11(0.00-0.44) | 78(0-316) | 2.31(0.01-9.28) |
| Saint Lucia | 0(0-1) | 0.15(0.00-0.58) | 6(0-24) | 2.66(0.02-10.47) |
| Saint Vincent and the Grenadines | 0(0-1) | 0.16(0.00-0.64) | 4(0-14) | 2.82(0.01-11.11) |
| Suriname | 1(0-2) | 0.10(0.00-0.39) | 13(0-50) | 2.18(0.01-8.69) |
| Trinidad and Tobago | 2(0-6) | 0.09(0.00-0.37) | 41(0-166) | 2.18(0.01-8.88) |
| Bolivia (Plurinational State of) | 10(0-34) | 0.16(0.00-0.54) | 263(4-872) | 3.45(0.05-11.52) |
| Ecuador | 15(0-53) | 0.11(0.00-0.38) | 413(8-1430) | 2.70(0.05-9.31) |
| Peru | 34(1-115) | 0.10(0.00-0.34) | 929(18-3074) | 2.82(0.05-9.30) |
| Colombia | 110(15-290) | 0.19(0.03-0.50) | 2896(395-7739) | 5.18(0.71-13.83) |
| Costa Rica | 8(0-25) | 0.13(0.00-0.44) | 204(9-633) | 3.67(0.17-11.26) |
| El Salvador | 12(0-42) | 0.17(0.01-0.56) | 263(11-838) | 3.95(0.18-12.55) |
| Guatemala | 11(0-39) | 0.14(0.00-0.46) | 340(15-1071) | 3.44(0.14-11.00) |
| Honduras | 10(0-31) | 0.24(0.01-0.75) | 256(9-797) | 4.97(0.19-15.66) |
| Mexico | 114(2-426) | 0.11(0.00-0.40) | 3145(59-11616) | 2.70(0.05-9.94) |
| Nicaragua | 4(0-15) | 0.11(0.00-0.41) | 143(6-467) | 3.27(0.14-10.94) |
| Venezuela (Bolivarian Republic of) | 37(1-127) | 0.14(0.00-0.48) | 1063(53-3223) | 3.77(0.18-11.40) |
| Panama | 7(0-23) | 0.15(0.00-0.49) | 170(7-548) | 3.75(0.16-12.06) |
| Paraguay | 8(0-29) | 0.16(0.00-0.57) | 200(5-694) | 3.72(0.08-12.97) |
| Brazil | 317(8-1057) | 0.14(0.00-0.46) | 8461(234-28429) | 3.49(0.10-11.74) |
| Bahrain | 0(0-0) | 0.02(0.00-0.14) | 3(0-16) | 0.45(0.00-2.92) |
| Algeria | 4(0-30) | 0.02(0.00-0.17) | 125(0-767) | 0.46(0.00-2.98) |
| Iran (Islamic Republic of) | 8(0-56) | 0.01(0.00-0.09) | 264(0-1595) | 0.39(0.00-2.36) |
| Egypt | 5(0-33) | 0.02(0.00-0.11) | 194(0-1124) | 0.42(0.00-2.57) |
| Jordan | 0(0-3) | 0.01(0.00-0.08) | 21(0-124) | 0.35(0.00-2.06) |
| Iraq | 3(0-19) | 0.02(0.00-0.13) | 96(0-555) | 0.49(0.00-2.94) |
| Lebanon | 1(0-6) | 0.01(0.00-0.09) | 23(0-144) | 0.36(0.00-2.23) |
| Kuwait | 1(0-3) | 0.04(0.00-0.17) | 21(0-97) | 0.91(0.00-4.29) |
| Libya | 0(0-3) | 0.01(0.00-0.08) | 16(0-100) | 0.37(0.00-2.33) |
| Morocco | 4(0-29) | 0.02(0.00-0.12) | 127(0-756) | 0.42(0.00-2.59) |
| Palestine | 0(0-2) | 0.02(0.00-0.15) | 8(0-53) | 0.43(0.00-2.86) |
| Oman | 0(0-2) | 0.02(0.00-0.15) | 8(0-47) | 0.51(0.00-3.19) |
| Qatar | 0(0-0) | 0.02(0.00-0.11) | 3(0-16) | 0.42(0.00-2.65) |
| Syrian Arab Republic | 2(0-10) | 0.02(0.00-0.14) | 48(0-287) | 0.45(0.00-2.88) |
| Saudi Arabia | 1(0-8) | 0.01(0.00-0.09) | 58(0-334) | 0.40(0.00-2.39) |
| Turkey | 10(0-68) | 0.01(0.00-0.09) | 224(0-1598) | 0.26(0.00-1.88) |
| Tunisia | 2(0-13) | 0.02(0.00-0.13) | 48(0-293) | 0.40(0.00-2.45) |
| United Arab Emirates | 0(0-1) | 0.02(0.00-0.13) | 13(0-72) | 0.52(0.00-3.18) |
| Yemen | 1(0-9) | 0.02(0.00-0.10) | 44(0-263) | 0.38(0.00-2.44) |
| Bangladesh | 122(1-442) | 0.13(0.00-0.48) | 2857(21-10262) | 2.57(0.02-9.17) |
| Afghanistan | 1(0-6) | 0.01(0.00-0.09) | 29(0-171) | 0.38(0.00-2.32) |
| Bhutan | 1(0-2) | 0.12(0.00-0.45) | 13(0-48) | 2.45(0.02-9.02) |
| India | 559(11-2229) | 0.07(0.00-0.27) | 19438(546-71196) | 1.86(0.04-6.96) |
| Nepal | 15(0-53) | 0.10(0.00-0.38) | 410(4-1475) | 2.24(0.02-8.07) |
| Pakistan | 81(1-297) | 0.12(0.00-0.45) | 2305(33-8087) | 2.64(0.04-9.26) |
| Angola | 4(0-18) | 0.08(0.00-0.34) | 102(0-468) | 1.49(0.00-6.76) |
| Central African Republic | 1(0-3) | 0.07(0.00-0.33) | 16(0-81) | 1.34(0.00-6.24) |
| Congo | 1(0-5) | 0.10(0.00-0.42) | 27(0-127) | 1.70(0.00-7.50) |
| Equatorial Guinea | 0(0-1) | 0.09(0.00-0.40) | 5(0-24) | 1.59(0.00-7.23) |
| Democratic Republic of the Congo | 6(0-35) | 0.03(0.00-0.18) | 153(0-988) | 0.61(0.00-3.86) |
| Gabon | 1(0-3) | 0.12(0.00-0.47) | 13(0-60) | 1.91(0.00-8.44) |
| Comoros | 0(0-1) | 0.11(0.00-0.38) | 9(0-31) | 2.39(0.05-8.05) |
| Burundi | 2(0-8) | 0.09(0.00-0.34) | 75(2-259) | 2.15(0.05-7.71) |
| Djibouti | 0(0-1) | 0.12(0.00-0.41) | 10(0-35) | 2.56(0.06-8.34) |
| Eritrea | 2(0-6) | 0.13(0.00-0.46) | 47(1-162) | 2.68(0.06-9.22) |
| Kenya | 8(0-28) | 0.07(0.00-0.24) | 252(11-874) | 1.53(0.06-5.35) |
| Ethiopia | 21(1-80) | 0.08(0.00-0.29) | 746(26-2530) | 2.16(0.07-7.20) |
| Madagascar | 8(0-29) | 0.16(0.00-0.58) | 238(5-775) | 3.26(0.06-10.88) |
| Malawi | 4(0-13) | 0.09(0.00-0.32) | 127(3-422) | 2.31(0.05-7.68) |
| Mauritius | 3(0-9) | 0.19(0.01-0.57) | 86(8-238) | 4.91(0.42-13.82) |
| Mozambique | 8(0-25) | 0.14(0.00-0.43) | 224(5-758) | 2.87(0.06-9.46) |
| Rwanda | 3(0-12) | 0.10(0.00-0.35) | 107(3-355) | 2.32(0.05-7.76) |
| Seychelles | 0(0-1) | 0.21(0.01-0.65) | 5(0-14) | 4.81(0.36-14.00) |
| Somalia | 2(0-8) | 0.07(0.00-0.29) | 81(2-290) | 2.01(0.05-6.99) |
| United Republic of Tanzania | 24(1-71) | 0.15(0.01-0.44) | 655(41-1883) | 3.31(0.20-9.45) |
| Uganda | 8(0-28) | 0.09(0.00-0.33) | 249(6-832) | 2.27(0.06-7.61) |
| Zambia | 7(0-26) | 0.18(0.00-0.67) | 168(4-617) | 3.51(0.08-12.91) |
| Botswana | 0(0-1) | 0.03(0.00-0.16) | 9(0-44) | 0.73(0.00-3.86) |
| Lesotho | 0(0-1) | 0.03(0.00-0.18) | 7(0-36) | 0.80(0.00-4.18) |
| Namibia | 0(0-2) | 0.03(0.00-0.20) | 9(0-44) | 0.80(0.00-4.13) |
| South Africa | 10(0-52) | 0.03(0.00-0.17) | 291(0-1472) | 0.75(0.00-3.86) |
| Eswatini | 0(0-0) | 0.03(0.00-0.16) | 4(0-18) | 0.84(0.00-4.14) |
| Zimbabwe | 2(0-8) | 0.05(0.00-0.22) | 62(0-279) | 1.19(0.00-5.36) |
| Benin | 2(0-8) | 0.07(0.00-0.28) | 53(0-211) | 1.39(0.00-5.67) |
| Burkina Faso | 4(0-18) | 0.08(0.00-0.34) | 98(0-413) | 1.45(0.00-6.31) |
| Cameroon | 5(0-22) | 0.08(0.00-0.34) | 127(0-553) | 1.48(0.00-6.22) |
| Cabo Verde | 0(0-1) | 0.08(0.00-0.36) | 6(0-25) | 1.41(0.00-6.03) |
| Chad | 2(0-8) | 0.06(0.00-0.26) | 48(0-208) | 1.19(0.00-5.17) |
| Côte d'Ivoire | 4(0-17) | 0.08(0.00-0.33) | 111(0-465) | 1.52(0.00-6.16) |
| Gambia | 0(0-2) | 0.08(0.00-0.35) | 10(0-46) | 1.41(0.00-6.37) |
| Ghana | 8(0-31) | 0.10(0.00-0.37) | 225(1-857) | 1.93(0.01-7.49) |
| Guinea | 2(0-10) | 0.07(0.00-0.29) | 56(0-234) | 1.29(0.00-5.54) |
| Guinea-Bissau | 0(0-1) | 0.08(0.00-0.34) | 6(0-27) | 1.44(0.00-6.47) |
| Liberia | 1(0-3) | 0.07(0.00-0.29) | 19(0-87) | 1.32(0.00-5.95) |
| Mali | 2(0-8) | 0.04(0.00-0.18) | 63(0-278) | 0.99(0.00-4.20) |
| Mauritania | 1(0-5) | 0.08(0.00-0.37) | 24(0-111) | 1.47(0.00-6.79) |
| Niger | 2(0-8) | 0.05(0.00-0.22) | 57(0-250) | 1.02(0.00-4.49) |
| Nigeria | 27(0-126) | 0.05(0.00-0.26) | 687(1-3076) | 1.07(0.00-4.75) |
| Sao Tome and Principe | 0(0-0) | 0.07(0.00-0.33) | 1(0-5) | 1.36(0.00-6.10) |
| Senegal | 3(0-15) | 0.07(0.00-0.33) | 80(0-351) | 1.37(0.00-6.08) |
| Sierra Leone | 1(0-5) | 0.05(0.00-0.24) | 32(0-141) | 1.14(0.00-4.95) |
| Togo | 1(0-5) | 0.07(0.00-0.31) | 33(0-143) | 1.31(0.00-5.76) |
| American Samoa | 0(0-0) | 0.17(0.00-0.59) | 1(0-4) | 3.13(0.04-11.29) |
| Bermuda | 0(0-1) | 0.09(0.00-0.37) | 3(0-12) | 2.04(0.01-8.07) |
| Cook Islands | 0(0-0) | 0.24(0.01-0.73) | 1(0-4) | 5.43(0.38-16.46) |
| Greenland | 0(0-0) | 0.13(0.00-0.54) | 2(0-9) | 3.77(0.02-14.91) |
| Guam | 0(0-1) | 0.08(0.01-0.26) | 8(1-24) | 3.63(0.25-10.84) |
| Monaco | 0(0-0) | 0.07(0.00-0.31) | 2(0-9) | 1.95(0.01-8.01) |
| Nauru | 0(0-0) | 0.41(0.02-1.42) | 0(0-1) | 8.04(0.52-24.85) |
| Niue | 0(0-0) | 0.29(0.02-0.90) | 0(0-0) | 6.10(0.41-17.92) |
| Northern Mariana Islands | 0(0-0) | 0.31(0.02-0.94) | 2(0-7) | 6.44(0.47-19.43) |
| Palau | 0(0-0) | 0.18(0.01-0.55) | 1(0-2) | 4.55(0.29-13.61) |
| Puerto Rico | 8(0-32) | 0.08(0.00-0.32) | 174(1-694) | 1.99(0.01-8.03) |
| Saint Kitts and Nevis | 0(0-0) | 0.15(0.00-0.64) | 1(0-6) | 2.78(0.01-11.38) |
| San Marino | 0(0-0) | 0.05(0.00-0.22) | 1(0-6) | 1.65(0.01-6.83) |
| Tokelau | 0(0-0) | 0.28(0.02-0.86) | 0(0-0) | 5.91(0.40-17.77) |
| Tuvalu | 0(0-0) | 0.28(0.02-0.87) | 1(0-1) | 5.89(0.38-17.46) |
| United States Virgin Islands | 0(0-1) | 0.11(0.00-0.43) | 4(0-16) | 2.17(0.01-8.86) |
| South Sudan | 2(0-7) | 0.09(0.00-0.34) | 62(1-210) | 2.27(0.05-7.71) |
| Sudan | 2(0-12) | 0.01(0.00-0.09) | 65(0-386) | 0.40(0.00-2.43) |
| **Countries and  territories** | **Lead exposure** | | | |
|  | **Deaths (95%UI)** | **ASMR (95%UI)** | **DALYs (95%UI)** | **ASDR (95%UI)** |
| Democratic People's Republic of Korea | 36(-6-96) | 0.16(-0.02-0.24) | 88954(-122-2246) | 3.14(-0.45-8.21) |
| Taiwan (Province of China) | 50(-7-128) | 0.11(-0.02-0.24) | 11954(-144-2891) | 2.46(-0.32-6.44) |
| Indonesia | 173(-29-428) | 0.16(-0.03-0.24) | 56954(-789-14157) | 3.36(-0.49-8.31) |
| Lao People's Democratic Republic | 5(-1-12) | 0.18(-0.03-0.24) | 13954(-17-345) | 3.93(-0.52-10.23) |
| Cambodia | 9(-1-24) | 0.14(-0.02-0.24) | 28954(-36-761) | 3.14(-0.40-8.33) |
| China | 2270(-369-5685) | 0.15(-0.02-0.24) | 56954(-7719-146211) | 3.09(-0.43-8.01) |
| Malaysia | 19(-3-49) | 0.10(-0.02-0.24) | 54954(-69-1394) | 2.32(-0.30-5.94) |
| Myanmar | 52(-7-128) | 0.16(-0.02-0.24) | 13954(-172-3442) | 3.48(-0.45-8.72) |
| Philippines | 43(-6-109) | 0.08(-0.01-0.24) | 12954(-162-3261) | 1.96(-0.25-5.02) |
| Maldives | 0(0-1) | 0.11(-0.01-0.24) | 954(-1-18) | 2.43(-0.30-6.19) |
| Sri Lanka | 11(-1-28) | 0.06(-0.01-0.24) | 31954(-38-822) | 1.33(-0.16-3.40) |
| Thailand | 78(-12-197) | 0.07(-0.01-0.24) | 16954(-226-4441) | 1.55(-0.21-4.05) |
| Timor-Leste | 1(0-2) | 0.18(-0.03-0.24) | 26954(-3-67) | 3.88(-0.52-9.88) |
| Viet Nam | 90(-13-222) | 0.13(-0.02-0.24) | 21954(-278-5462) | 2.65(-0.35-6.74) |
| Kiribati | 0(0-0) | 0.05(-0.01-0.24) | 954(0-2) | 1.23(-0.16-3.32) |
| Micronesia (Federated States of) | 0(0-0) | 0.10(-0.01-0.24) | 954(0-2) | 1.81(-0.25-4.73) |
| Fiji | 0(0-1) | 0.07(-0.01-0.24) | 954(-1-18) | 1.35(-0.18-3.47) |
| Marshall Islands | 0(0-0) | 0.10(-0.01-0.24) | 954(0-1) | 1.93(-0.27-5.05) |
| Samoa | 0(0-0) | 0.07(-0.01-0.24) | 954(0-4) | 1.44(-0.18-3.72) |
| Papua New Guinea | 2(0-4) | 0.07(-0.01-0.24) | 52954(-7-137) | 1.54(-0.22-4.12) |
| Tonga | 0(0-0) | 0.06(-0.01-0.24) | 954(0-3) | 1.28(-0.17-3.38) |
| Solomon Islands | 0(0-1) | 0.14(-0.02-0.24) | 954(-1-16) | 2.82(-0.39-7.39) |
| Armenia | 2(0-6) | 0.05(-0.01-0.24) | 64954(-8-170) | 1.46(-0.18-3.86) |
| Vanuatu | 0(0-0) | 0.10(-0.02-0.24) | 954(0-8) | 2.34(-0.33-6.23) |
| Georgia | 8(-1-21) | 0.13(-0.02-0.24) | 19954(-26-484) | 3.07(-0.41-7.64) |
| Azerbaijan | 2(0-6) | 0.03(-0.01-0.24) | 98954(-12-267) | 1.18(-0.15-3.19) |
| Kyrgyzstan | 2(0-4) | 0.05(-0.01-0.24) | 56954(-7-147) | 1.42(-0.19-3.72) |
| Kazakhstan | 4(-1-11) | 0.04(-0.01-0.24) | 15954(-19-400) | 1.00(-0.13-2.62) |
| Tajikistan | 1(0-3) | 0.03(-0.01-0.24) | 66954(-8-178) | 1.48(-0.19-3.97) |
| Uzbekistan | 4(-1-11) | 0.03(-0.00-0.24) | 21954(-25-557) | 1.00(-0.13-2.66) |
| Mongolia | 1(0-2) | 0.05(-0.01-0.24) | 29954(-4-77) | 1.67(-0.21-4.47) |
| Turkmenistan | 1(0-3) | 0.05(-0.01-0.24) | 45954(-6-117) | 1.34(-0.19-3.49) |
| Albania | 4(-1-10) | 0.11(-0.02-0.24) | 98954(-14-253) | 2.30(-0.32-5.90) |
| Bulgaria | 15(-2-39) | 0.11(-0.01-0.24) | 33954(-42-858) | 2.21(-0.28-5.69) |
| Bosnia and Herzegovina | 7(-1-18) | 0.11(-0.02-0.24) | 16954(-22-449) | 2.61(-0.33-6.90) |
| Czechia | 15(-2-37) | 0.06(-0.01-0.24) | 40954(-54-1049) | 1.71(-0.23-4.45) |
| Croatia | 6(-1-16) | 0.06(-0.01-0.24) | 12954(-18-331) | 1.29(-0.18-3.33) |
| North Macedonia | 3(0-7) | 0.13(-0.02-0.24) | 64954(-9-173) | 2.38(-0.33-6.33) |
| Hungary | 11(-2-30) | 0.05(-0.01-0.24) | 29954(-39-736) | 1.38(-0.19-3.50) |
| Poland | 77(-11-196) | 0.09(-0.01-0.24) | 18954(-242-4843) | 2.43(-0.31-6.28) |
| Montenegro | 2(0-5) | 0.24(-0.04-0.24) | 31954(-4-80) | 3.61(-0.51-9.29) |
| Serbia | 13(-2-34) | 0.08(-0.01-0.24) | 26954(-38-685) | 1.50(-0.21-3.92) |
| Slovenia | 3(0-7) | 0.05(-0.01-0.24) | 58954(-8-146) | 1.14(-0.15-2.92) |
| Romania | 25(-4-61) | 0.06(-0.01-0.24) | 52954(-75-1318) | 1.28(-0.18-3.21) |
| Belarus | 9(-1-24) | 0.06(-0.01-0.24) | 23954(-31-602) | 1.41(-0.19-3.64) |
| Slovakia | 8(-1-20) | 0.08(-0.01-0.24) | 19954(-26-487) | 2.02(-0.27-5.02) |
| Latvia | 2(0-6) | 0.05(-0.01-0.24) | 53954(-7-136) | 1.19(-0.16-3.00) |
| Republic of Moldova | 3(-1-8) | 0.05(-0.01-0.24) | 90954(-13-235) | 1.49(-0.21-3.87) |
| Estonia | 2(0-5) | 0.06(-0.01-0.24) | 40954(-5-99) | 1.26(-0.16-3.16) |
| Ukraine | 35(-5-93) | 0.04(-0.01-0.24) | 80954(-104-2168) | 1.00(-0.13-2.68) |
| Lithuania | 4(-1-9) | 0.05(-0.01-0.24) | 78954(-10-200) | 1.19(-0.16-3.11) |
| Russian Federation | 128(-19-315) | 0.05(-0.01-0.24) | 32954(-408-8330) | 1.32(-0.17-3.40) |
| Japan | 190(-28-484) | 0.03(-0.00-0.24) | 34954(-446-8807) | 0.73(-0.09-1.85) |
| Singapore | 3(0-8) | 0.04(-0.01-0.24) | 11954(-14-305) | 1.34(-0.16-3.65) |
| Australia | 100(-15-250) | 0.18(-0.03-0.24) | 19954(-250-4902) | 3.72(-0.48-9.58) |
| Brunei Darussalam | 0(0-1) | 0.13(-0.02-0.24) | 954(-1-15) | 2.39(-0.31-6.30) |
| Republic of Korea | 79(-12-196) | 0.09(-0.01-0.24) | 19954(-268-5032) | 2.06(-0.29-5.38) |
| New Zealand | 17(-3-44) | 0.18(-0.03-0.24) | 33954(-46-820) | 3.62(-0.50-8.96) |
| Austria | 26(-4-68) | 0.10(-0.02-0.24) | 54954(-76-1381) | 2.50(-0.35-6.38) |
| Andorra | 0(0-0) | 0.05(-0.01-0.24) | 954(0-6) | 1.34(-0.17-3.59) |
| Cyprus | 3(0-8) | 0.22(-0.03-0.24) | 59954(-8-149) | 3.26(-0.48-8.14) |
| Finland | 7(-1-17) | 0.04(-0.01-0.24) | 14954(-20-376) | 1.01(-0.13-2.58) |
| Germany | 222(-32-578) | 0.08(-0.01-0.24) | 43954(-577-11111) | 1.92(-0.25-4.94) |
| Belgium | 45(-7-111) | 0.13(-0.02-0.24) | 84954(-118-2145) | 3.03(-0.41-7.70) |
| Denmark | 14(-2-35) | 0.10(-0.01-0.24) | 28954(-38-731) | 2.18(-0.29-5.51) |
| France | 203(-30-522) | 0.09(-0.01-0.24) | 36954(-498-9350) | 2.03(-0.28-5.32) |
| Iceland | 1(0-2) | 0.12(-0.02-0.24) | 15954(-2-39) | 2.31(-0.31-5.86) |
| Israel | 11(-2-28) | 0.07(-0.01-0.24) | 27954(-38-724) | 2.05(-0.28-5.37) |
| Greece | 40(-6-100) | 0.11(-0.02-0.24) | 77954(-106-1984) | 2.57(-0.34-6.61) |
| Luxembourg | 1(0-3) | 0.10(-0.01-0.24) | 24954(-3-58) | 1.97(-0.27-4.88) |
| Netherlands | 41(-6-106) | 0.10(-0.01-0.24) | 79954(-112-1987) | 2.00(-0.28-5.02) |
| Ireland | 9(-1-22) | 0.10(-0.02-0.24) | 17954(-24-457) | 2.07(-0.28-5.36) |
| Italy | 218(-34-557) | 0.10(-0.02-0.24) | 43954(-579-11319) | 2.33(-0.30-6.18) |
| Malta | 2(0-6) | 0.18(-0.03-0.24) | 46954(-7-118) | 4.13(-0.59-10.54) |
| Portugal | 41(-6-102) | 0.12(-0.02-0.24) | 92954(-133-2350) | 3.09(-0.44-7.83) |
| Sweden | 30(-4-77) | 0.10(-0.01-0.24) | 59954(-78-1556) | 2.27(-0.29-6.02) |
| United Kingdom | 138(-20-347) | 0.08(-0.01-0.24) | 24954(-336-6290) | 1.64(-0.22-4.17) |
| Norway | 12(-2-30) | 0.09(-0.01-0.24) | 21954(-29-564) | 1.88(-0.25-4.91) |
| Spain | 192(-28-491) | 0.13(-0.02-0.24) | 37954(-509-9490) | 3.09(-0.41-7.80) |
| Switzerland | 15(-2-40) | 0.06(-0.01-0.24) | 28954(-39-727) | 1.30(-0.18-3.34) |
| Argentina | 34(-5-84) | 0.06(-0.01-0.24) | 68954(-90-1744) | 1.17(-0.16-2.99) |
| Uruguay | 6(-1-16) | 0.09(-0.01-0.24) | 12954(-16-323) | 1.97(-0.25-5.09) |
| Canada | 50(-7-128) | 0.06(-0.01-0.24) | 12954(-167-3400) | 1.61(-0.21-4.25) |
| Chile | 10(-1-24) | 0.04(-0.01-0.24) | 22954(-29-557) | 0.85(-0.11-2.12) |
| Antigua and Barbuda | 0(0-0) | 0.17(-0.02-0.24) | 954(0-7) | 3.14(-0.43-8.17) |
| United States of America | 543(-82-1388) | 0.08(-0.01-0.24) | 13954(-1801-34336) | 2.11(-0.28-5.41) |
| Barbados | 1(0-2) | 0.13(-0.02-0.24) | 13954(-2-33) | 2.50(-0.32-6.39) |
| Cuba | 45(-7-110) | 0.20(-0.03-0.24) | 92954(-122-2366) | 4.40(-0.57-11.24) |
| Dominican Republic | 24(-4-59) | 0.25(-0.04-0.24) | 52954(-75-1341) | 5.54(-0.79-14.02) |
| Belize | 0(0-1) | 0.12(-0.02-0.24) | 954(-1-18) | 2.79(-0.36-7.19) |
| Bahamas | 0(0-1) | 0.10(-0.02-0.24) | 954(-1-18) | 2.10(-0.29-5.37) |
| Dominica | 0(0-0) | 0.19(-0.03-0.24) | 954(0-7) | 3.47(-0.47-8.83) |
| Guyana | 1(0-2) | 0.22(-0.03-0.24) | 24954(-3-64) | 4.77(-0.65-12.22) |
| Grenada | 0(0-0) | 0.25(-0.04-0.24) | 954(-1-12) | 4.80(-0.63-12.43) |
| Haiti | 14(-2-36) | 0.37(-0.06-0.24) | 39954(-55-995) | 7.79(-1.10-19.87) |
| Jamaica | 8(-1-20) | 0.20(-0.03-0.24) | 14954(-19-360) | 4.20(-0.54-10.62) |
| Saint Lucia | 1(0-2) | 0.28(-0.04-0.24) | 11954(-2-28) | 4.98(-0.70-12.29) |
| Saint Vincent and the Grenadines | 0(0-1) | 0.35(-0.05-0.24) | 954(-1-20) | 6.10(-0.82-15.43) |
| Trinidad and Tobago | 2(0-4) | 0.09(-0.01-0.24) | 34954(-5-91) | 1.87(-0.26-4.87) |
| Suriname | 1(0-3) | 0.19(-0.03-0.24) | 24954(-3-62) | 4.07(-0.60-10.68) |
| Bolivia (Plurinational State of) | 13(-2-36) | 0.21(-0.03-0.24) | 33954(-45-892) | 4.45(-0.60-11.83) |
| Ecuador | 14(-2-34) | 0.10(-0.02-0.24) | 34954(-44-894) | 2.26(-0.29-5.87) |
| Peru | 34(-5-90) | 0.10(-0.01-0.24) | 92954(-119-2413) | 2.80(-0.36-7.31) |
| Colombia | 78(-11-196) | 0.13(-0.02-0.24) | 17954(-217-4291) | 3.06(-0.39-7.64) |
| Costa Rica | 9(-1-21) | 0.15(-0.02-0.24) | 19954(-26-487) | 3.48(-0.46-8.69) |
| El Salvador | 24(-3-59) | 0.31(-0.04-0.24) | 44954(-60-1132) | 6.58(-0.87-16.56) |
| Guatemala | 24(-4-57) | 0.29(-0.04-0.24) | 62954(-84-1585) | 6.50(-0.89-16.46) |
| Honduras | 18(-3-47) | 0.47(-0.07-0.24) | 43954(-61-1071) | 8.85(-1.27-21.78) |
| Nicaragua | 7(-1-17) | 0.19(-0.03-0.24) | 18954(-25-472) | 4.42(-0.60-11.16) |
| Mexico | 206(-33-516) | 0.20(-0.03-0.24) | 51954(-722-12837) | 4.52(-0.64-11.21) |
| Venezuela (Bolivarian Republic of) | 45(-6-113) | 0.17(-0.02-0.24) | 11954(-152-2852) | 4.17(-0.55-10.26) |
| Panama | 8(-1-20) | 0.16(-0.02-0.24) | 16954(-22-410) | 3.61(-0.49-8.98) |
| Brazil | 347(-52-867) | 0.15(-0.02-0.24) | 85954(-1086-21601) | 3.55(-0.45-8.98) |
| Paraguay | 9(-1-21) | 0.17(-0.02-0.24) | 18954(-24-485) | 3.55(-0.45-9.19) |
| Algeria | 39(-5-98) | 0.22(-0.03-0.24) | 81954(-100-2004) | 3.42(-0.44-8.46) |
| Bahrain | 0(0-1) | 0.14(-0.02-0.24) | 10954(-1-25) | 2.25(-0.33-5.98) |
| Egypt | 54(-8-132) | 0.21(-0.03-0.24) | 15954(-202-3985) | 4.25(-0.56-10.44) |
| Iran (Islamic Republic of) | 112(-17-274) | 0.19(-0.03-0.24) | 26954(-350-6540) | 4.04(-0.55-10.12) |
| Iraq | 21(-3-55) | 0.16(-0.02-0.24) | 50954(-66-1260) | 3.03(-0.41-7.55) |
| Jordan | 3(0-8) | 0.09(-0.01-0.24) | 97954(-13-252) | 1.93(-0.27-4.91) |
| Kuwait | 2(0-4) | 0.08(-0.01-0.24) | 34954(-5-87) | 1.62(-0.22-4.18) |
| Lebanon | 6(-1-15) | 0.08(-0.01-0.24) | 10954(-14-277) | 1.59(-0.20-4.16) |
| Libya | 4(-1-10) | 0.10(-0.02-0.24) | 90954(-13-229) | 2.24(-0.32-5.69) |
| Morocco | 29(-5-76) | 0.13(-0.02-0.24) | 64954(-92-1617) | 2.41(-0.35-6.03) |
| Palestine | 3(0-8) | 0.25(-0.04-0.24) | 61954(-9-158) | 3.93(-0.57-10.38) |
| Oman | 1(0-3) | 0.14(-0.02-0.24) | 32954(-5-80) | 2.60(-0.37-6.56) |
| Qatar | 0(0-1) | 0.08(-0.01-0.24) | 954(-1-17) | 1.43(-0.19-3.92) |
| Saudi Arabia | 8(-1-20) | 0.11(-0.02-0.24) | 26954(-36-681) | 2.42(-0.34-6.36) |
| Syrian Arab Republic | 13(-2-35) | 0.20(-0.03-0.24) | 32954(-43-823) | 3.64(-0.51-9.17) |
| Tunisia | 17(-2-43) | 0.17(-0.02-0.24) | 32954(-43-807) | 2.91(-0.39-7.20) |
| Turkey | 73(-11-192) | 0.10(-0.01-0.24) | 13954(-180-3336) | 1.63(-0.22-4.16) |
| United Arab Emirates | 1(0-1) | 0.08(-0.01-0.24) | 27954(-3-70) | 1.74(-0.24-4.44) |
| Yemen | 18(-3-49) | 0.24(-0.04-0.24) | 46954(-71-1180) | 4.76(-0.75-12.13) |
| Bangladesh | 255(-41-662) | 0.29(-0.05-0.24) | 57954(-811-14549) | 5.27(-0.76-13.36) |
| Afghanistan | 11(-2-31) | 0.22(-0.04-0.24) | 31954(-46-814) | 4.61(-0.70-12.06) |
| Bhutan | 1(0-3) | 0.27(-0.04-0.24) | 29954(-4-72) | 5.42(-0.77-13.54) |
| India | 1352(-217-3420) | 0.17(-0.03-0.24) | 39954(-5577-100784) | 4.05(-0.58-10.33) |
| Nepal | 38(-6-102) | 0.27(-0.04-0.24) | 10954(-144-2526) | 5.59(-0.82-14.23) |
| Pakistan | 151(-23-381) | 0.23(-0.04-0.24) | 41954(-569-10202) | 4.88(-0.68-12.00) |
| Angola | 8(-1-21) | 0.15(-0.03-0.24) | 23954(-36-601) | 3.03(-0.47-7.71) |
| Central African Republic | 2(0-4) | 0.16(-0.03-0.24) | 51954(-8-136) | 3.58(-0.57-9.64) |
| Congo | 2(0-4) | 0.13(-0.02-0.24) | 45954(-7-118) | 2.53(-0.38-6.62) |
| Democratic Republic of the Congo | 27(-4-75) | 0.14(-0.02-0.24) | 75954(-117-1985) | 2.98(-0.47-7.72) |
| Equatorial Guinea | 0(0-1) | 0.14(-0.02-0.24) | 954(-1-24) | 2.68(-0.42-7.02) |
| Gabon | 1(0-2) | 0.14(-0.02-0.24) | 19954(-3-48) | 2.54(-0.36-6.60) |
| Burundi | 3(0-9) | 0.13(-0.02-0.24) | 98954(-14-267) | 2.90(-0.42-7.95) |
| Comoros | 0(0-1) | 0.12(-0.02-0.24) | 954(-1-24) | 2.49(-0.36-6.48) |
| Djibouti | 0(0-1) | 0.14(-0.02-0.24) | 11954(-2-28) | 2.80(-0.41-6.99) |
| Eritrea | 2(0-5) | 0.15(-0.02-0.24) | 48954(-7-126) | 2.87(-0.42-7.73) |
| Ethiopia | 37(-5-102) | 0.14(-0.02-0.24) | 11954(-160-3072) | 3.46(-0.49-9.24) |
| Kenya | 11(-2-31) | 0.10(-0.02-0.24) | 34954(-47-890) | 2.20(-0.31-5.78) |
| Madagascar | 9(-1-23) | 0.18(-0.03-0.24) | 23954(-31-587) | 3.36(-0.46-8.50) |
| Malawi | 5(-1-12) | 0.12(-0.02-0.24) | 14954(-21-365) | 2.72(-0.40-6.78) |
| Mauritius | 1(0-3) | 0.08(-0.01-0.24) | 29954(-4-75) | 1.68(-0.22-4.40) |
| Mozambique | 11(-2-29) | 0.20(-0.04-0.24) | 31954(-52-794) | 4.10(-0.70-10.31) |
| Rwanda | 4(0-10) | 0.12(-0.02-0.24) | 11954(-16-294) | 2.59(-0.37-6.79) |
| Seychelles | 0(0-0) | 0.07(-0.01-0.24) | 954(0-4) | 1.45(-0.19-3.80) |
| Somalia | 4(-1-9) | 0.14(-0.02-0.24) | 15954(-24-397) | 3.75(-0.59-9.87) |
| United Republic of Tanzania | 14(-2-38) | 0.09(-0.02-0.24) | 37954(-55-987) | 1.92(-0.29-5.01) |
| Uganda | 9(-1-26) | 0.11(-0.02-0.24) | 28954(-44-734) | 2.64(-0.41-6.81) |
| Zambia | 7(-1-19) | 0.19(-0.03-0.24) | 16954(-24-438) | 3.50(-0.51-9.20) |
| Botswana | 1(0-2) | 0.11(-0.01-0.24) | 27954(-4-70) | 2.51(-0.35-6.44) |
| Lesotho | 1(0-2) | 0.12(-0.02-0.24) | 25954(-3-66) | 3.02(-0.41-7.79) |
| Namibia | 1(0-2) | 0.10(-0.02-0.24) | 23954(-3-59) | 2.24(-0.31-5.71) |
| South Africa | 24(-4-60) | 0.08(-0.01-0.24) | 69954(-88-1836) | 1.86(-0.24-4.87) |
| Zimbabwe | 4(-1-9) | 0.11(-0.02-0.24) | 13954(-17-336) | 2.65(-0.37-6.82) |
| Eswatini | 0(0-1) | 0.11(-0.02-0.24) | 11954(-2-29) | 2.70(-0.37-7.12) |
| Benin | 4(-1-10) | 0.13(-0.02-0.24) | 90954(-12-231) | 2.43(-0.34-6.21) |
| Burkina Faso | 14(-2-38) | 0.27(-0.05-0.24) | 31954(-48-828) | 4.81(-0.75-12.76) |
| Cameroon | 12(-2-31) | 0.19(-0.03-0.24) | 29954(-41-756) | 3.56(-0.49-9.10) |
| Cabo Verde | 1(0-2) | 0.15(-0.02-0.24) | 10954(-1-26) | 2.54(-0.36-6.41) |
| Chad | 6(-1-16) | 0.21(-0.03-0.24) | 16954(-23-415) | 4.20(-0.58-10.51) |
| Côte d'Ivoire | 9(-1-21) | 0.17(-0.02-0.24) | 21954(-29-557) | 3.08(-0.41-7.99) |
| Gambia | 1(0-3) | 0.20(-0.03-0.24) | 25954(-4-63) | 3.55(-0.51-8.93) |
| Ghana | 8(-1-21) | 0.10(-0.01-0.24) | 21954(-28-575) | 1.96(-0.26-5.14) |
| Guinea | 7(-1-18) | 0.20(-0.03-0.24) | 15954(-23-383) | 3.76(-0.54-9.16) |
| Guinea-Bissau | 1(0-1) | 0.20(-0.03-0.24) | 16954(-2-41) | 3.69(-0.54-9.75) |
| Liberia | 2(0-5) | 0.18(-0.03-0.24) | 47954(-7-122) | 3.34(-0.48-8.53) |
| Mali | 6(-1-17) | 0.14(-0.02-0.24) | 20954(-29-524) | 3.26(-0.47-8.09) |
| Mauritania | 2(0-5) | 0.14(-0.02-0.24) | 40954(-5-102) | 2.46(-0.34-6.22) |
| Niger | 7(-1-18) | 0.18(-0.03-0.24) | 21954(-31-534) | 3.88(-0.58-9.90) |
| Nigeria | 51(-9-129) | 0.11(-0.02-0.24) | 12954(-184-3323) | 2.03(-0.30-5.19) |
| Sao Tome and Principe | 0(0-0) | 0.15(-0.03-0.24) | 954(0-6) | 2.76(-0.42-6.99) |
| Senegal | 7(-1-19) | 0.16(-0.03-0.24) | 15954(-22-397) | 2.77(-0.40-7.25) |
| Sierra Leone | 3(0-7) | 0.14(-0.02-0.24) | 78954(-12-201) | 2.79(-0.42-7.30) |
| Togo | 2(0-6) | 0.15(-0.02-0.24) | 66954(-9-167) | 2.77(-0.39-6.95) |
| American Samoa | 0(0-0) | 0.05(-0.01-0.24) | 954(0-1) | 0.94(-0.12-2.45) |
| Bermuda | 0(0-0) | 0.07(-0.01-0.24) | 954(0-6) | 1.48(-0.19-3.87) |
| Cook Islands | 0(0-0) | 0.04(-0.01-0.24) | 954(0-1) | 0.90(-0.12-2.39) |
| Greenland | 0(0-0) | 0.09(-0.01-0.24) | 954(0-3) | 2.16(-0.28-5.65) |
| Guam | 0(0-0) | 0.01(-0.00-0.24) | 954(0-3) | 0.55(-0.07-1.46) |
| Monaco | 0(0-0) | 0.06(-0.01-0.24) | 954(0-5) | 1.48(-0.19-3.87) |
| Nauru | 0(0-0) | 0.09(-0.01-0.24) | 954(0-0) | 1.65(-0.26-4.54) |
| Niue | 0(0-0) | 0.06(-0.01-0.24) | 954(0-0) | 1.09(-0.14-2.80) |
| Northern Mariana Islands | 0(0-0) | 0.05(-0.01-0.24) | 954(0-1) | 0.91(-0.12-2.36) |
| Palau | 0(0-0) | 0.03(-0.00-0.24) | 954(0-0) | 0.70(-0.09-1.78) |
| Puerto Rico | 7(-1-18) | 0.07(-0.01-0.24) | 14954(-19-382) | 1.71(-0.22-4.37) |
| Saint Kitts and Nevis | 0(0-0) | 0.20(-0.03-0.24) | 954(0-5) | 3.52(-0.48-9.09) |
| San Marino | 0(0-0) | 0.04(-0.01-0.24) | 954(0-3) | 1.15(-0.15-3.20) |
| Tokelau | 0(0-0) | 0.07(-0.01-0.24) | 954(0-0) | 1.31(-0.17-3.29) |
| Tuvalu | 0(0-0) | 0.10(-0.01-0.24) | 954(0-0) | 1.97(-0.26-4.95) |
| United States Virgin Islands | 0(0-0) | 0.09(-0.01-0.24) | 954(0-8) | 1.75(-0.23-4.44) |
| South Sudan | 3(0-7) | 0.13(-0.02-0.24) | 75954(-11-193) | 2.85(-0.41-7.16) |
| Sudan | 19(-3-51) | 0.16(-0.03-0.24) | 52954(-75-1306) | 3.55(-0.53-8.78) |
| **Countries and  territories** | **Smoking** | | | |
|  | **Deaths (95%UI)** | **ASMR (95%UI)** | **DALYs (95%UI)** | **ASDR (95%UI)** |
| China | 2682(1472-4093) | 0.16(0.09-0.25) | 113501(63200-171028) | 5.51（3.05-8.26） |
| Taiwan (Province of China) | 59(32-86) | 0.13(0.07-0.18) | 1978(1088-2936) | 4.63（2.56-6.89） |
| Cambodia | 16(9-26) | 0.22(0.12-0.38) | 698(399-1064) | 6.55（3.75-10.14） |
| Democratic People's Republic of Korea | 29(15-44) | 0.10(0.05-0.16) | 1436(802-2205) | 4.32（2.42-6.54） |
| Lao People's Democratic Republic | 7(4-10) | 0.24(0.13-0.38) | 289(166-436) | 7.06（4.15-10.79） |
| Indonesia | 315(179-486) | 0.25(0.13-0.39) | 15629(8887-23308) | 7.43（4.26-11.05） |
| Malaysia | 32(18-50) | 0.16(0.08-0.25) | 1283(758-1976) | 4.92（2.90-7.59） |
| Maldives | 1(0-1) | 0.25(0.13-0.41) | 21(12-32) | 6.85（3.77-10.34） |
| Philippines | 99(58-151) | 0.17(0.10-0.27) | 4321(2586-6364) | 5.62（3.35-8.39） |
| Myanmar | 79(44-122) | 0.23(0.13-0.36) | 2765(1568-4179) | 6.30（3.55-9.72） |
| Thailand | 168(89-265) | 0.15(0.08-0.24) | 5209(2976-7745) | 4.78（2.73-7.13） |
| Sri Lanka | 17(8-29) | 0.08(0.04-0.14) | 757(414-1141) | 2.97（1.62-4.48） |
| Viet Nam | 137(78-217) | 0.18(0.10-0.28) | 5616(3260-8274) | 5.82（3.38-8.70） |
| Timor-Leste | 1(1-2) | 0.19(0.10-0.30) | 45(24-69) | 5.88（3.26-8.95） |
| Kiribati | 0(0-0) | 0.21(0.12-0.34) | 7(4-11) | 9.35（5.49-14.03） |
| Fiji | 1(1-2) | 0.15(0.08-0.24) | 54(30-81) | 6.42（3.66-9.56） |
| Micronesia (Federated States of) | 0(0-0) | 0.31(0.17-0.46) | 10(6-14) | 11.52（6.61-17.05） |
| Samoa | 0(0-0) | 0.19(0.10-0.30) | 11(6-16) | 7.19（4.08-10.93） |
| Marshall Islands | 0(0-0) | 0.18(0.09-0.27) | 2(1-4) | 6.10（3.36-9.44） |
| Papua New Guinea | 6(3-9) | 0.11(0.05-0.18) | 312(169-480) | 4.99（2.68-7.67） |
| Tonga | 0(0-0) | 0.22(0.11-0.35) | 6(3-9) | 7.70（4.23-11.64） |
| Armenia | 5(3-7) | 0.11(0.07-0.17) | 234(138-351) | 5.36（3.13-8.12） |
| Solomon Islands | 1(0-1) | 0.19(0.09-0.31) | 29(17-46) | 7.74（4.44-12.01） |
| Vanuatu | 0(0-0) | 0.12(0.06-0.20) | 9(5-14) | 4.94（2.73-7.68） |
| Georgia | 14(8-21) | 0.23(0.13-0.33) | 451(271-654) | 7.71（4.72-11.03） |
| Azerbaijan | 5(3-9) | 0.07(0.04-0.10) | 421(228-663) | 4.05（2.22-6.38） |
| Kazakhstan | 8(5-12) | 0.05(0.03-0.07) | 661(356-1012) | 3.46（1.88-5.37） |
| Mongolia | 1(1-2) | 0.07(0.04-0.10) | 110(61-165) | 4.49（2.52-6.76） |
| Kyrgyzstan | 3(2-5) | 0.08(0.05-0.12) | 204(118-310) | 4.13（2.39-6.33） |
| Turkmenistan | 2(1-2) | 0.04(0.02-0.07) | 100(55-156) | 2.36（1.30-3.62） |
| Tajikistan | 1(0-1) | 0.02(0.01-0.03) | 95(48-152) | 1.56（0.81-2.49） |
| Bosnia and Herzegovina | 12(6-19) | 0.19(0.10-0.30) | 418(233-632) | 6.75（3.74-10.14） |
| Uzbekistan | 6(3-9) | 0.03(0.01-0.04) | 591(313-913) | 2.10（1.13-3.22） |
| Croatia | 15(8-23) | 0.15(0.08-0.23) | 424(246-644) | 4.76（2.79-7.19） |
| Albania | 9(5-15) | 0.23(0.12-0.39) | 332(185-509) | 7.67（4.26-11.66） |
| Hungary | 17(10-26) | 0.08(0.05-0.13) | 795(440-1187) | 4.39（2.46-6.55） |
| Bulgaria | 23(13-34) | 0.16(0.09-0.24) | 857(494-1251) | 6.56（3.81-9.48） |
| Czechia | 33(19-49) | 0.14(0.08-0.21) | 1536(881-2291) | 7.28（4.18-10.77） |
| Montenegro | 5(3-8) | 0.58(0.32-0.90) | 143(83-216) | 14.57（8.48-22.11） |
| Romania | 31(18-46) | 0.08(0.04-0.12) | 1241(711-1909) | 3.54（2.04-5.39） |
| North Macedonia | 6(3-11) | 0.26(0.13-0.42) | 245(133-379) | 7.80（4.40-11.85） |
| Poland | 99(59-147) | 0.13(0.08-0.19) | 4666(2666-7228) | 6.72（3.87-10.32） |
| Serbia | 23(13-36) | 0.13(0.07-0.21) | 776(444-1177) | 4.83（2.81-7.35） |
| Slovenia | 3(2-5) | 0.06(0.03-0.09) | 147(82-226) | 3.60（2.00-5.48） |
| Belarus | 19(11-27) | 0.11(0.07-0.17) | 922(531-1392) | 5.84（3.35-8.86） |
| Slovakia | 14(8-22) | 0.15(0.08-0.23) | 646(364-957) | 6.84（3.87-10.16） |
| Latvia | 4(2-6) | 0.10(0.06-0.15) | 204(118-303) | 5.88（3.41-8.72） |
| Republic of Moldova | 5(3-7) | 0.08(0.05-0.11) | 262(154-396) | 4.49（2.64-6.81） |
| Ukraine | 63(34-97) | 0.08(0.04-0.12) | 3007(1681-4664) | 4.03（2.25-6.25） |
| Estonia | 3(2-5) | 0.11(0.07-0.17) | 132(76-200) | 5.40（3.10-8.17） |
| Lithuania | 7(4-11) | 0.11(0.06-0.16) | 278(160-425) | 5.28（3.01-8.01） |
| Russian Federation | 244(150-355) | 0.10(0.06-0.15) | 12986(7783-19300) | 5.53（3.29-8.28） |
| Japan | 341(195-513) | 0.08(0.04-0.11) | 10598(6053-16484) | 3.46（1.97-5.25） |
| Brunei Darussalam | 0(0-1) | 0.16(0.09-0.25) | 18(10-26) | 4.89（2.75-7.40） |
| Republic of Korea | 129(68-208) | 0.14(0.07-0.23) | 5103(2946-7752) | 5.67（3.30-8.56） |
| New Zealand | 20(11-30) | 0.21(0.12-0.33) | 574(328-875) | 6.85（3.91-10.47） |
| Singapore | 2(1-3) | 0.02(0.01-0.03) | 160(86-255) | 1.81（0.97-2.89） |
| Australia | 52(28-81) | 0.10(0.06-0.15) | 1894(1052-2978) | 4.44（2.45-6.92） |
| Andorra | 0(0-0) | 0.10(0.05-0.17) | 8(5-13) | 5.40（2.89-8.38） |
| Belgium | 31(16-47) | 0.11(0.06-0.16) | 1085(630-1692) | 5.04（2.91-7.87） |
| Denmark | 35(20-53) | 0.26(0.14-0.38) | 1092(625-1644) | 9.27（5.37-14.01） |
| France | 191(104-299) | 0.11(0.06-0.17) | 6825(3879-10614) | 5.36（3.02-8.25） |
| Austria | 35(19-55) | 0.17(0.09-0.26) | 1467(863-2254) | 8.52（5.01-13.07） |
| Cyprus | 3(2-5) | 0.17(0.10-0.27) | 111(63-168) | 5.48（3.09-8.32） |
| Finland | 13(7-20) | 0.09(0.05-0.14) | 567(321-894) | 5.14（2.97-8.01） |
| Greece | 54(30-81) | 0.17(0.10-0.26) | 1651(930-2552) | 7.22（4.09-10.98） |
| Ireland | 13(7-21) | 0.16(0.08-0.24) | 403(226-618) | 5.07（2.82-7.75） |
| Germany | 469(264-716) | 0.20(0.11-0.30) | 15930(9138-24424) | 8.93（5.16-13.64） |
| Iceland | 1(1-2) | 0.15(0.09-0.24) | 31(17-48) | 5.37（2.99-8.20） |
| Israel | 15(8-23) | 0.11(0.06-0.17) | 767(430-1171) | 6.44（3.60-9.87） |
| Luxembourg | 2(1-3) | 0.15(0.08-0.23) | 60(34-92) | 5.65（3.26-8.68） |
| Italy | 181(100-281) | 0.10(0.06-0.15) | 6702(3707-10608) | 4.84（2.69-7.75） |
| Portugal | 13(7-20) | 0.05(0.03-0.07) | 658(377-1015) | 3.12（1.77-4.80） |
| Netherlands | 84(48-128) | 0.21(0.12-0.32) | 2425(1388-3736) | 6.84（3.90-10.51） |
| Malta | 1(0-1) | 0.08(0.05-0.13) | 33(19-50) | 3.70（2.14-5.62） |
| Norway | 15(8-24) | 0.12(0.07-0.20) | 465(260-750) | 4.76（2.69-7.53） |
| United Kingdom | 279(157-441) | 0.18(0.10-0.28) | 7377(4184-11288) | 5.62（3.19-8.69） |
| Argentina | 40(23-62) | 0.07(0.04-0.11) | 1611(936-2436) | 2.94（1.72-4.46） |
| Sweden | 67(36-108) | 0.24(0.13-0.38) | 2056(1109-3234) | 9.27（5.01-14.63） |
| Spain | 104(58-157) | 0.08(0.05-0.12) | 4787(2789-7287) | 5.35（3.13-8.19） |
| Switzerland | 24(14-37) | 0.10(0.06-0.15) | 683(390-1061) | 3.70（2.09-5.74） |
| Chile | 15(9-22) | 0.06(0.03-0.08) | 741(429-1132) | 2.97（1.72-4.56） |
| Uruguay | 5(3-8) | 0.08(0.05-0.12) | 188(107-289) | 3.62（2.07-5.49） |
| United States of America | 940(536-1439) | 0.15(0.09-0.23) | 36861(21247-55370) | 6.37（3.70-9.49） |
| Bahamas | 0(0-0) | 0.09(0.05-0.14) | 12(7-19) | 2.99（1.64-4.64） |
| Canada | 96(53-149) | 0.12(0.07-0.18) | 3865(2137-6163) | 5.40（3.01-8.46） |
| Belize | 0(0-0) | 0.10(0.05-0.16) | 12(7-18) | 3.92（2.19-6.14） |
| Antigua and Barbuda | 0(0-0) | 0.09(0.05-0.14) | 3(2-5) | 2.95（1.65-4.64） |
| Dominica | 0(0-0) | 0.10(0.05-0.16) | 3(2-4) | 3.29（1.84-5.18） |
| Grenada | 0(0-0) | 0.08(0.04-0.13) | 4(2-5) | 3.04（1.66-4.56） |
| Barbados | 0(0-1) | 0.08(0.04-0.12) | 12(7-19) | 2.32（1.32-3.68） |
| Haiti | 3(2-6) | 0.07(0.03-0.12) | 163(87-271) | 2.26（1.21-3.66） |
| Cuba | 28(15-43) | 0.13(0.07-0.20) | 1072(602-1638) | 5.45（3.07-8.26） |
| Dominican Republic | 19(9-32) | 0.20(0.10-0.34) | 554(305-884) | 5.66（3.10-9.09） |
| Guyana | 0(0-1) | 0.09(0.05-0.15) | 23(13-35) | 3.47（1.96-5.36） |
| Saint Lucia | 0(0-0) | 0.10(0.05-0.15) | 8(4-12) | 3.22（1.77-4.92） |
| Jamaica | 3(2-5) | 0.10(0.05-0.16) | 114(65-178) | 3.63（2.07-5.64） |
| Saint Vincent and the Grenadines | 0(0-0) | 0.11(0.06-0.17) | 5(3-8) | 3.50（2.02-5.33） |
| Suriname | 1(0-1) | 0.11(0.06-0.18) | 29(16-44) | 4.45（2.54-6.78） |
| Trinidad and Tobago | 2(1-3) | 0.10(0.05-0.16) | 81(44-125) | 4.22（2.28-6.54） |
| Bolivia (Plurinational State of) | 7(4-12) | 0.09(0.05-0.16) | 305(166-467) | 3.40（1.85-5.20） |
| Ecuador | 10(5-15) | 0.06(0.04-0.10) | 432(241-663) | 2.66（1.50-4.08） |
| Peru | 21(11-36) | 0.06(0.03-0.11) | 893(484-1389) | 2.65（1.44-4.14） |
| Colombia | 32(17-51) | 0.06(0.03-0.09) | 1346(765-2129) | 2.42（1.38-3.85） |
| Costa Rica | 5(3-8) | 0.09(0.05-0.14) | 192(106-302) | 3.49（1.91-5.50） |
| El Salvador | 4(2-6) | 0.05(0.03-0.09) | 139(79-214) | 2.26（1.29-3.47） |
| Guatemala | 6(3-10) | 0.07(0.04-0.10) | 267(149-412) | 2.50（1.39-3.85） |
| Honduras | 7(4-11) | 0.15(0.08-0.24) | 282(160-432) | 4.67（2.63-7.15） |
| Mexico | 113(67-166) | 0.10(0.06-0.15) | 4597(2616-7141) | 3.68（2.11-5.75） |
| Nicaragua | 3(1-4) | 0.07(0.04-0.11) | 143(80-227) | 2.95（1.66-4.71） |
| Panama | 3(2-5) | 0.07(0.04-0.12) | 114(62-182) | 2.56（1.39-4.09） |
| Venezuela (Bolivarian Republic of) | 25(13-39) | 0.09(0.05-0.14) | 1086(604-1682) | 3.61（2.01-5.66） |
| Paraguay | 14(7-23) | 0.27(0.13-0.45) | 443(243-687) | 7.89（4.31-12.27） |
| Brazil | 286(167-431) | 0.12(0.07-0.18) | 12118(6858-18679) | 4.82（2.73-7.45） |
| Bahrain | 0(0-1) | 0.11(0.05-0.21) | 22(12-36) | 2.84（1.54-4.63） |
| Algeria | 28(15-46) | 0.16(0.08-0.27) | 918(514-1406) | 3.35（1.86-5.24） |
| Iran (Islamic Republic of) | 32(17-50) | 0.05(0.03-0.08) | 1487(822-2315) | 1.95（1.08-3.03） |
| Egypt | 42(23-62) | 0.14(0.07-0.21) | 1920(1102-2904) | 3.81（2.15-5.98） |
| Iraq | 27(15-44) | 0.17(0.09-0.29) | 969(548-1464) | 4.50（2.54-6.89） |
| Jordan | 5(3-8) | 0.11(0.06-0.18) | 257(145-389) | 3.79（2.19-5.81） |
| Kuwait | 2(1-3) | 0.09(0.05-0.14) | 78(44-121) | 2.66（1.48-4.08） |
| Lebanon | 11(6-17) | 0.16(0.09-0.25) | 289(161-446) | 4.61（2.58-7.03） |
| Libya | 2(1-4) | 0.06(0.03-0.11) | 122(69-193) | 2.39（1.29-3.71） |
| Morocco | 11(6-17) | 0.04(0.02-0.06) | 503(281-774) | 1.47（0.83-2.26） |
| Palestine | 2(1-3) | 0.14(0.07-0.24) | 77(44-115) | 3.49（2.05-5.35） |
| Qatar | 0(0-0) | 0.04(0.02-0.08) | 23(11-40) | 1.73（0.89-2.86） |
| Oman | 1(0-1) | 0.07(0.03-0.11) | 39(21-62) | 1.87（1.01-2.86） |
| Saudi Arabia | 5(3-8) | 0.03(0.02-0.05) | 371(201-568) | 1.50（0.84-2.25） |
| Syrian Arab Republic | 10(5-16) | 0.13(0.06-0.21) | 383(211-585) | 3.32（1.83-5.12） |
| Turkey | 65(36-100) | 0.08(0.04-0.12) | 2325(1321-3446) | 2.53（1.43-3.74） |
| Tunisia | 17(9-28) | 0.16(0.08-0.27) | 470(260-714) | 3.86（2.12-5.89） |
| Yemen | 10(5-17) | 0.10(0.05-0.18) | 392(213-640) | 3.02（1.58-4.86） |
| United Arab Emirates | 1(0-1) | 0.06(0.03-0.10) | 101(49-169) | 2.14（1.19-3.39） |
| Bangladesh | 248(132-405) | 0.26(0.14-0.43) | 7511(4184-11403) | 6.17（3.46-9.51） |
| Afghanistan | 3(1-5) | 0.04(0.02-0.08) | 133(73-216) | 1.39（0.73-2.18） |
| Bhutan | 0(0-1) | 0.08(0.04-0.14) | 12(6-19) | 2.13（1.12-3.43） |
| India | 943(530-1430) | 0.11(0.06-0.16) | 34761(19795-53021) | 3.22（1.85-4.91） |
| Nepal | 38(19-62) | 0.24(0.12-0.41) | 1213(679-1923) | 6.11（3.44-9.77） |
| Pakistan | 106(54-167) | 0.13(0.06-0.21) | 4471(2465-6676) | 4.18（2.33-6.45） |
| Angola | 7(4-11) | 0.08(0.04-0.13) | 308(169-482) | 2.65（1.41-4.03） |
| Congo | 2(1-3) | 0.09(0.05-0.15) | 71(40-108) | 2.75（1.52-4.24） |
| Central African Republic | 1(0-2) | 0.05(0.02-0.09) | 40(20-67) | 1.72（0.88-2.83） |
| Democratic Republic of the Congo | 13(6-21) | 0.04(0.02-0.07) | 583(309-933) | 1.49（0.80-2.39） |
| Equatorial Guinea | 0(0-0) | 0.07(0.03-0.12) | 12(6-19) | 2.31（1.18-3.71） |
| Gabon | 1(0-1) | 0.07(0.04-0.11) | 23(13-35) | 2.19（1.16-3.36） |
| Burundi | 2(1-3) | 0.04(0.02-0.08) | 83(42-139) | 1.66（0.85-2.84） |
| Comoros | 0(0-1) | 0.10(0.04-0.20) | 12(6-20) | 2.62（1.26-4.50） |
| Djibouti | 1(0-1) | 0.15(0.07-0.25) | 25(14-40) | 4.38（2.39-6.86） |
| Eritrea | 1(0-1) | 0.03(0.01-0.06) | 33(17-53) | 1.05（0.54-1.70） |
| Ethiopia | 8(3-15) | 0.02(0.01-0.04) | 417(213-671) | 0.96（0.50-1.56） |
| Kenya | 9(4-15) | 0.06(0.03-0.10) | 430(235-695) | 1.96（1.06-3.17） |
| Madagascar | 6(3-10) | 0.09(0.04-0.15) | 239(127-378) | 2.21（1.18-3.52） |
| Malawi | 6(3-10) | 0.11(0.05-0.20) | 232(122-368) | 3.42（1.79-5.58） |
| Mauritius | 2(1-3) | 0.15(0.09-0.22) | 91(54-139) | 5.08（3.03-7.72） |
| Mozambique | 8(4-13) | 0.10(0.05-0.16) | 306(166-494) | 2.83（1.54-4.57） |
| Rwanda | 7(2-13) | 0.18(0.06-0.37) | 264(128-438) | 4.99（2.41-8.79） |
| Seychelles | 0(0-0) | 0.16(0.09-0.26) | 6(3-9) | 5.36（3.14-8.40） |
| Somalia | 2(0-4) | 0.04(0.01-0.08) | 102(48-184) | 1.65（0.80-2.93） |
| United Republic of Tanzania | 19(9-34) | 0.10(0.04-0.19) | 792(402-1297) | 3.21（1.62-5.26） |
| Uganda | 6(2-10) | 0.05(0.02-0.10) | 255(134-422) | 1.81（0.93-3.00） |
| Zambia | 8(3-15) | 0.16(0.07-0.32) | 252(111-464) | 3.99（1.80-7.22） |
| Botswana | 1(1-2) | 0.10(0.05-0.16) | 51(29-79) | 3.59（2.00-5.51） |
| Lesotho | 1(0-2) | 0.12(0.06-0.21) | 37(20-59) | 3.69（2.03-5.94） |
| Namibia | 1(1-2) | 0.17(0.09-0.29) | 49(27-75) | 4.29（2.39-6.65） |
| South Africa | 29(17-43) | 0.08(0.04-0.12) | 1387(821-2076) | 2.94（1.75-4.42） |
| Eswatini | 0(0-0) | 0.07(0.03-0.12) | 11(6-17) | 2.10（1.13-3.34） |
| Zimbabwe | 5(3-8) | 0.11(0.06-0.19) | 235(135-358) | 3.74（2.10-5.71） |
| Benin | 1(0-1) | 0.02(0.01-0.04) | 43(23-70) | 0.87（0.46-1.41） |
| Burkina Faso | 2(1-4) | 0.04(0.02-0.06) | 100(52-164) | 1.12（0.56-1.80） |
| Cameroon | 4(2-6) | 0.05(0.02-0.08) | 156(86-244) | 1.31（0.70-2.09） |
| Cabo Verde | 0(0-0) | 0.03(0.02-0.05) | 5(3-8) | 1.05（0.59-1.70） |
| Chad | 2(1-3) | 0.05(0.02-0.10) | 77(42-126) | 1.52（0.81-2.52） |
| Côte d'Ivoire | 4(2-6) | 0.05(0.03-0.09) | 162(85-250) | 1.58（0.81-2.47） |
| Gambia | 0(0-1) | 0.05(0.03-0.09) | 16(9-24) | 1.67（0.92-2.61） |
| Ghana | 5(3-9) | 0.06(0.03-0.10) | 209(118-333) | 1.51（0.82-2.46） |
| Guinea | 2(1-3) | 0.06(0.03-0.09) | 81(43-130) | 1.58（0.82-2.54） |
| Guinea-Bissau | 0(0-0) | 0.03(0.02-0.05) | 7(4-11) | 1.00（0.51-1.59） |
| Liberia | 0(0-1) | 0.03(0.02-0.05) | 25(14-40) | 1.17（0.61-1.86） |
| Mali | 3(1-5) | 0.06(0.03-0.11) | 132(71-210) | 1.81（0.97-2.90） |
| Mauritania | 1(0-1) | 0.04(0.02-0.07) | 30(16-47) | 1.42（0.77-2.23） |
| Niger | 1(1-3) | 0.04(0.01-0.07) | 55(27-92) | 0.88（0.41-1.50） |
| Nigeria | 11(5-18) | 0.02(0.01-0.03) | 527(274-834) | 0.61（0.32-0.96） |
| Sao Tome and Principe | 0(0-0) | 0.02(0.01-0.04) | 1(1-2) | 0.95（0.50-1.52） |
| Senegal | 2(1-3) | 0.03(0.01-0.04) | 83(45-134) | 1.05（0.58-1.68） |
| Sierra Leone | 1(1-2) | 0.05(0.02-0.08) | 68(37-107) | 1.81（0.97-2.85） |
| Togo | 1(1-2) | 0.06(0.03-0.09) | 61(33-97) | 1.72（0.94-2.72） |
| American Samoa | 0(0-0) | 0.18(0.10-0.28) | 4(2-6) | 7.57（4.41-11.23） |
| Bermuda | 0(0-0) | 0.08(0.04-0.13) | 5(3-7) | 3.43（1.90-5.39） |
| Cook Islands | 0(0-0) | 0.16(0.08-0.25) | 2(1-2) | 6.27（3.50-9.81） |
| Greenland | 0(0-0) | 0.34(0.19-0.51) | 9(5-14) | 13.06（7.44-19.52） |
| Guam | 0(0-0) | 0.08(0.05-0.12) | 10(6-15) | 4.97（2.82-7.45） |
| Monaco | 0(0-0) | 0.12(0.06-0.19) | 5(3-8) | 5.37（2.97-8.54） |
| Nauru | 0(0-0) | 0.36(0.18-0.58) | 1(0-1) | 12.14（6.94-17.81） |
| Niue | 0(0-0) | 0.15(0.08-0.24) | 0(0-0) | 6.05（3.44-9.02） |
| Northern Mariana Islands | 0(0-0) | 0.21(0.11-0.31) | 4(3-7) | 7.62（4.41-11.13） |
| Palau | 0(0-0) | 0.09(0.05-0.13) | 1(1-1) | 4.14（2.39-6.22） |
| Puerto Rico | 7(3-11) | 0.08(0.04-0.12) | 226(123-358) | 3.28（1.80-5.06） |
| Saint Kitts and Nevis | 0(0-0) | 0.08(0.04-0.13) | 2(1-2) | 2.35（1.32-3.74） |
| San Marino | 0(0-0) | 0.07(0.03-0.12) | 3(2-4) | 3.94（2.10-6.32） |
| Tokelau | 0(0-0) | 0.18(0.09-0.30) | 0(0-0) | 6.78（3.81-10.60） |
| Tuvalu | 0(0-0) | 0.22(0.12-0.33) | 1(1-1) | 8.34（4.94-12.28） |
| United States Virgin Islands | 0(0-0) | 0.06(0.03-0.10) | 4(2-7) | 2.40（1.33-3.75） |
| South Sudan | 2(1-3) | 0.07(0.03-0.13) | 83(43-137) | 2.23（1.13-3.73） |
| Sudan | 7(4-12) | 0.05(0.03-0.09) | 302(163-478) | 1.70（0.91-2.66） |
